# Crystallographic workshops – a primer and perspective from Whitworth University’s Summer Crystallography Institute

**DOI:** 10.1107/S2056989026000939

**Published:** 2026-02-03

**Authors:** Kraig A. Wheeler

**Affiliations:** a Department of Chemistry Whitworth University 300 W Hawthorne Road Spokane WA 99521 USA; Harvard University, USA

**Keywords:** crystallographic education, on-site workshops

## Abstract

Crystallographic workshops provide participants with a wide range of foundational knowledge and vital practical skills. They build technical and soft skills, bridge theory and practice, and provide avenues for mentorship and career growth. This short perspective article highlights the activities of recent Summer Crystallography Institutes held at Whitworth University and provides additional discussion of key workshop components, including philosophy, objectives, strategies, planning, timeline, schedule, and funding.

## Abstract 2

Whitworth University’s recent Summer Crystallography Institutes offer important insights into training and engaging the next generation of structural scientists in a workshop setting.

## Introduction

*Human Curiosity, the urge to know, is a powerful force and is perhaps the best secret weapon of all in the struggle to unravel the workings of the natural world*. Aaron Klug

*The great advantage of X-ray analysis as a method of chemical structure analysis is its power to show some totally unexpected and surprising structure with, at the same time, complete certainty*. Dorothy Hodgkin

The above quotes from Klug and Hodgkin nicely capture the hallmarks of the discovery process and motivation for crystallographic training (Klug, 1982 ▸; Hodgkin, 1994 ▸). There is something distinctly captivating and emboldening about realizing the mysteries of chemical systems at the atomic level using X-ray diffraction data. Whether it be the most basic and foundational chemical principles or the complex self-assembly of macromolecules, experiencing such structural insights for the first time can be gripping, often accompanied by a desire for more. Why such fascination? Simply stated, where other analytical techniques may disappoint or falter, X-ray diffraction makes three-dimensional atomic structures familiar, like family photos, and accessible to diverse audiences, yielding results with the potential for novelty and a deeper understanding of the connection between structure and function. Fascination with the inherent beauty of crystals dates back to ancient times (Garcia-Ruiz, 2021 ▸), and it is no surprise that many scientific theories and guesses have found support in crystals and X-ray diffraction, underscoring the power of crystallography to uncover and clarify chemical and structural truths (Hasnain, 2015 ▸).

How the crystallographic community best trains current and the next generation of structural scientists has been a topic of discussion for some time, and, as expected, different strategies to crystallographic education are essential for providing a multi-front approach to training (Berry & Guzei, 2026 ▸; Bou-Nader *et al.*, 2025 ▸; Irmer, 2025 ▸; Zheng *et al.*, 2018 ▸; Garcia-Ruiz *et al.*, 2015 ▸). Training experiences in a workshop setting provide one avenue that has proven immensely useful for developing participants in focused areas of crystallographic education. While the titles of these activities may differ (*e.g.*, workshops, summer schools, symposia, or institutes), their mission and activities often follow a similar path, sharing in the high-impact practice of bringing together experts and novices for active engagement around common goals. The term *workshop* is used throughout this article to refer to any short-term, interactive, often hands-on exchange in which participants actively engage in scientific activities to learn and build skills. When done right, those involved go home transformed, with a greater appreciation and connection to the discipline and the participants, and with enhanced abilities to approach a variety of structural challenges on their own.

This contribution to the *Education and Outreach* section of *Acta Crystallographica Section E* describes the 2021–2025 Summer Crystallographic Institutes (SCI) hosted by Whitworth University (WU), with offerings that give some practical insights for those who wish to coordinate similar activities. Additionally, SCI lecture materials and practice crystallographic datasets of various levels are also included in the supporting information to assist those interested in crystallographic training. While several organizational and operational elements presented are specific to Whitworth’s SCI’s mission and objectives, the experiences gained, and many aspects of the planning and implementation processes (see Table 1 ▸) should benefit those seeking to organize a crystallography-focused workshop. The following discussion provides details on these recent SCI activities and some additional thoughts on best practices of organizing and executing workshops.

## Workshop philosophy and objectives

Developing a working philosophy in the first year of planning is essential to the success of any workshop. How you prioritize and perceive the various aspects of the training culture and expected outcomes should ultimately inform the organization of the workshop. The goal of such a philosophy statement should be to provide a clear, inspiring picture of a workshop’s roadmap, align efforts around common goals, define a workshop’s long-term impact and purpose, and even help to attract financial support. Expect to spend considerable time developing your philosophy, as it will provide the organizational framework for building other workshop activities. Though the philosophy and objectives presented below are unique to Whitworth’s SCI, they should offer some insight into the planning process for workshops and the various areas to consider for a successful workshop.

The philosophy of WU’s Summer Crystallographic Institute (SCI) is to provide quality instruction to a broad demographic base of participants in the methods and rewards of small molecule single-crystal X-ray crystallography as a tool for routine characterization. This four-day workshop will be distinguished by targeting junior scholars and their faculty advisors who wish to become better informed consumers of crystallographic data, and by exploring ways in which crystallography can have a greater impact on instructional and research programs.

This philosophy and many of the objectives were shared with participants through a variety of practices, including professional organization websites and listservs, the workshop website, email communications, and other materials provided before and during the workshop.

Specific objectives of the SCI:

Learn by hearing and seeing

· Provide well placed demonstrations and formal discussions of foundational concepts in crystallographic theory.

· Establish a curriculum that supports diverse learning styles and prepares the next generation of structural scientists.

· Engage all participants in the crystallographic process from start to finish.

Learn by interacting

· Engage participants with a full range of practical experiences – *e.g.*, crystal growth to data preparation for publication.

· Allocate *Flex Time* as a significant component for participants to explore and learn various aspects of the crystallographic process.

· Engage research groups with the crystallographic process using their samples.

· Communicate the crystallographic process using data from participant samples.

· Organize meaningful networking opportunities.

Workshop resources

· Provide content-friendly workshop materials that are centrally located and easily accessible to all participants. The SCI utilized a cloud storage option to ensure and simplify access to workshop materials.

· Use single-vendor hardware and software to reduce unnecessary distractions in the workshop.

· Limit participant costs *via* workshop support. This aspect was essential to the SCI’s success, given the potential resource constraints of the home institutions of the target audience.

· Include instructors proficient in the workshop’s hardware and software. For 20–25 participants, we found four instructors ideal: one managed the X-ray laboratory, and the others served as workshop lecturers and mentors. Local students (2–4) also provided essential help with crystal growth experiments and individual assistance with the crystal structure solution and refinement processes.

· Provide a unified set of software and software guides.

· Develop 30–40 practice datasets of varying difficulty and chemical class.

## Target audience

Because participants are central to the operation and success of any workshop, the early planning stages should consider the demographics of the science community and potential attendees. Will the target audience consist of seasoned practitioners, novices in the field, or people with a blend of scientific and crystallographic abilities? Identifying the workshop target audience will help guide every aspect of workshop preparation, including curriculum, resources, and activities.

The initial focus of the SCI was to train the next generation of scientists, with particular attention to undergraduate students and their research advisors. Experience from the first year of the SCI revealed the importance of organizational flexibility. Because of SCI’s under-enrollment among the targeted undergraduate audience and significant interest from nearby PhD-granting research-intensive universities, the SCI quickly broadened its reach to include these other science groups as participants. This change proved transformational for the SCI and its participants, with the benefits mostly evident in informal interactions during the workshop. For instance, (i) several meaningful collaborations were established among the participants and instructors, and (ii) younger attendees with sights on furthering their careers received important information and guidance about graduate school and industrial positions from the more senior participants.

It is worth noting that while the SCI demographic broadened, the workshop retained its initial vision of training users with little to no experience with crystallographic processes and of bringing research groups together to work on common structural problems, using samples prepared in their research laboratories. This target group typically does not attend other crystallographic meetings or workshops and is thus highly suitable to participate in Whitworth’s SCI. It is no surprise that this demographic lacked significant participation in crystallographic workshops from just a few decades ago. However, with the advent of more user-friendly software and hardware, the process of data collection to data manipulation is more accessible than ever. The benefits of these technological advances have been tangible and immediate for the structural sciences, and, as expected, crystallography workshops have developed into learning communities able to reach a wider range of participants and address a broader range of topics.

**Figure 1:**
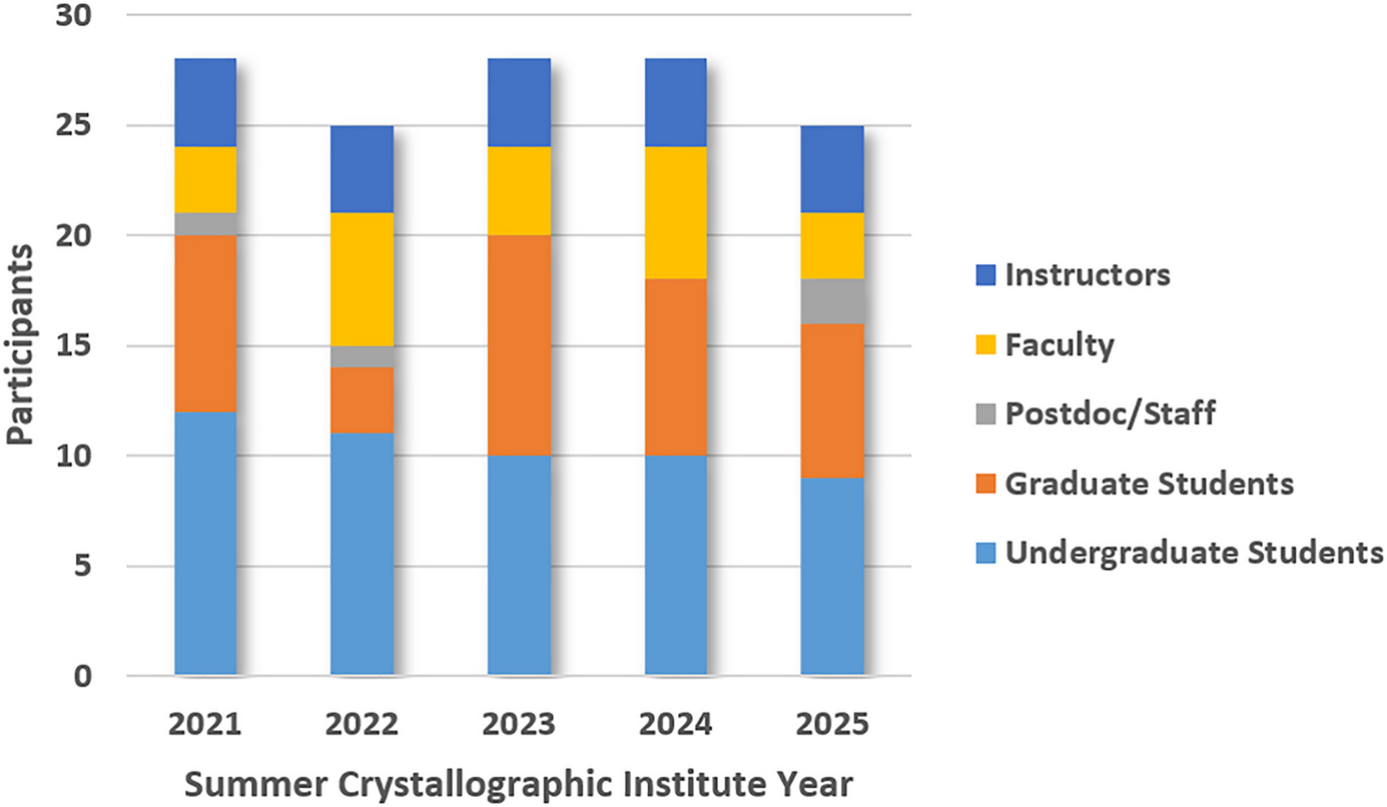
Participant numbers and demographic distribution for Whitworth’s Summer Crystallographic Institute from 2021–2025.

Fig. 1 ▸ provides details of the SCI demographic for each workshop year. Attendees and instructors ranged from 25 to 28 (40:60 ratio of female and male participants), with the majority being undergraduate students (9–12). Though fewer in number, those in the graduate student, faculty, and staff categories were consistent participants who contributed immensely to the workshop culture. University and national laboratory staff contributed 0–2 participants each year.

## Strategies for crystallographic workshops

How a workshop delivers its training and various activities is vital to how participants will engage and retain information about the crystallographic process. Closely connected to information delivery is workshop duration, because the amount of scheduled time will dictate the types and levels of information a workshop can deliver. Because the SCI was designed as a four-day event (two full days and two half-day sessions), careful consideration was given to the intended workshop outcomes in light of the target audience. Due to constraints in the SCI program’s timeframe, the overall approach and several content areas of the crystallographic process were either reduced or excluded from the schedule. Given the workshop’s limited time and the likely inexperience of the attendees with the crystallographic process, all formal lectures began with this level of understanding and progressed to more content-rich material. As such, the SCI did not formally cover the areas of crystal screening, crystal twinning, data collections, powder X-ray diffraction, and space-group determinations. However, the SCI strategy emphasized what is needed to get the job done, what information can be obtained, how to determine what is reliable and what is not, and how to prepare data for publication and presentations. All participants were involved with the crystallographic process from start to finish.

*If we knew all the answers there would be no point in carrying out scientific research. Because we do not, it is stimulating, exciting, challenging*. Kathleen Lonsdale

The goals of a scientific workshop can be immense, as they often introduce participants to new theories, bridge theory and practice, and offer new strategies to answer complex problems. Why do people participate in these day-long or longer activities? Because, as the above quote from Kathleen Lonsdale describes, there is an inherent need to know more, to learn more, to investigate and innovate the unknown, where much excitement and purpose can follow discovery (Lonsdale, 1964 ▸). Effective workshop strategies catalyze this discovery process for their participants. The differences between workshop strategies can be immense, so formulating these details early in the planning stage is one key to success. For the SCI, three areas emerged as important factors during the early strategy planning: cost-reduction measures, hardware use, and participant engagement.

### Cost-reduction measures

The initial desire to keep program costs low was in part to attract those from primarily undergraduate institutions and research groups lacking the funding often found at PhD research-intensive institutions. To reduce the financial barriers for participants, the SCI provided daily meals and scholarships to help reduce participant travel expenses. Information about these scholarships was available on the workshop website, and informal applications were reviewed at the attendees’ request, with reimbursements handled by the host institution after the workshop. It should be said that campuses like Whitworth University can offer ideal settings for workshops. Not only is the geographic setting nature-rich and inspiring, but the administrative structures also enable lower-cost events that may not be available at institutions with business models that target on-site conferences as a source of revenue. Because of this benefit, the significant expenses for the SCI were meals and participant reimbursements, not infrastructure.

### Workshop hardware and facilities

One crucial consideration for any crystallographic workshop is access to laboratory space for crystal growth experiments and a modern in-house X-ray diffractometer. Without these resources, the workshop would be very different for the participant, focusing on discussions of crystallography concepts and workshop-provided datasets, with no exposure to instrument use or sample preparation. The SCI utilized Whitworth’s X-ray facility, equipped with a dual-source diffractometer, microscopes, and teaching technology, all located near the workshop learning area. Participants were granted access to this facility and encouraged to work with the instructors to gain more practical experience with X-ray diffraction experiments and processes.

On-site instrumentation also provides the host institution with opportunities to further serve the scientific community through collaborations established during the workshop. The experience gained with the instrumentation and the relationships developed offer valuable avenues for participants to reconnect after the workshop. This strategy can boost the facility’s productivity and help support instrument maintenance and facility costs.

An alternative strategy invites vendors to share and ship their equipment to the workshop setting. This approach has occurred at larger, more established workshops where the opportunity to showcase their equipment to key audiences exists. The benefit of this model is that participants can be exposed to a wide range of state-of-the-art instrumentation and learn about the advantages of each instrument. The SCI limited the equipment to a single modern dual-source diffractometer and a single vendor. This vendor provided a senior applications specialist to manage the SCI’s X-ray laboratory during the workshop. Because of the diffractometer’s data-collection speed and the facility’s efficiency, this setup effectively served the 21–24 SCI attendees. Also, unwanted distractions caused by software and hardware variances, often encountered at multi-vendor workshops, were eliminated using this model.

### Participant engagement

*A crystal is like a class of children arranged for drill, but standing at ease, so that while the class as a whole has regularity both in time and space, each individual child is a little fidgety!* Kathleen Lonsdale

This quote by Lonsdale is well placed in this section (Lonsdale & DuMond, 1950 ▸). How the workshop approaches the degree of *fidgety* in crystal structures relates to the prior experience of the attendees with the crystallographic process. Novices need access to easy-entry crystallographic concepts and data, while those with more experience require more advanced material and attention. As such, developing formal lectures on crystallographic content can be challenging given the range of this participant experience. The SCI approached this issue by providing low- to mid-level formal crystallographic instruction, with the intent of addressing more advanced topics with attendees on a need-to-know basis. This method of delivering instruction was effective, allowing those less experienced in crystallography to focus on and reexamine the material. At the same time, the other, more experienced attendees could explore additional aspects of the crystallographic process with the instructors’ help; many of these important discussions arose from the structural challenges encountered in datasets from the participant samples.

Dedicated time for these discussions, reviewing lecture material, and crystal growth experiments was scheduled in the SCI as *Flex Time* (Section 6). The sizable set of practice datasets made available *via* the workshop’s cloud storage space provided another essential aspect of *Flex Time*. Each structure included an instruction file and reflection data, a chemical diagram, and a short description of any crystallographic anomaly. This collection of structures accommodated the learning needs of a wide range of participants. In addition to being chemically diverse, these practice datasets varied in difficulty, incorporating missed-atom assignments, chemical connectivity, and varying levels of disorder.

The last day of the SCI was dedicated to participant presentations. Though this activity may seem less weighty than other workshop components, it proved vital to participants’ learning, as they showcased their new crystallographic knowledge and skills. These ten-minute presentations consisted of a description of a crystal structure(s), preferably data acquired at the SCI, emphasizing the stepwise process from crystal structure determination to space-group assignment to crystal packing features. While these presentations began with a few introductory slides to provide scientific context, participants were asked to focus primarily on crystallography to strengthen their workshop experience and engagement. Because preparation for these presentations began days in advance, developing meaningful content for these presentations served as an important training exercise for the participants.

## Planning timeline

Many difficulties in workshop planning can be avoided by creating a detailed, workable action list. The flow of planning begins many months before the actual workshop and includes tasks such as sharing intentions, assembling materials, enlisting instructors and financial supporters, and communicating effectively with participants. The job becomes less daunting when broken down into small, manageable backstage tasks spread over many months. Enlisting others (campus staff and administration, instructors) with specific assignments can also provide considerable help. Also, having a firm grasp of the workshop to-do list will ensure, or at least increase your chances, of a successful workshop.

The planning assignments provided in Table 1 ▸ have emerged from several years of refining Whitworth’s SCI. While some planning details are specific to our workshop, many of the principles outlined in the table should apply and transfer to other related workshop events.

Lastly, though not listed in Table 1 ▸, follow-up activities should be considered an essential part of workshop operation. Collecting feedback from participants through a questionnaire and using this information to reflect and learn will help inform necessary changes for the next workshop. At this same time, work to thank and celebrate with those involved. Recognize and show gratitude for the participants, instructors, and supporters.

## Workshop schedules

**Figure 2:**
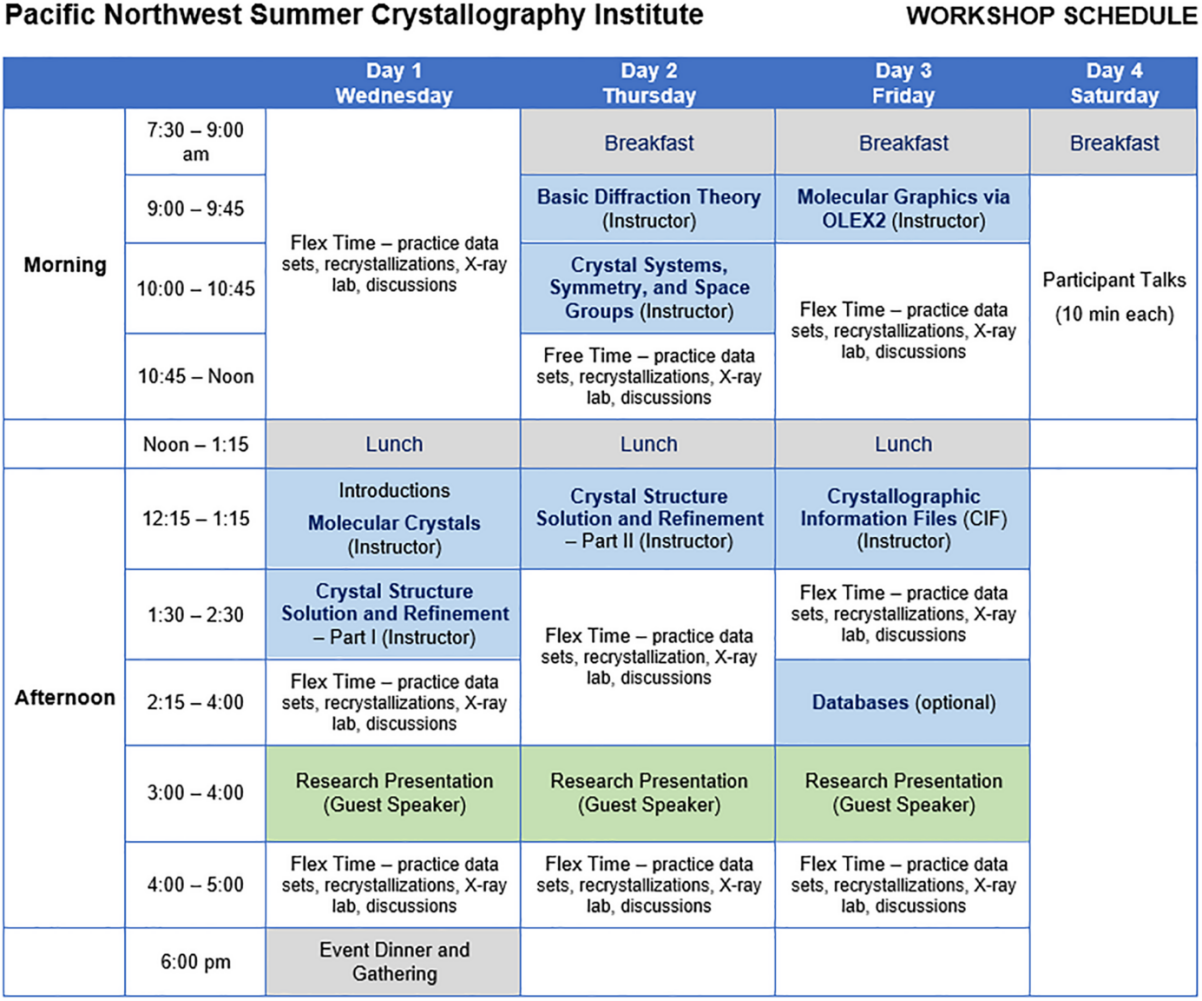
Typical workshop schedule for Whitworth’s Summer Crystallography Institute.

Fig. 2 ▸ provides the details of a typical SCI schedule. Several key features of this timetable include the start and finish of the workshop occurring at noon. This time is strategic due to participants’ travel considerations and because it meets the needs of most local participants, those from bordering states, and attendees who need to travel by air. More importantly, because of the reduced SCI schedule, participants who arrived early (generally, many attendees) could begin crystal growth experiments and data collections, and troubleshoot any software installation issues before the official start of the workshop. It should be noted that while participants were instructed to bring diffraction-ready samples, a subset each year arrived with materials that required recrystallization. Though beyond the general scope of the SCI, for these cases, laboratory space and resources were made available near the X-ray facility.

Two meals (breakfast and lunch) were provided by the SCI on most days, with the noon meal intended as a working lunch in the workshop space to make the most of available time.

In addition to the seven planned formal lectures (*i.e.*, crystal growth, crystal structure solution and refinement I and II), more workshop time was dedicated to *Flex Time*. There always seems to be a need for organizers to fill every available space with content; however, many studies show that learners work best in these flexible learning spaces, where they are given opportunities to engage with the material to establish specialized skills and understanding (Papaioannou *et al.*, 2023 ▸; Laranjeira & Teixeira, 2025 ▸; Wong *et al.*, 2024 ▸; Freeman *et al.*, 2014 ▸; Zhoc *et al.*, 2019 ▸). Experience from the SCI supports this notion that participants need time and space to promote active learning. We found that these unplanned moments provided a key to the workshop’s success. Participants used this time to its fullest by focusing on the various aspects of their samples (*e.g*., crystal growth and structure refinements), interacting with instructors and other participants, and determining and refining crystal structures from the workshop’s numerous datasets. *Flex Time* also provided opportunities to foster community, where individuals from different backgrounds combined efforts on structural questions, ultimately contributing to a constructive learning environment.

In addition to the participants’ natural motivation to work on samples from their laboratory, they also anticipated giving a presentation on the last day of the workshop. These short presentations, described in more detail in Section 4.3, were an essential part of the schedule, providing participants with opportunities to give a detailed discussion of the crystallographic process for a sample(s) determined at the SCI.

## Workshop support

Successful scientific workshops often require support from others. This support comes in many forms, including in-house and external, and financial and in-kind. To determine the level of workshop need, a thorough consideration of workshop planning (Section 5) is essential to gain insight into the organizational framework and the support needed to achieve success. Some support may be considered in-kind, including supplies for the operational aspects of a workshop and an X-ray facility. Other sponsorship, such as financial support, is critical for covering workshop expenses and ensuring diverse participation by covering travel, lodging, and registration costs (Table 1 ▸). The SCI participants accessed the workshop website to register, select on-campus housing options, and provide electronic payment.

The two extremes of covering workshop expenses are charging attendees or seeking full sponsorship. The SCI combined these two approaches to ensure the workshop’s sustainability and that participants incurred minimal costs. This model is not new and requires a sizeable effort to gain financial workshop support. While much has been written about targeting key stakeholders and sales pitches that show impact by providing value (Pallikaraki, 2025 ▸; Kelwig, 2019 ▸), fundraizing simplifies to starting early and leveraging the organizer’s network. Look close and far. There may be opportunities at your home institution for in-kind and financial support, as workshops can benefit local students and scientists, showcase ongoing activities, and enhance the organization’s reputation and visibility. If targeting companies and other agencies, be aware of their fiscal year, as this may dictate their disbursements for workshop support. Organizers should start activity in this area at least nine months before the workshop (Table 1 ▸). In addition to a proactive approach to securing support, the projected workshop budget should inform the time commitment needed for fundraizing. Targeting a broad section of potential funders is important. Federal agencies (or corresponding international agencies), professional organizations, private programs and foundations, corporate funders, public charities, and local scientific sections should be considered and possibly pursued when seeking workshop funding. Also, insights from other successful workshops and from those with funding experience can be helpful resources in securing workshop support.

## Supplementary Material

Supporting information file. DOI: 10.1107/S2056989026000939/oi2032sup2.zip

## Figures and Tables

**Table 1:** Workshop planning activities and timeline

Number of months before workshop	Planning Activities
9	**Contact** financial supporters and instructors (assign duties), share intentions with campus conference services (determine housing and meal costs), and other campus administrators, and potential event dinner sites and caterers. **Create** the workshop title, mission, and dates, contact information for potential participants, a website with workshop description (or update the existing website), promotional materials, and cloud-based storage to serve as the workshop resource center. Also, set up an internal account(s) for participant payments and external support deposits. **Reserve** campus spaces for the workshop and the event dinner, and arrange caterer services. Sign the necessary agreements.
6	**Contact** potential participants, agencies with promotional materials (*e.g*., listserves, vendors, and guilds), and additional financial supporters. **Create** meal plans *via* conference services; website, including registration, campus housing, and payment options; cloud-based storage resources with schedule and logistics (list of local restaurants and sites, campus map, software guides, off-campus housing options).
3-4	**Review** workshop applications. **Contact** all applicants to inform them of the workshop participant decision, financial supporters to assist with transferring funds to a campus account, and instructors and participants to share the cloud-based resource site and schedule. **Create** additional cloud-based resources (*e.g*., practice datasets, participant to-do list, software guides and installation instructions, lecture slide decks, parking passes), a dietary restrictions survey, and other workshop details. **Share** cloud space and workshop materials with participants and instructors.
1 week	**Contact** participants with a final communication. **Create** instructor and participant name tags, required university forms, and X-ray facility forms.

## Data Availability

SCI lecture presentation slide decks and 32 practice datasets (ins and *hkl* files) of varying difficulty are available in the supporting information.
