# Supplementary material for: Crystallographic workshops – a primer and perspective from Whitworth University’s Summer Crystallography Institute
Source: Acta Crystallogr E Crystallogr Commun. 2026 Feb 3;82(Pt 3):313–9. doi: 10.1107/S2056989026000939 (PMC12961664; doi:10.1107/S2056989026000939)
Supplement: Supplementary file 1 [file e-82-00313-sup2.zip › Lectures/SCI_Crystal_Growth.pptx]

## Slide 1
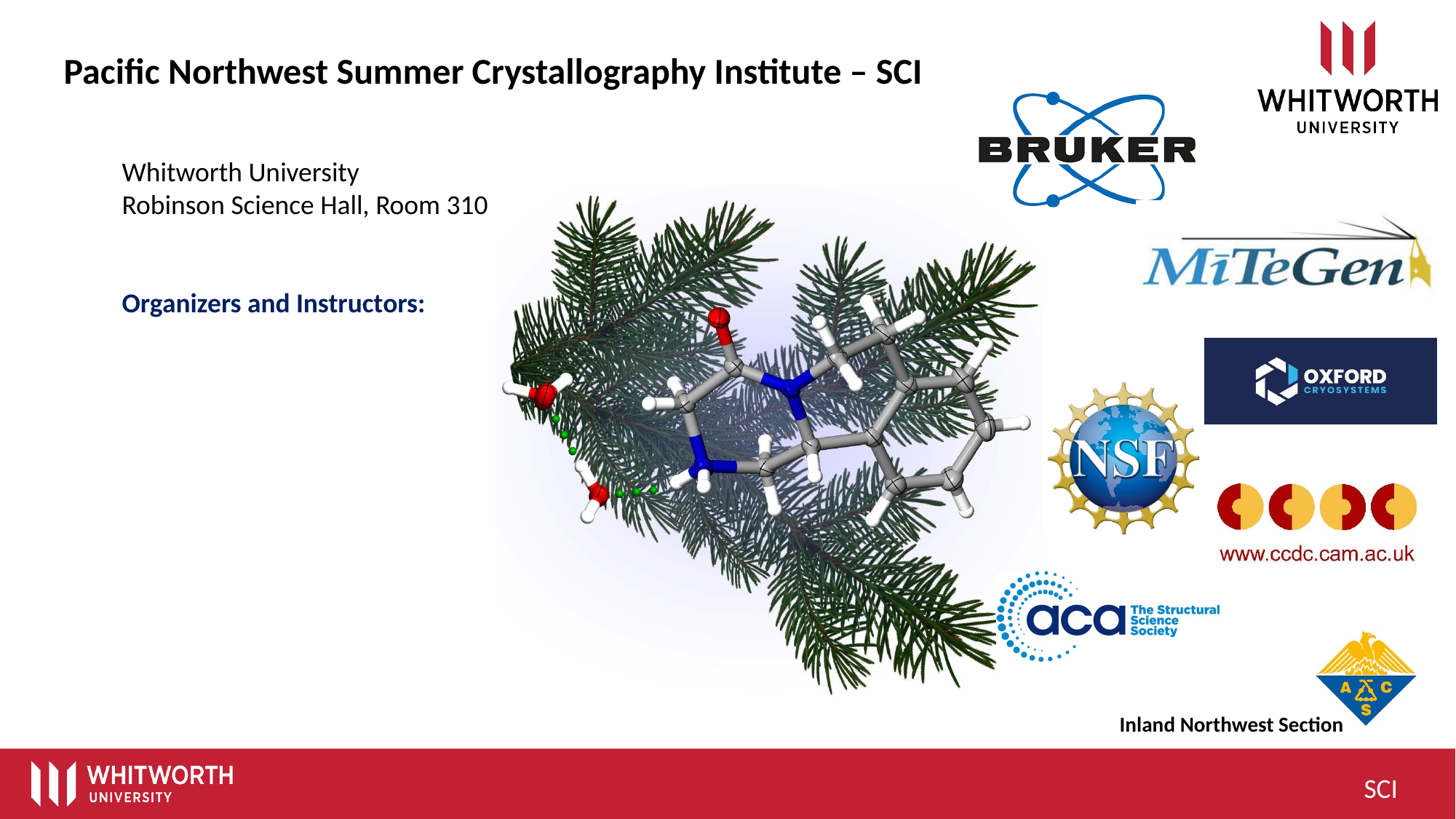

Pacific Northwest Summer Crystallography Institute – SCI
Whitworth University
Robinson Science Hall, Room 310
Organizers and Instructors:
Inland Northwest Section
SCI

## Slide 2
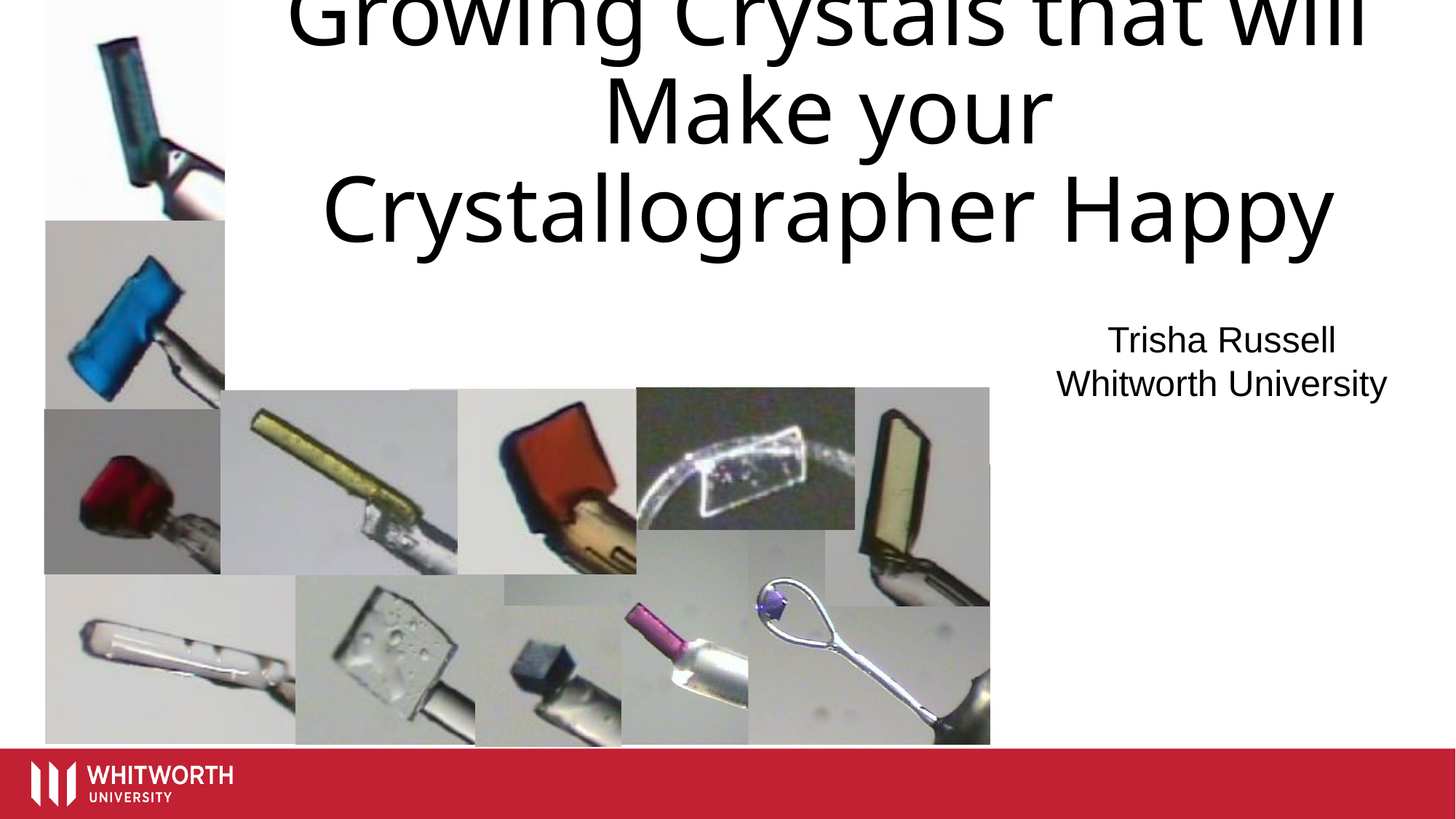

# Growing Crystals that will Make your Crystallographer Happy
Trisha Russell
Whitworth University

## Slide 3
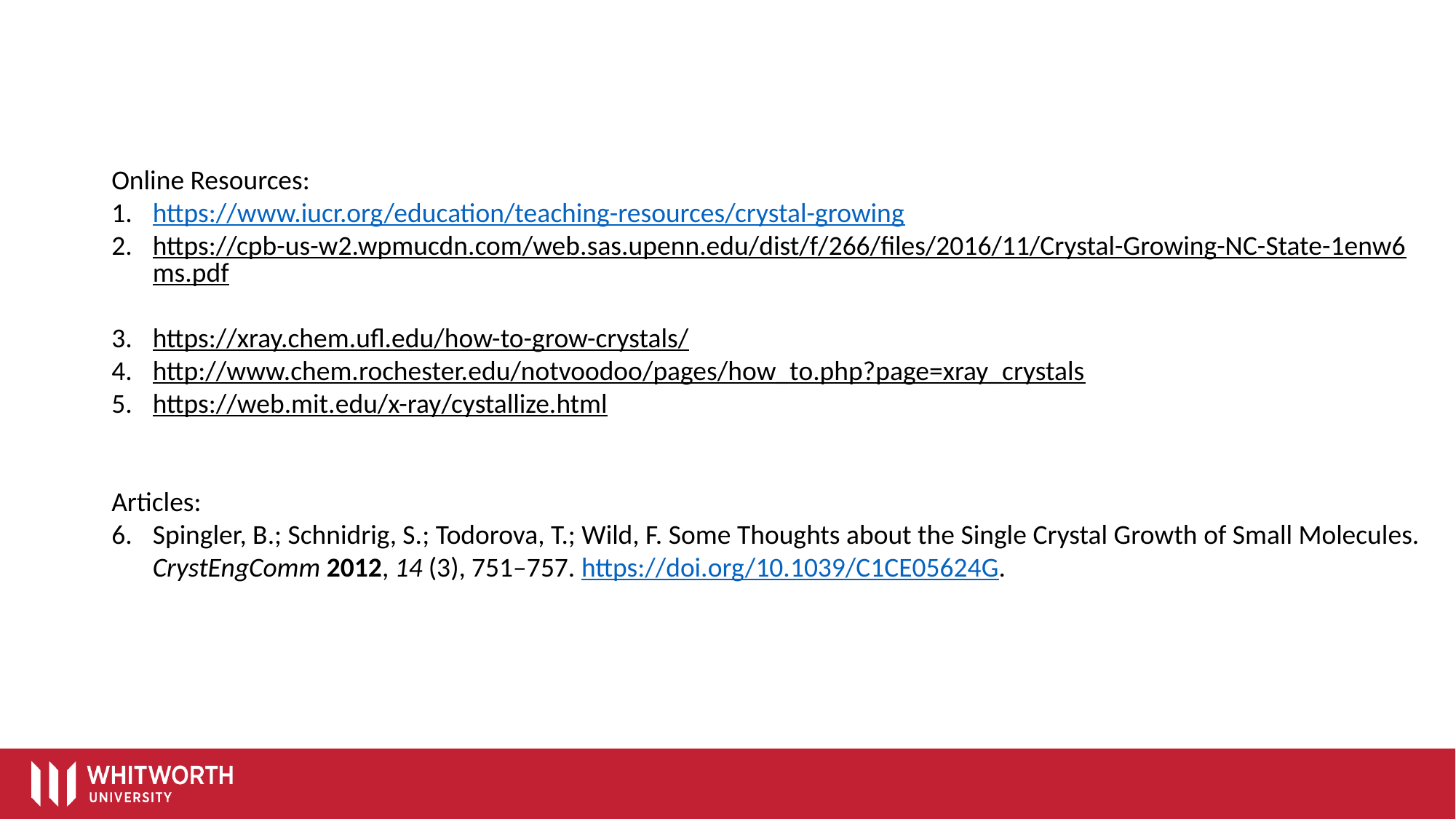

Online Resources:
https://www.iucr.org/education/teaching-resources/crystal-growing
https://cpb-us-w2.wpmucdn.com/web.sas.upenn.edu/dist/f/266/files/2016/11/Crystal-Growing-NC-State-1enw6ms.pdf
https://xray.chem.ufl.edu/how-to-grow-crystals/
http://www.chem.rochester.edu/notvoodoo/pages/how_to.php?page=xray_crystals
https://web.mit.edu/x-ray/cystallize.html
Articles:
Spingler, B.; Schnidrig, S.; Todorova, T.; Wild, F. Some Thoughts about the Single Crystal Growth of Small Molecules. CrystEngComm 2012, 14 (3), 751–757. https://doi.org/10.1039/C1CE05624G.

## Slide 4
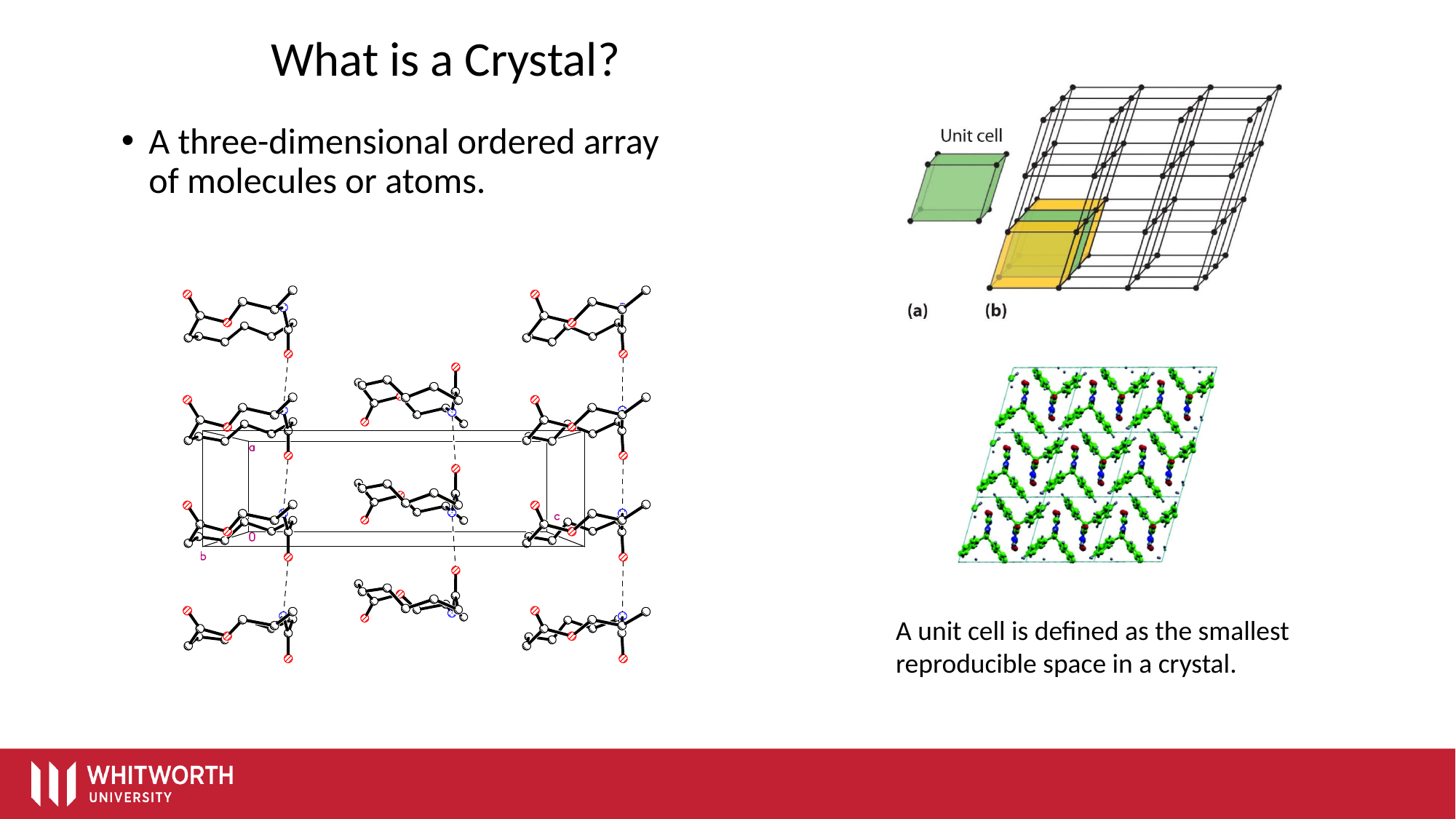

# What is a Crystal?
A three-dimensional ordered array of molecules or atoms.
A unit cell is defined as the smallest reproducible space in a crystal.

## Slide 5
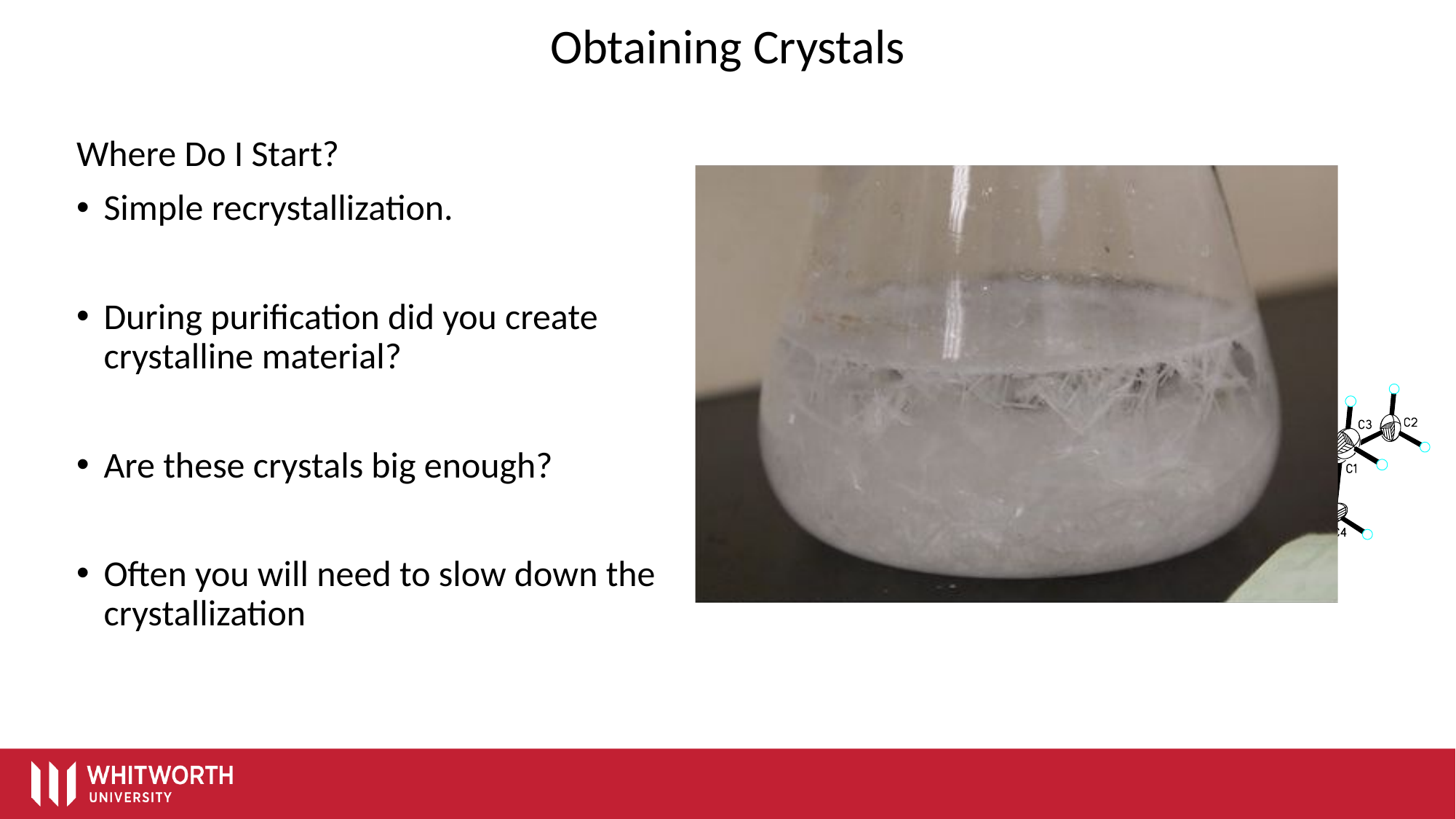

# Obtaining Crystals
Where Do I Start?
Simple recrystallization.
During purification did you create crystalline material?
Are these crystals big enough?
Often you will need to slow down the crystallization

## Slide 6
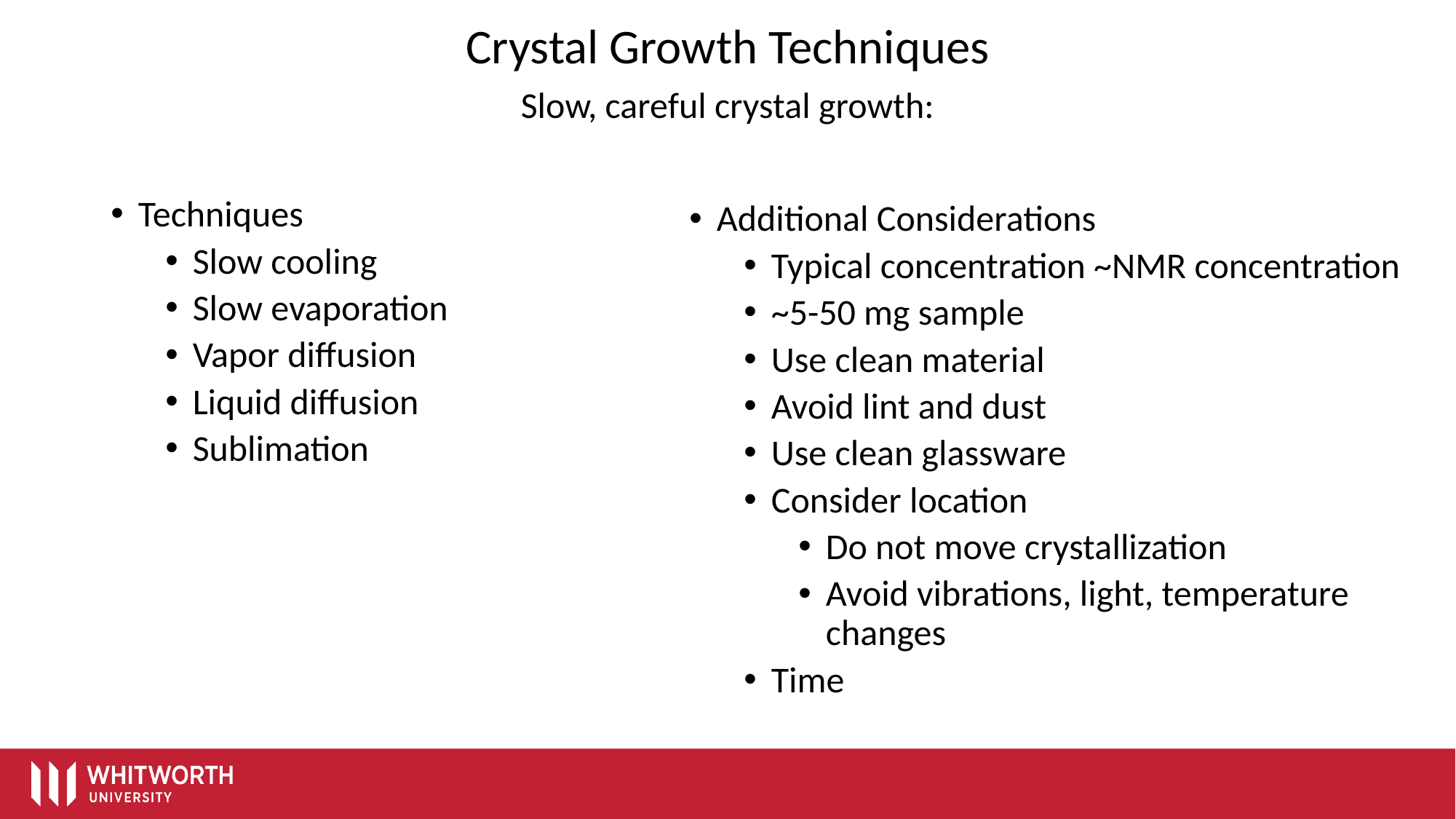

# Crystal Growth Techniques
Slow, careful crystal growth:
Techniques
Slow cooling
Slow evaporation
Vapor diffusion
Liquid diffusion
Sublimation
Additional Considerations
Typical concentration ~NMR concentration
~5-50 mg sample
Use clean material
Avoid lint and dust
Use clean glassware
Consider location
Do not move crystallization
Avoid vibrations, light, temperature changes
Time

## Slide 7
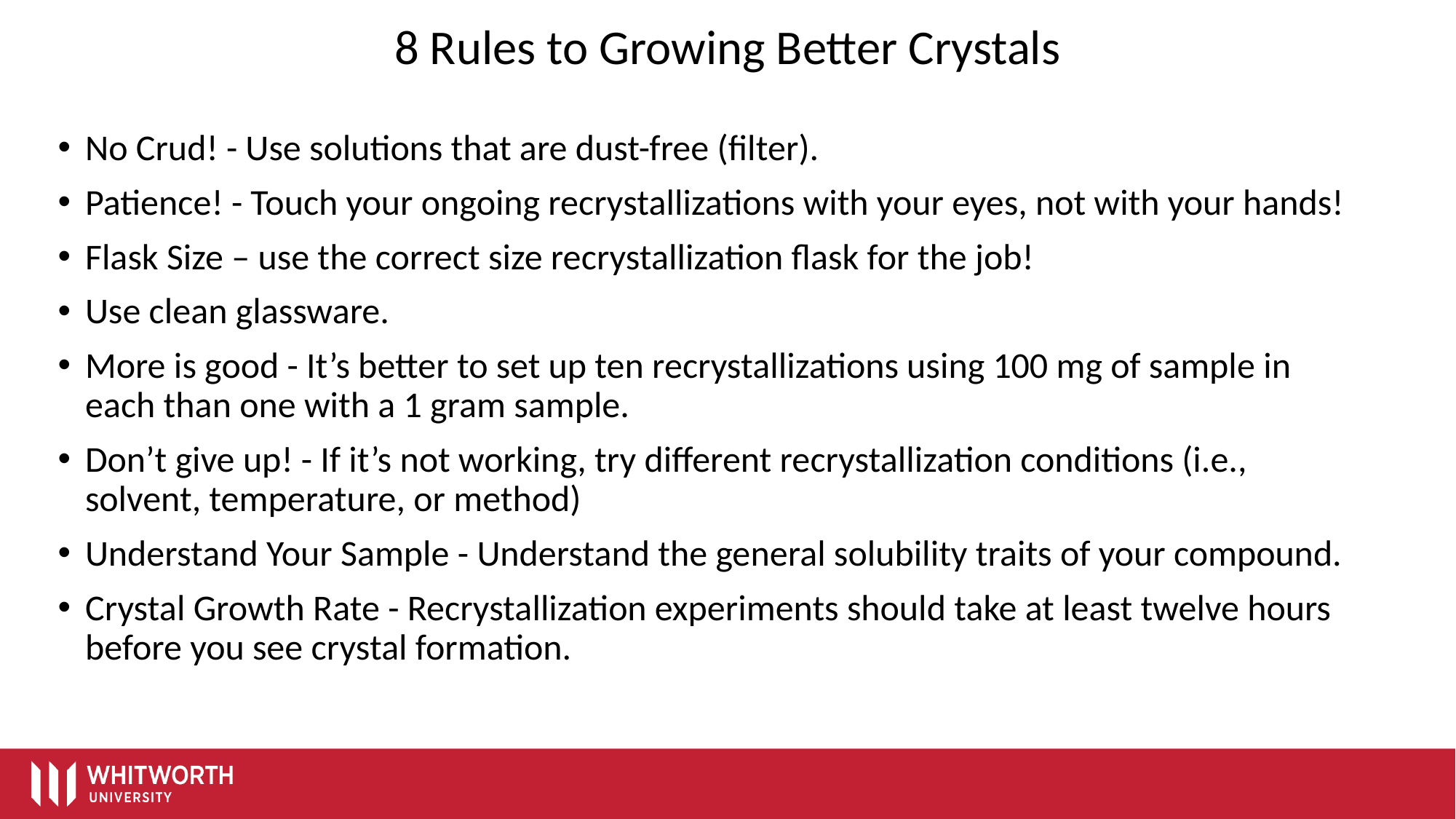

# 8 Rules to Growing Better Crystals
No Crud! - Use solutions that are dust-free (filter).
Patience! - Touch your ongoing recrystallizations with your eyes, not with your hands!
Flask Size – use the correct size recrystallization flask for the job!
Use clean glassware.
More is good - It’s better to set up ten recrystallizations using 100 mg of sample in each than one with a 1 gram sample.
Don’t give up! - If it’s not working, try different recrystallization conditions (i.e., solvent, temperature, or method)
Understand Your Sample - Understand the general solubility traits of your compound.
Crystal Growth Rate - Recrystallization experiments should take at least twelve hours before you see crystal formation.

## Slide 8
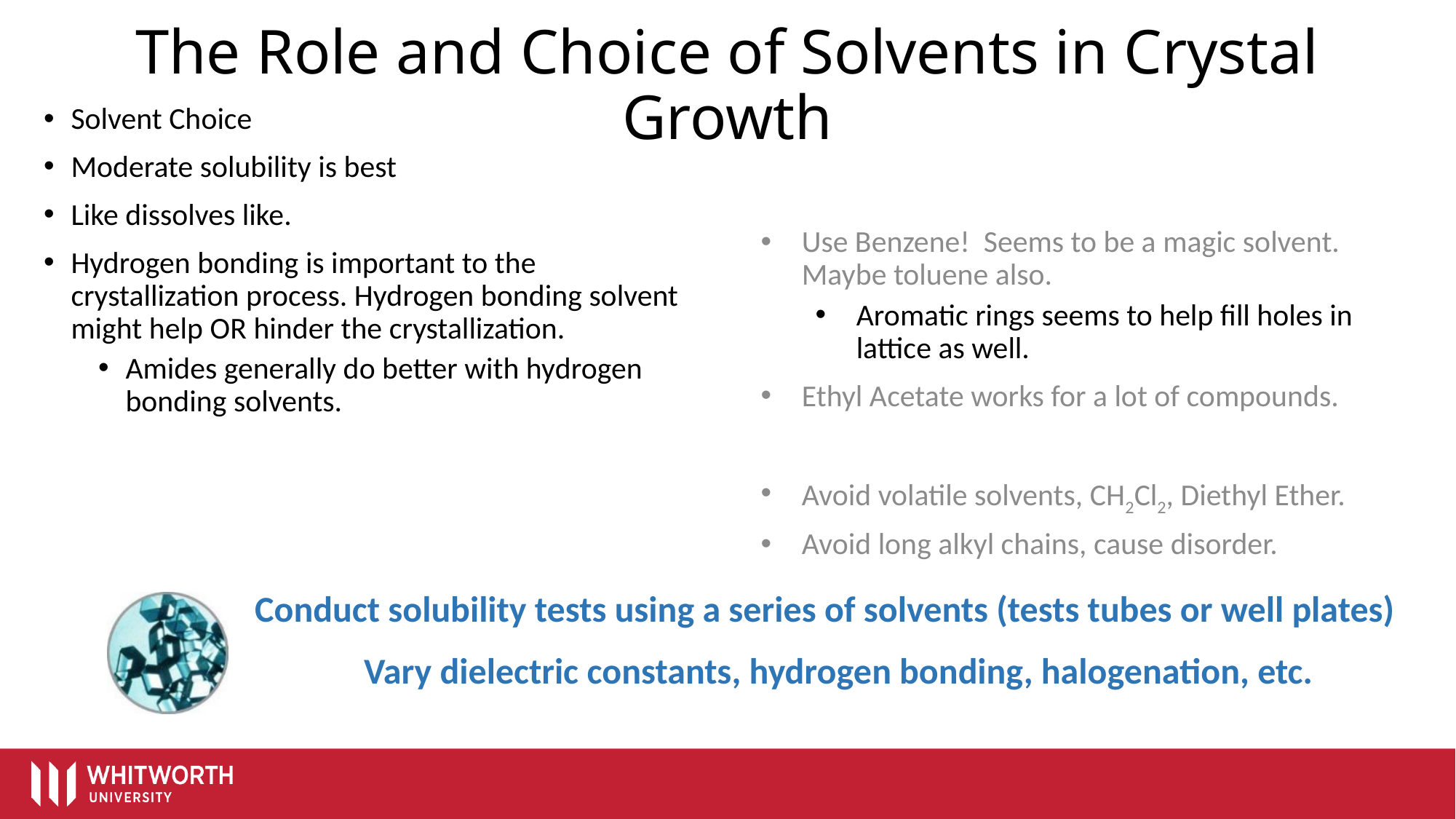

# The Role and Choice of Solvents in Crystal Growth
Solvent Choice
Moderate solubility is best
Like dissolves like.
Hydrogen bonding is important to the crystallization process. Hydrogen bonding solvent might help OR hinder the crystallization.
Amides generally do better with hydrogen bonding solvents.
Use Benzene! Seems to be a magic solvent. Maybe toluene also.
Aromatic rings seems to help fill holes in lattice as well.
Ethyl Acetate works for a lot of compounds.
Avoid volatile solvents, CH2Cl2, Diethyl Ether.
Avoid long alkyl chains, cause disorder.
Conduct solubility tests using a series of solvents (tests tubes or well plates)
	Vary dielectric constants, hydrogen bonding, halogenation, etc.

## Slide 9
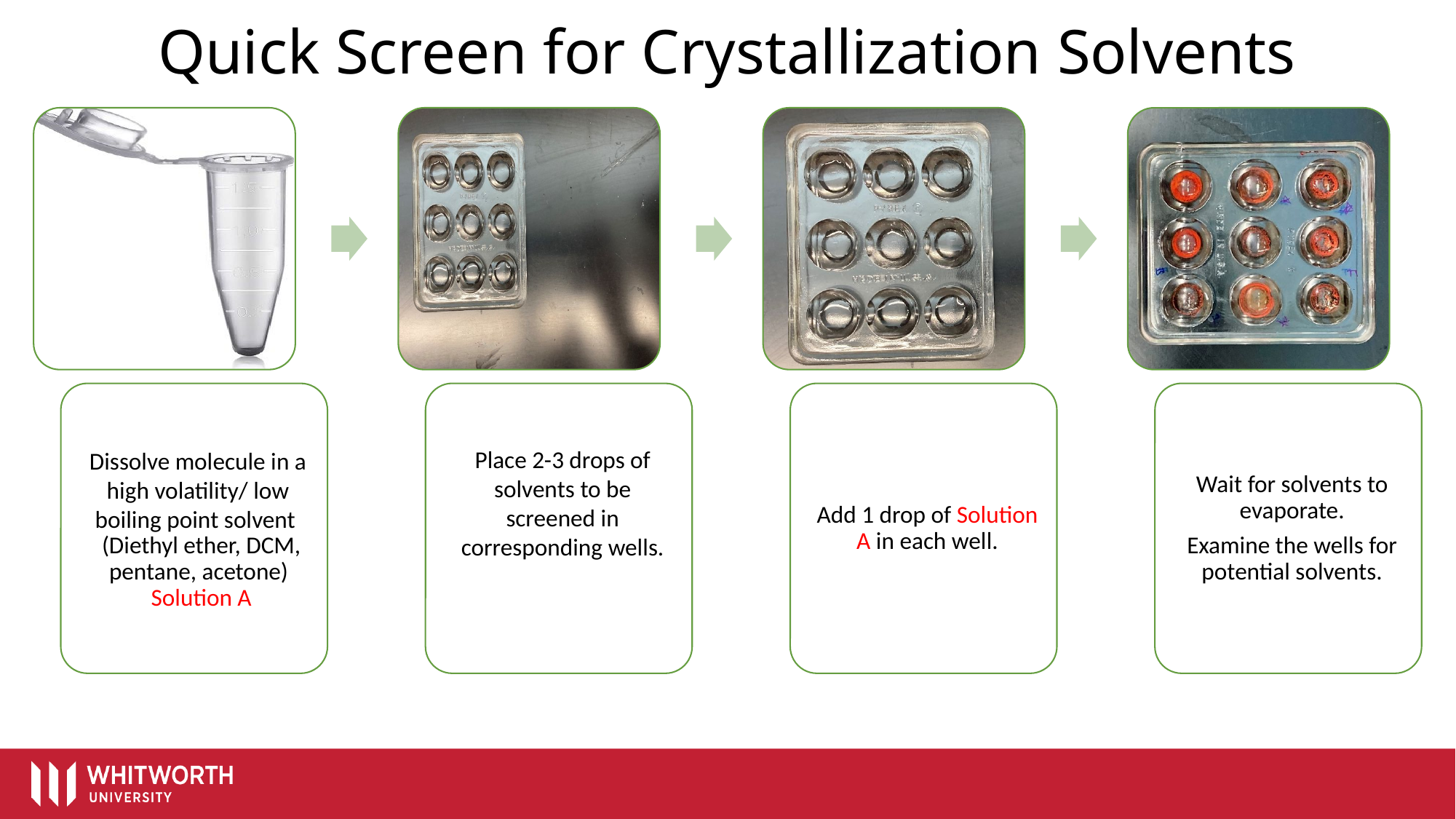

# Quick Screen for Crystallization Solvents

## Slide 10
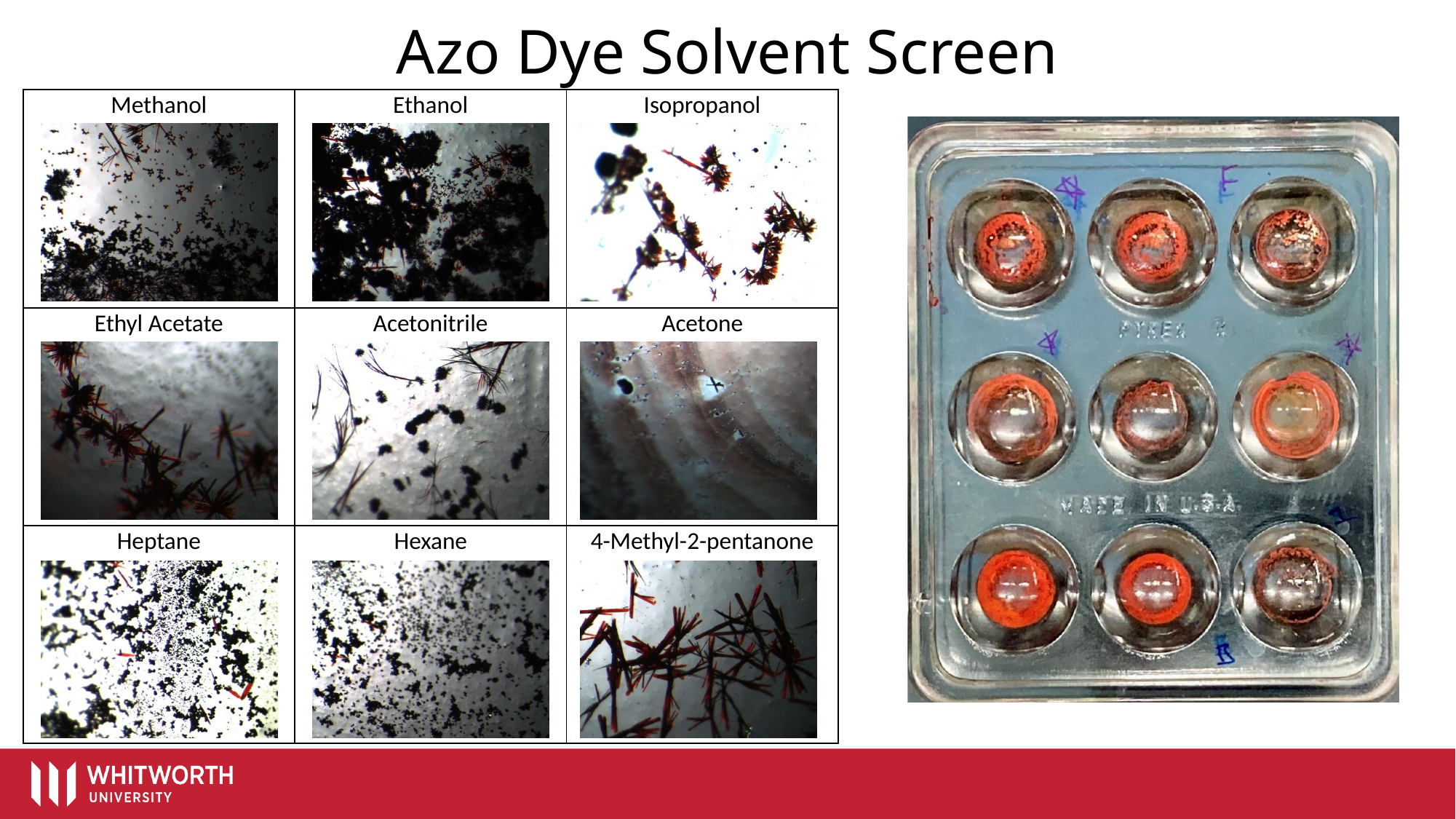

# Azo Dye Solvent Screen
| Methanol | Ethanol | Isopropanol |
| --- | --- | --- |
| Ethyl Acetate | Acetonitrile | Acetone |
| Heptane | Hexane | 4-Methyl-2-pentanone |

## Slide 11
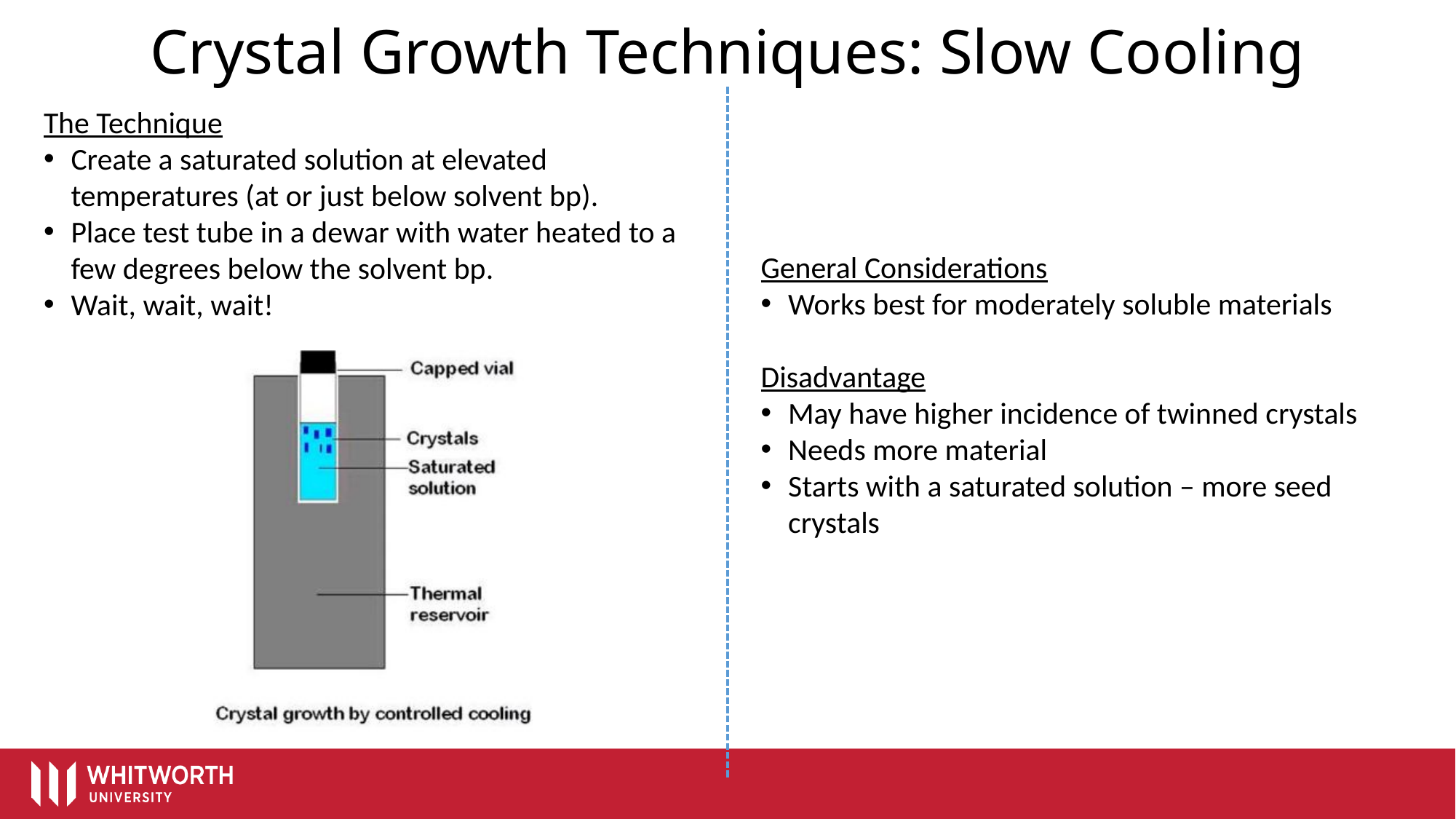

# Crystal Growth Techniques: Slow Cooling
The Technique
Create a saturated solution at elevated temperatures (at or just below solvent bp).
Place test tube in a dewar with water heated to a few degrees below the solvent bp.
Wait, wait, wait!
General Considerations
Works best for moderately soluble materials
Disadvantage
May have higher incidence of twinned crystals
Needs more material
Starts with a saturated solution – more seed crystals

## Slide 12
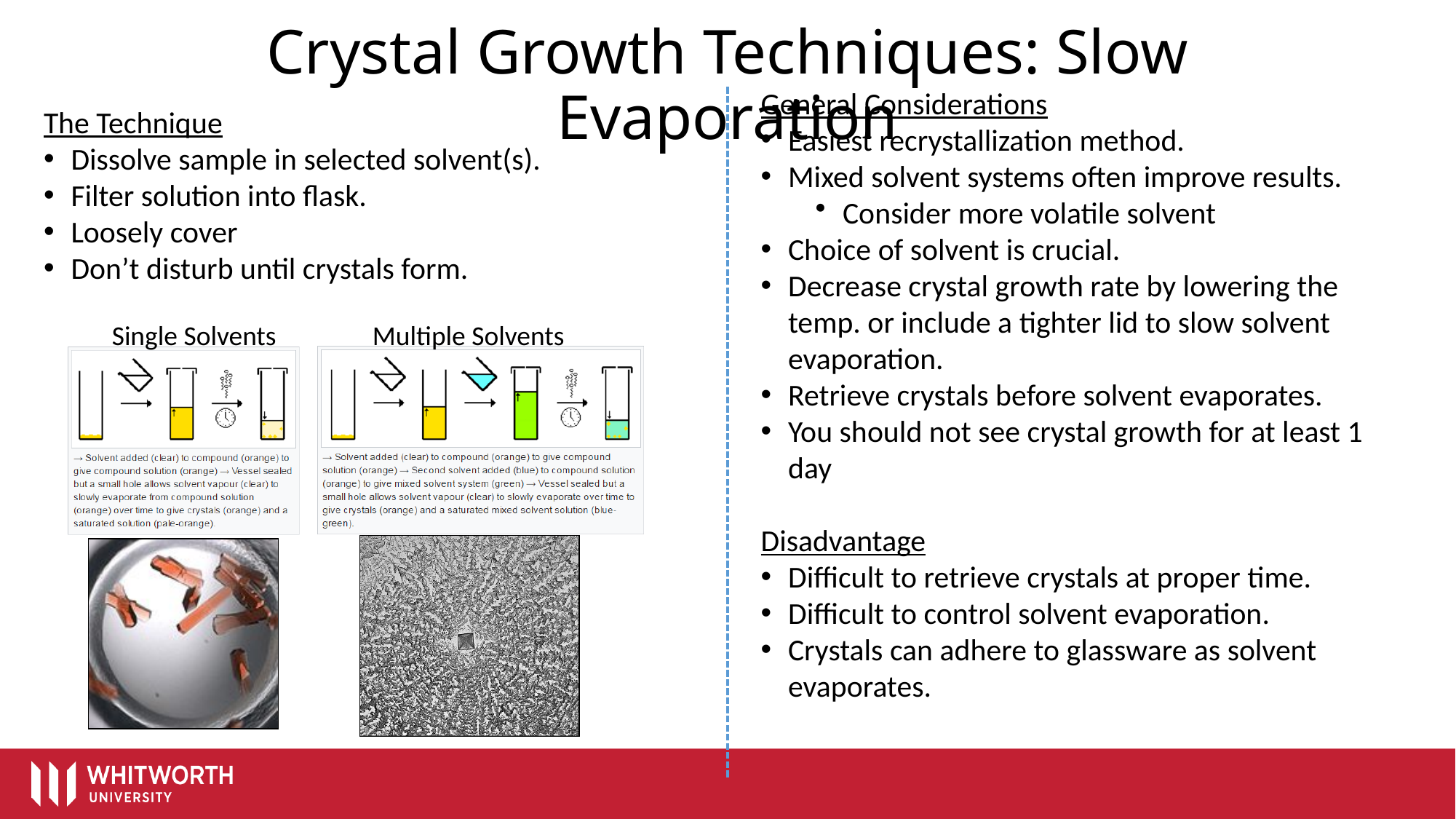

# Crystal Growth Techniques: Slow Evaporation
The Technique
Dissolve sample in selected solvent(s).
Filter solution into flask.
Loosely cover
Don’t disturb until crystals form.
General Considerations
Easiest recrystallization method.
Mixed solvent systems often improve results.
Consider more volatile solvent
Choice of solvent is crucial.
Decrease crystal growth rate by lowering the temp. or include a tighter lid to slow solvent evaporation.
Retrieve crystals before solvent evaporates.
You should not see crystal growth for at least 1 day
Disadvantage
Difficult to retrieve crystals at proper time.
Difficult to control solvent evaporation.
Crystals can adhere to glassware as solvent evaporates.
Single Solvents
Multiple Solvents

## Slide 13
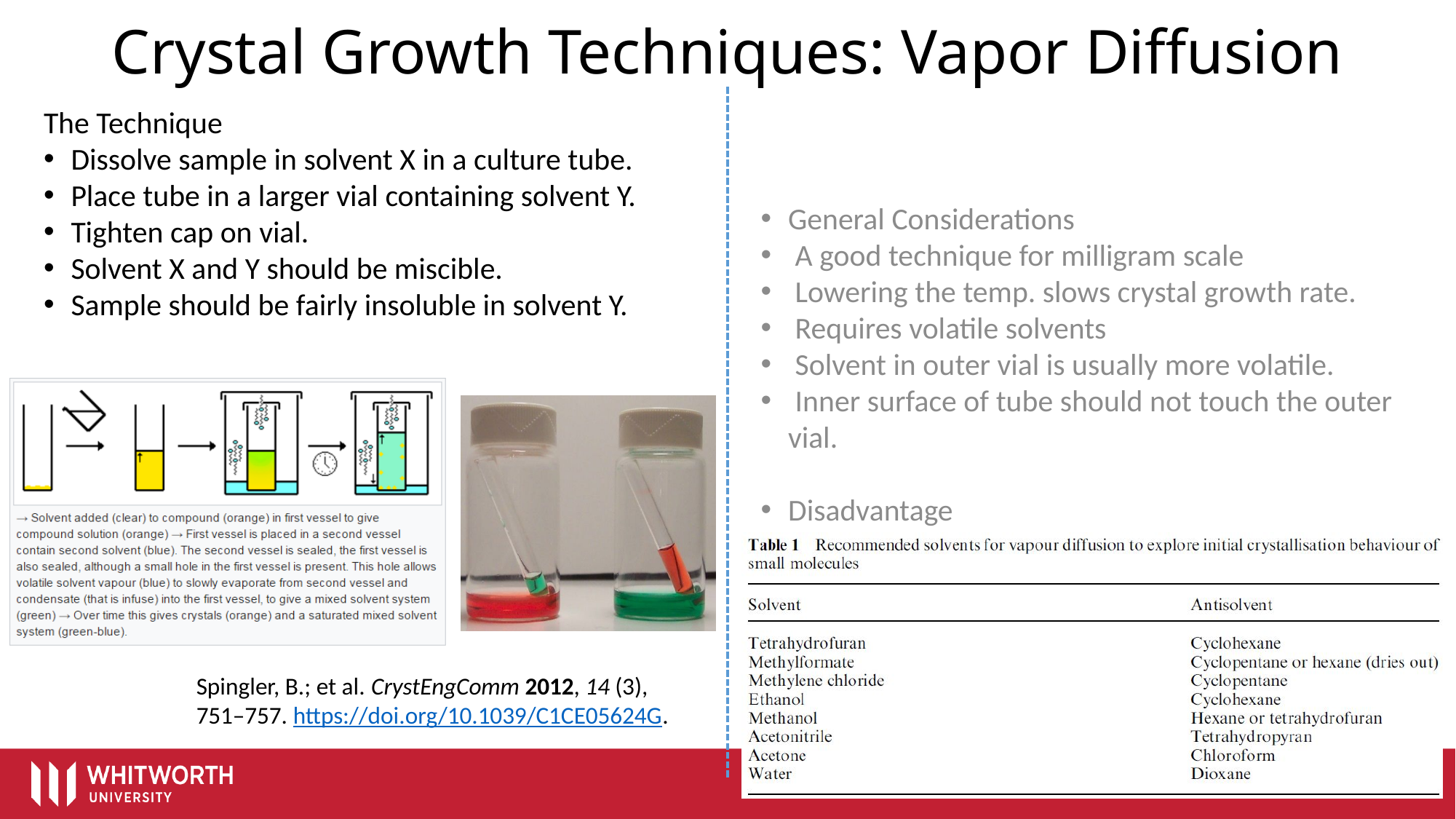

# Crystal Growth Techniques: Vapor Diffusion
The Technique
Dissolve sample in solvent X in a culture tube.
Place tube in a larger vial containing solvent Y.
Tighten cap on vial.
Solvent X and Y should be miscible.
Sample should be fairly insoluble in solvent Y.
General Considerations
 A good technique for milligram scale
 Lowering the temp. slows crystal growth rate.
 Requires volatile solvents
 Solvent in outer vial is usually more volatile.
 Inner surface of tube should not touch the outer vial.
Disadvantage
More complicated set up
Identifying solvents is challenging
Spingler, B.; et al. CrystEngComm 2012, 14 (3), 751–757. https://doi.org/10.1039/C1CE05624G.

## Slide 14
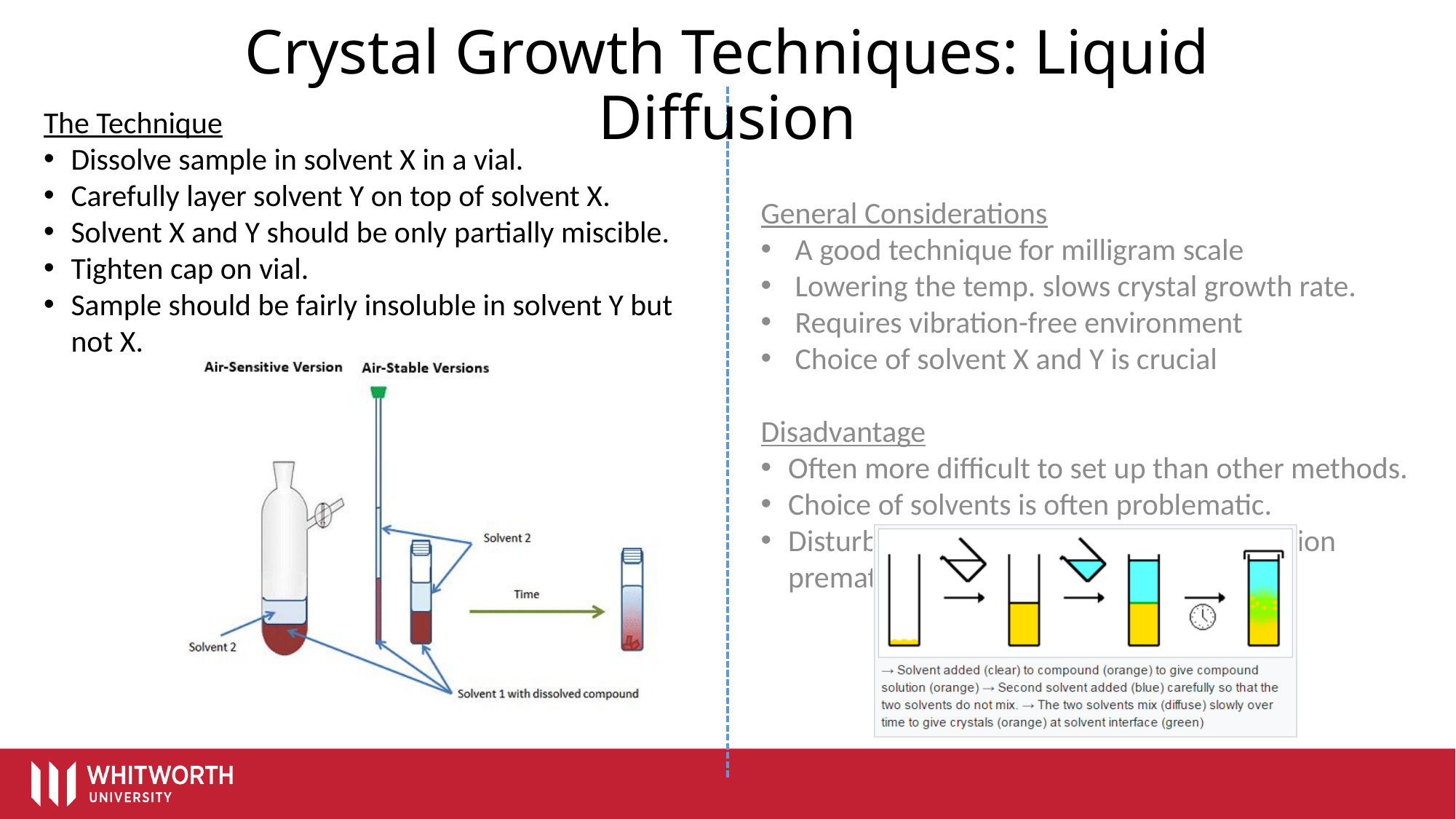

# Crystal Growth Techniques: Liquid Diffusion
The Technique
Dissolve sample in solvent X in a vial.
Carefully layer solvent Y on top of solvent X.
Solvent X and Y should be only partially miscible.
Tighten cap on vial.
Sample should be fairly insoluble in solvent Y but not X.
General Considerations
 A good technique for milligram scale
 Lowering the temp. slows crystal growth rate.
 Requires vibration-free environment
 Choice of solvent X and Y is crucial
Disadvantage
Often more difficult to set up than other methods.
Choice of solvents is often problematic.
Disturbing the set-up can start crystallization prematurely.

## Slide 15
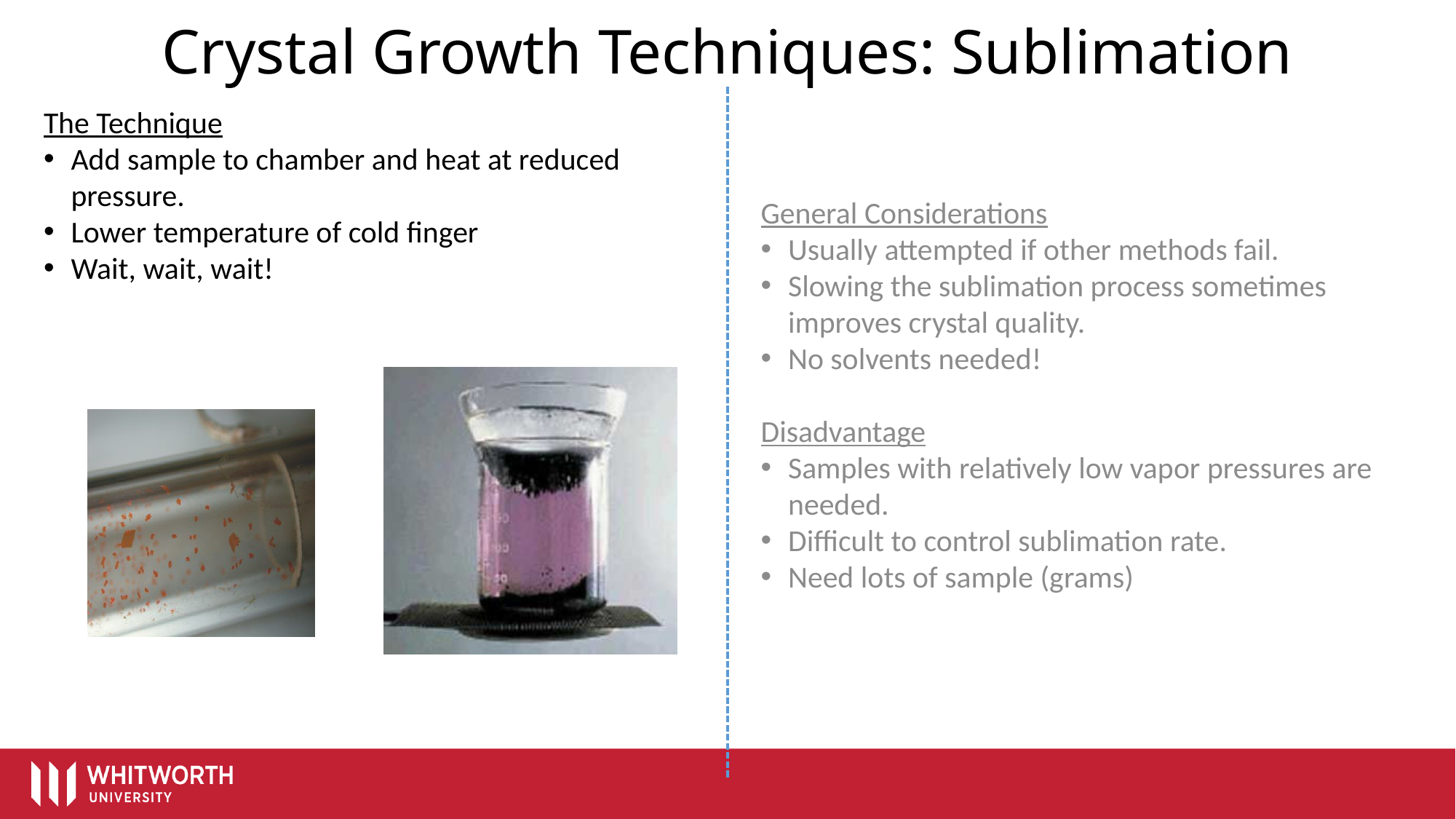

# Crystal Growth Techniques: Sublimation
The Technique
Add sample to chamber and heat at reduced pressure.
Lower temperature of cold finger
Wait, wait, wait!
General Considerations
Usually attempted if other methods fail.
Slowing the sublimation process sometimes improves crystal quality.
No solvents needed!
Disadvantage
Samples with relatively low vapor pressures are needed.
Difficult to control sublimation rate.
Need lots of sample (grams)

## Slide 16
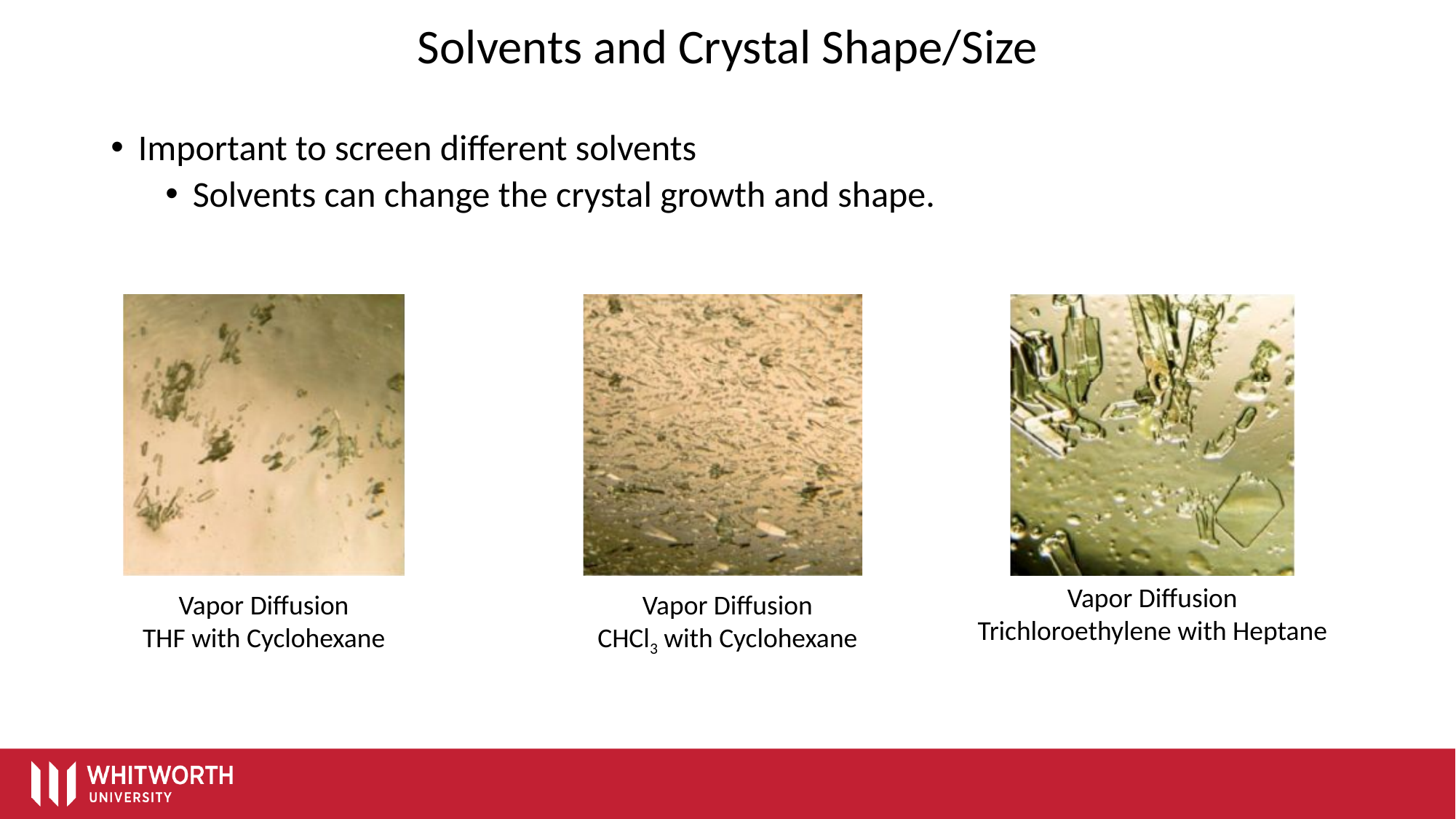

# Solvents and Crystal Shape/Size
Important to screen different solvents
Solvents can change the crystal growth and shape.
Vapor Diffusion
Trichloroethylene with Heptane
Vapor Diffusion
CHCl3 with Cyclohexane
Vapor Diffusion
THF with Cyclohexane

## Slide 17
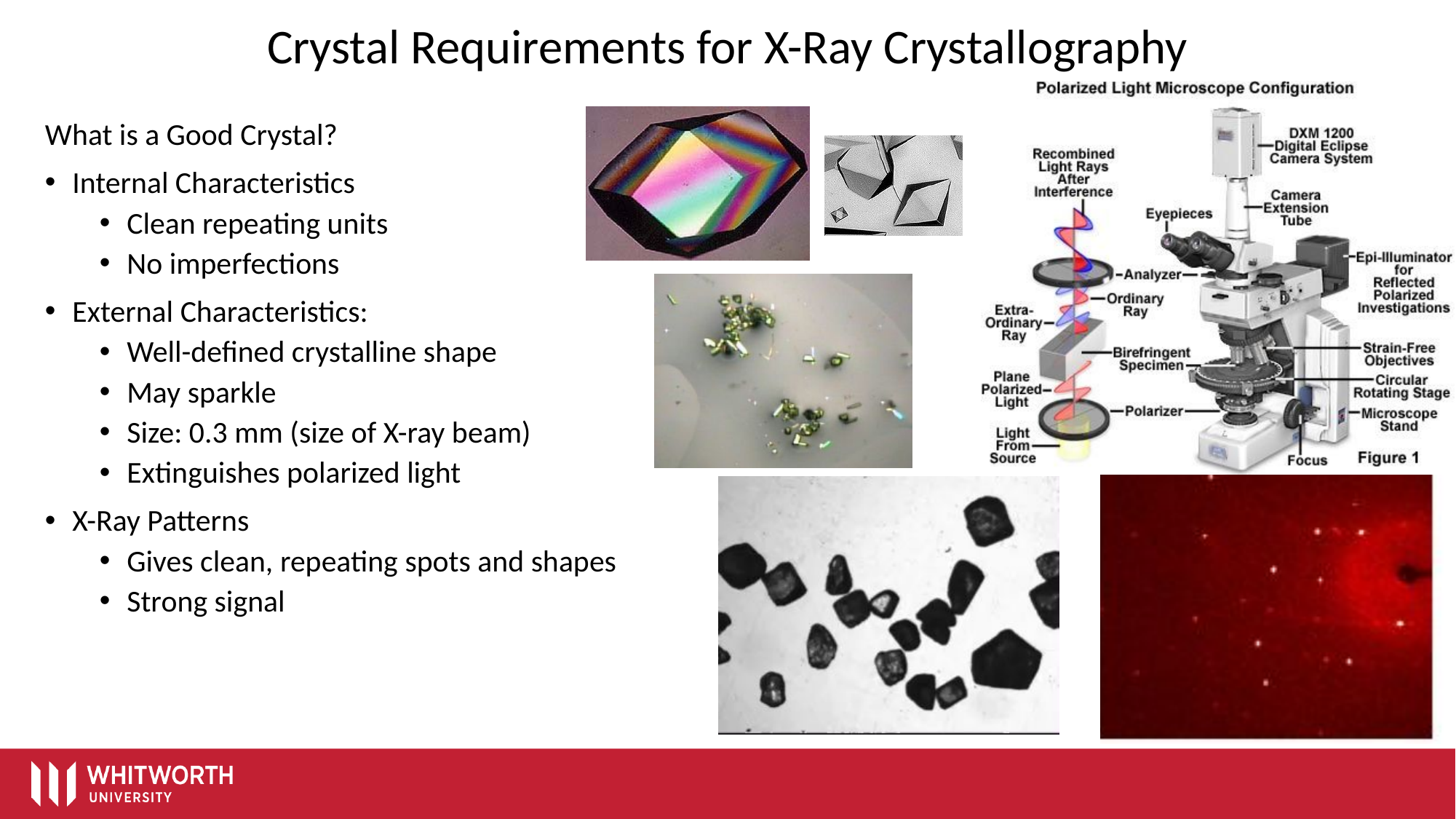

# Crystal Requirements for X-Ray Crystallography
What is a Good Crystal?
Internal Characteristics
Clean repeating units
No imperfections
External Characteristics:
Well-defined crystalline shape
May sparkle
Size: 0.3 mm (size of X-ray beam)
Extinguishes polarized light
X-Ray Patterns
Gives clean, repeating spots and shapes
Strong signal

## Slide 18
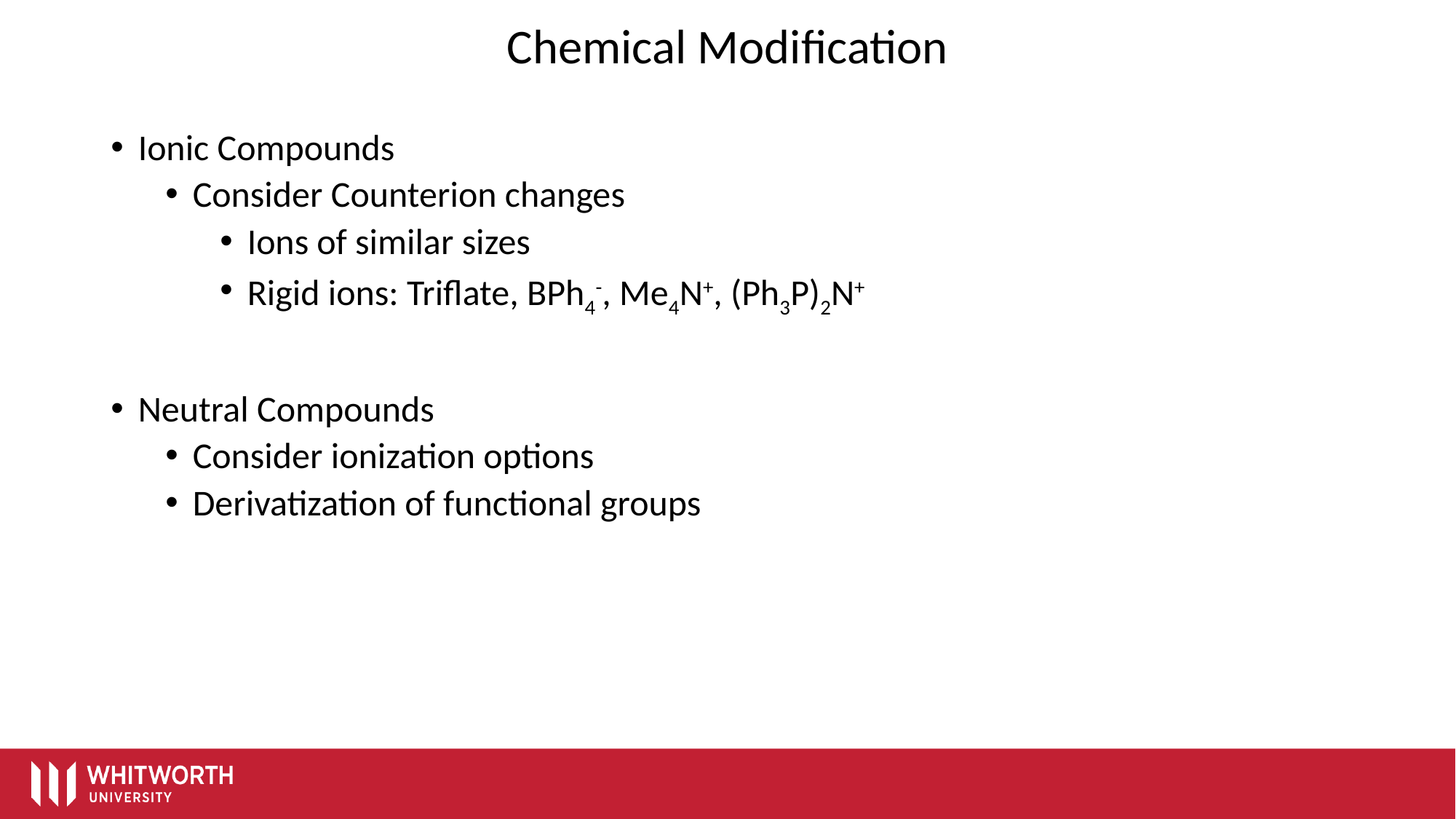

# Chemical Modification
Ionic Compounds
Consider Counterion changes
Ions of similar sizes
Rigid ions: Triflate, BPh4-, Me4N+, (Ph3P)2N+
Neutral Compounds
Consider ionization options
Derivatization of functional groups

## Slide 19
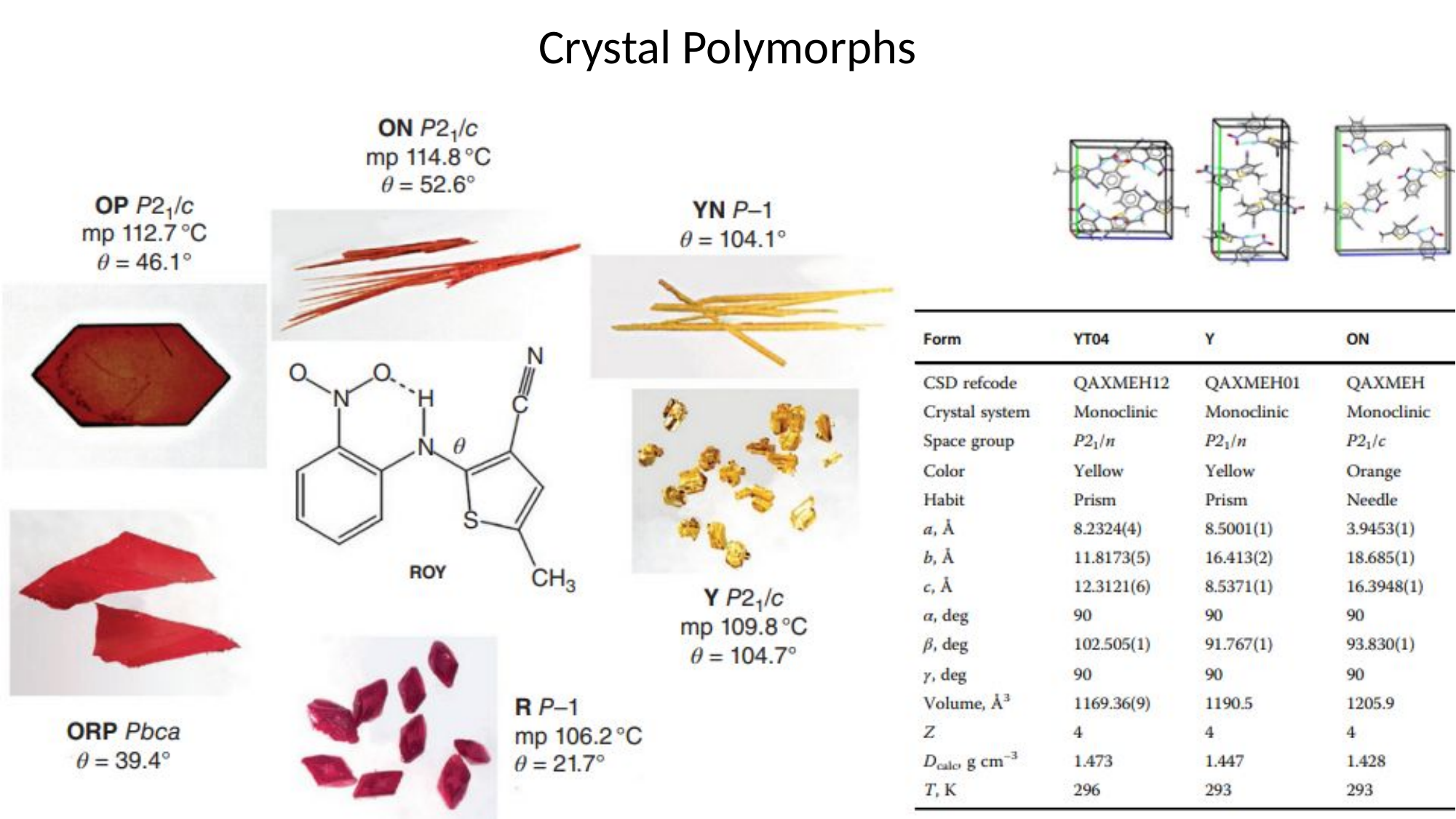

# Crystal Polymorphs

## Slide 20
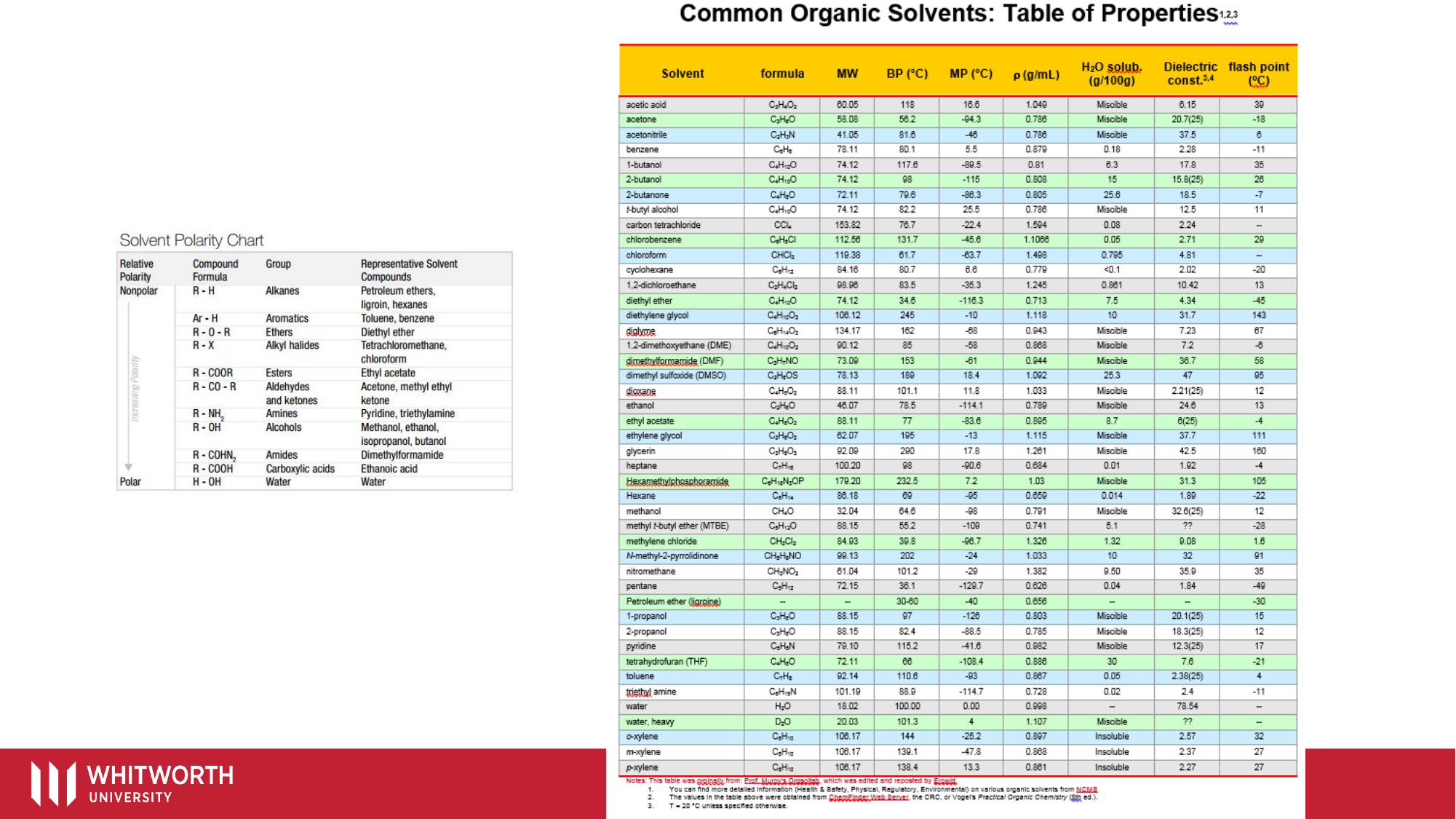

## Slide 21
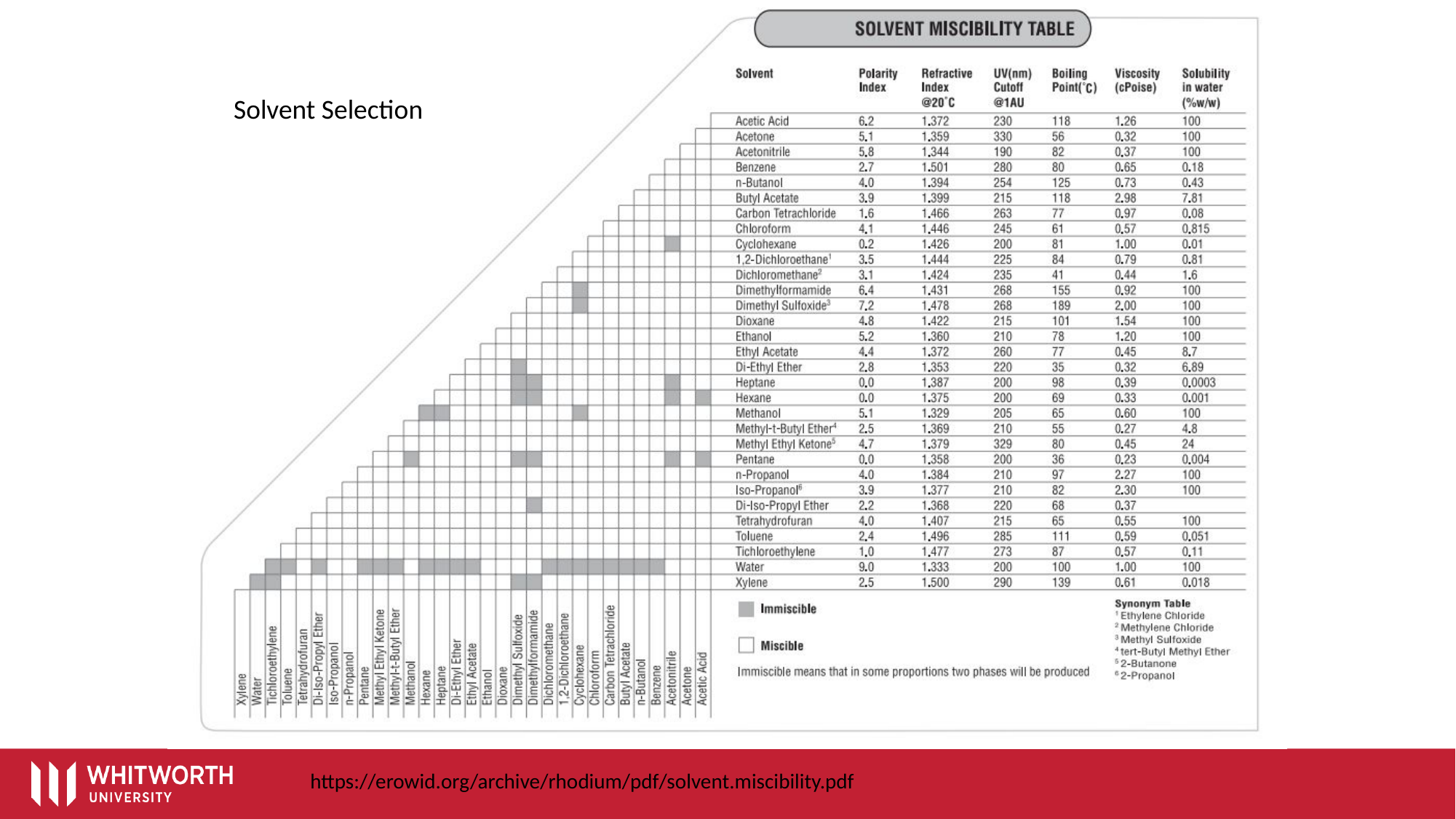

Solvent Selection
https://erowid.org/archive/rhodium/pdf/solvent.miscibility.pdf
