# Supplementary material for: Crystallographic workshops – a primer and perspective from Whitworth University’s Summer Crystallography Institute
Source: Acta Crystallogr E Crystallogr Commun. 2026 Feb 3;82(Pt 3):313–9. doi: 10.1107/S2056989026000939 (PMC12961664; doi:10.1107/S2056989026000939)
Supplement: Supplementary file 1 [file e-82-00313-sup2.zip › Lectures/SCI_Crystallog_Symmetry.pptx]

## Slide 1
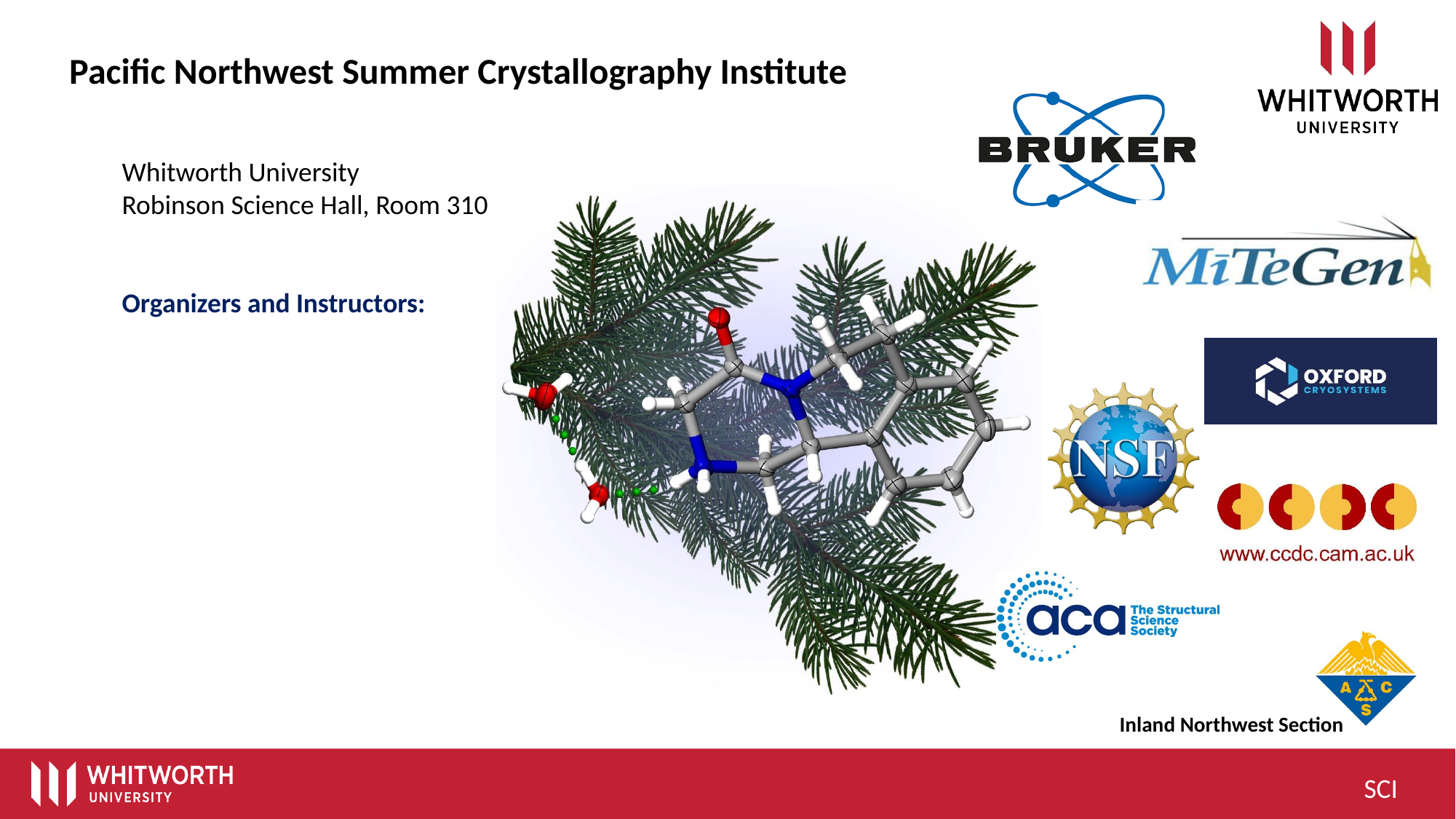

Pacific Northwest Summer Crystallography Institute
Whitworth University
Robinson Science Hall, Room 310
Organizers and Instructors:
Inland Northwest Section
SCI

## Slide 2
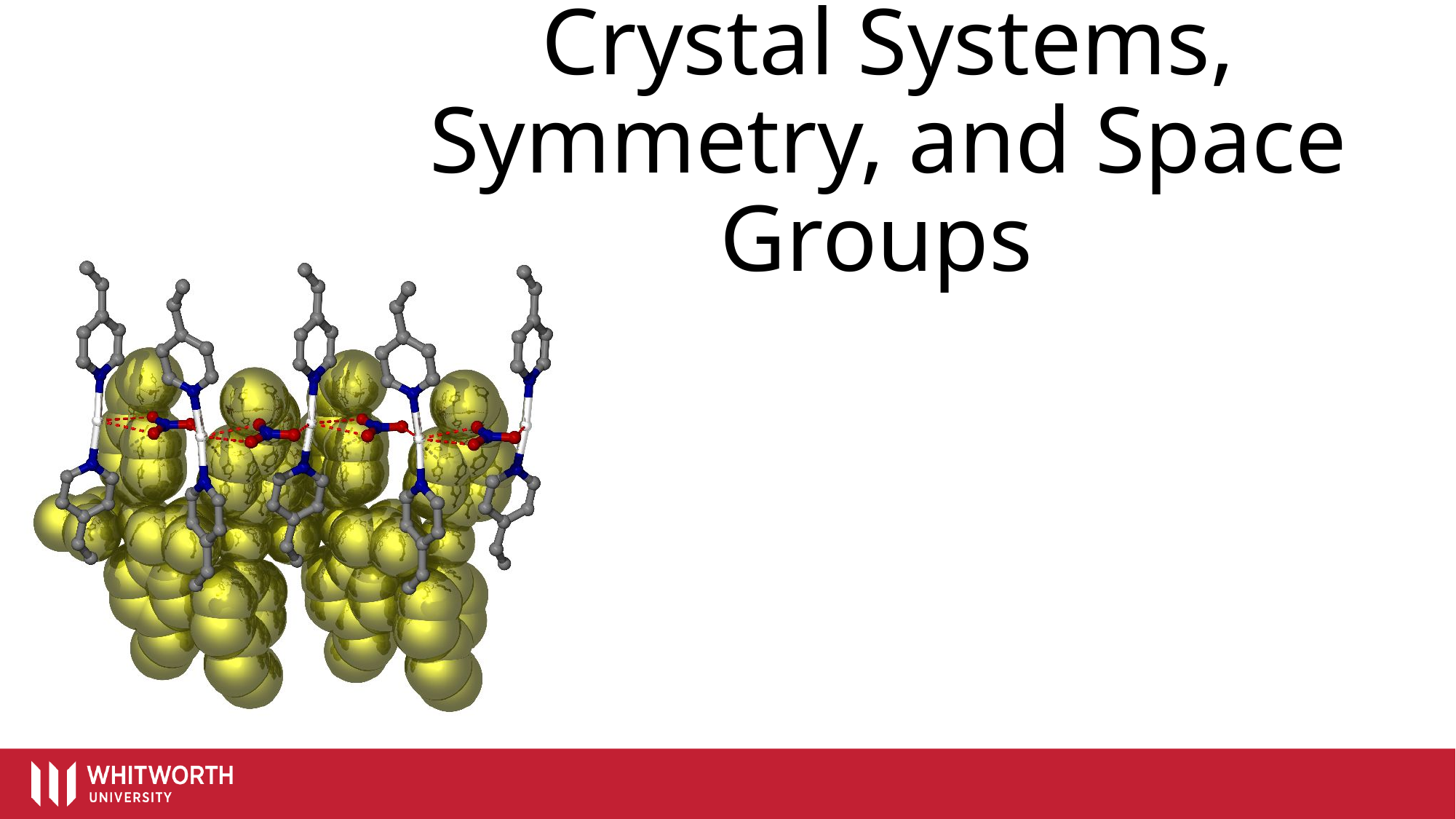

# Crystal Systems, Symmetry, and Space Groups

## Slide 3
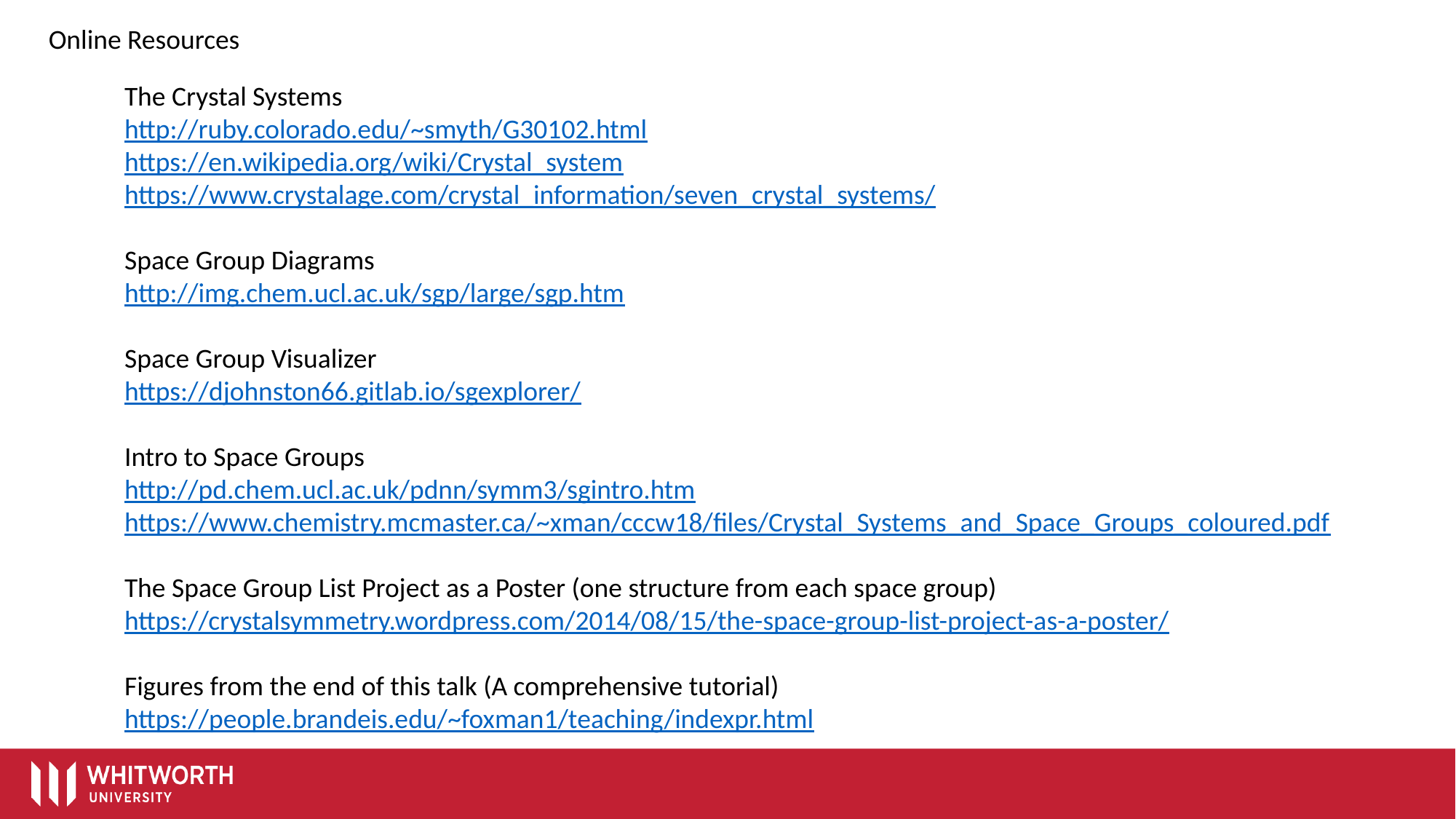

Online Resources
The Crystal Systems
http://ruby.colorado.edu/~smyth/G30102.html
https://en.wikipedia.org/wiki/Crystal_system
https://www.crystalage.com/crystal_information/seven_crystal_systems/
Space Group Diagrams
http://img.chem.ucl.ac.uk/sgp/large/sgp.htm
Space Group Visualizer
https://djohnston66.gitlab.io/sgexplorer/
Intro to Space Groups
http://pd.chem.ucl.ac.uk/pdnn/symm3/sgintro.htm
https://www.chemistry.mcmaster.ca/~xman/cccw18/files/Crystal_Systems_and_Space_Groups_coloured.pdf
The Space Group List Project as a Poster (one structure from each space group)
https://crystalsymmetry.wordpress.com/2014/08/15/the-space-group-list-project-as-a-poster/
Figures from the end of this talk (A comprehensive tutorial)
https://people.brandeis.edu/~foxman1/teaching/indexpr.html

## Slide 4
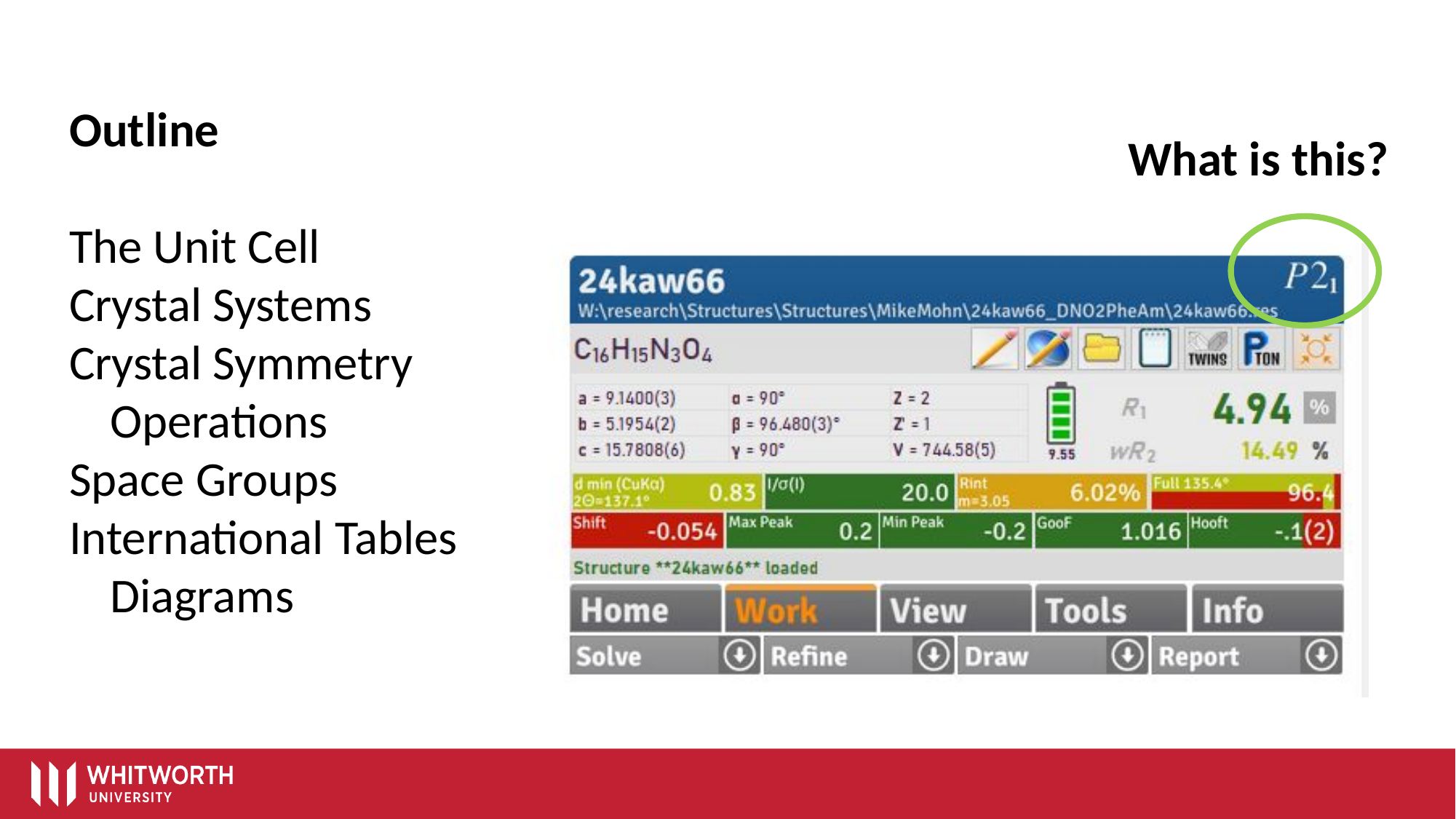

Outline
The Unit Cell
Crystal Systems
Crystal Symmetry Operations
Space Groups
International Tables Diagrams
What is this?

## Slide 5
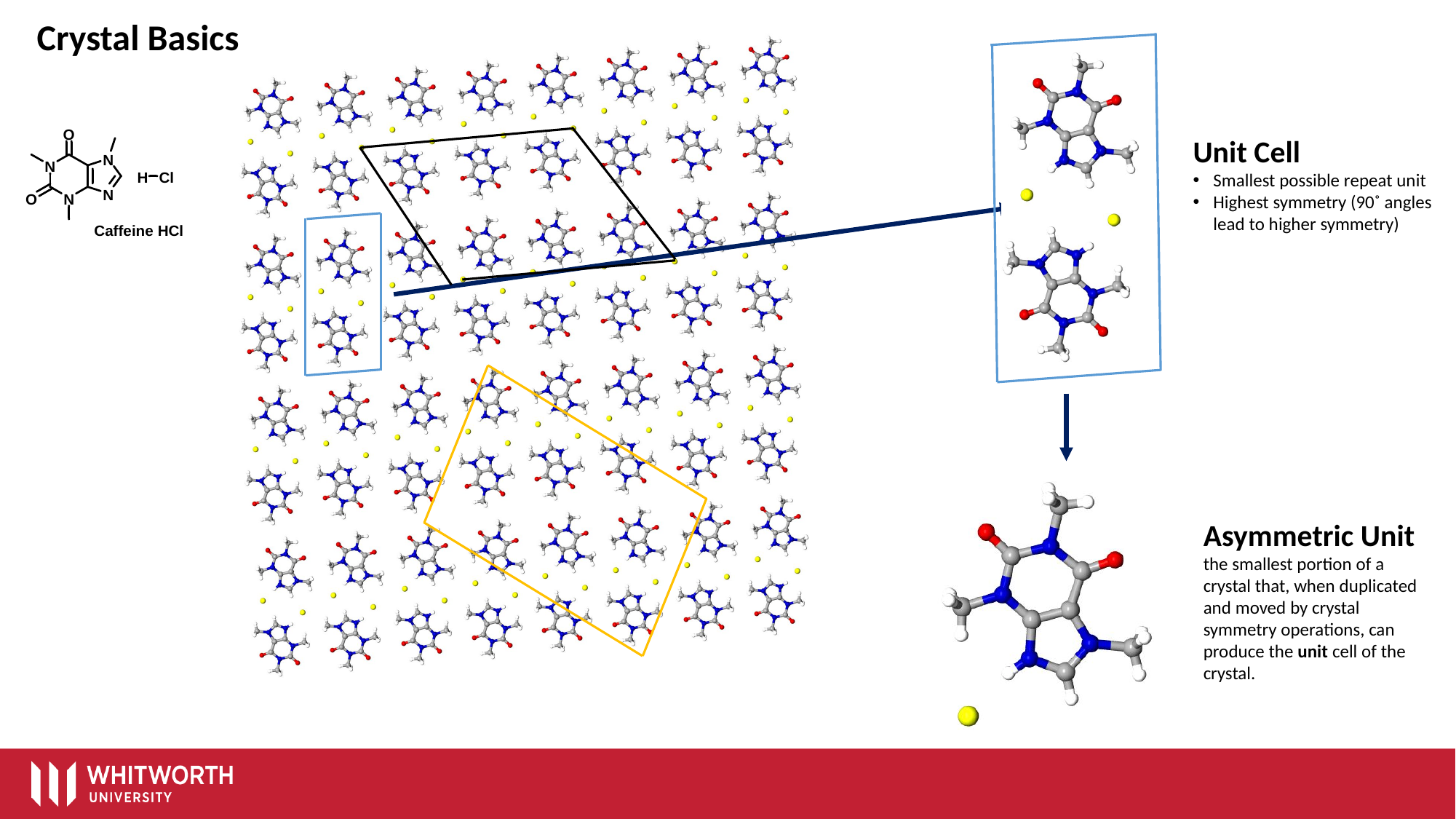

Crystal Basics
Unit Cell
Smallest possible repeat unit
Highest symmetry (90˚ angles lead to higher symmetry)
Asymmetric Unit
the smallest portion of a crystal that, when duplicated and moved by crystal symmetry operations, can produce the unit cell of the crystal.

## Slide 6
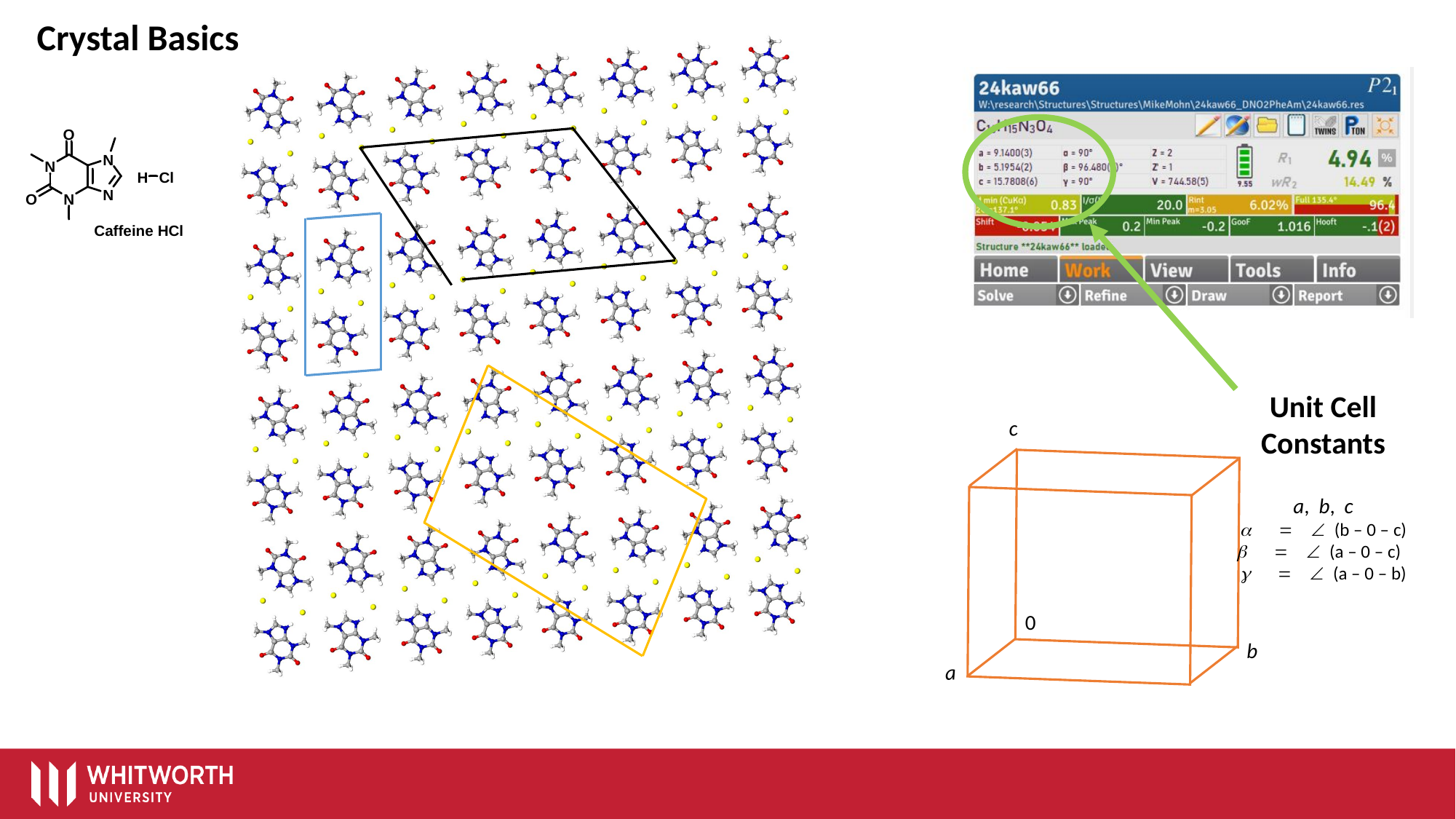

Crystal Basics
Unit Cell Constants
a, b, c
a =  (b – 0 – c)
b =  (a – 0 – c)
g =  (a – 0 – b)
c
0
a
b

## Slide 7
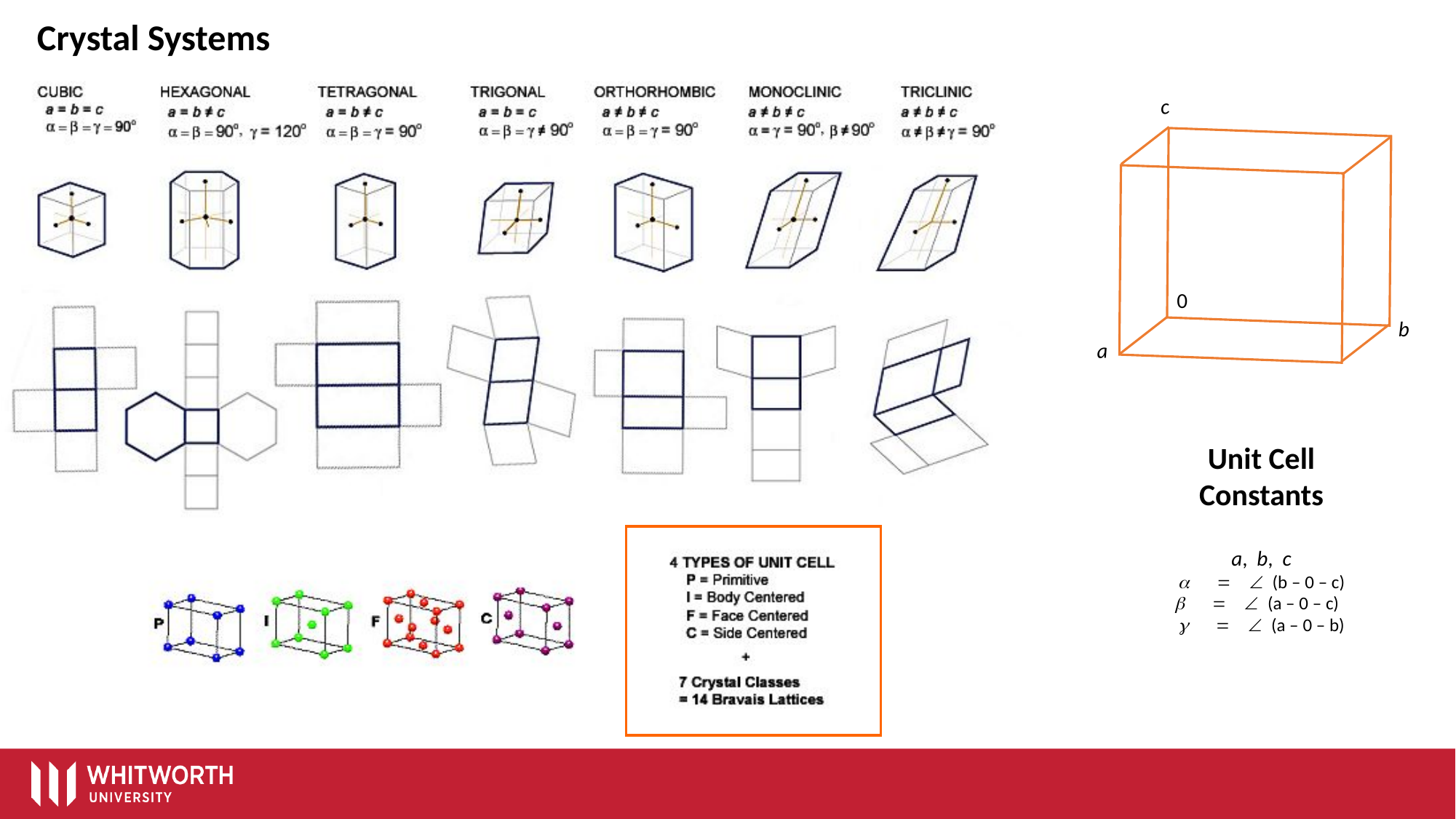

Crystal Systems
c
0
a
b
Unit Cell Constants
a, b, c
a =  (b – 0 – c)
b =  (a – 0 – c)
g =  (a – 0 – b)

## Slide 8
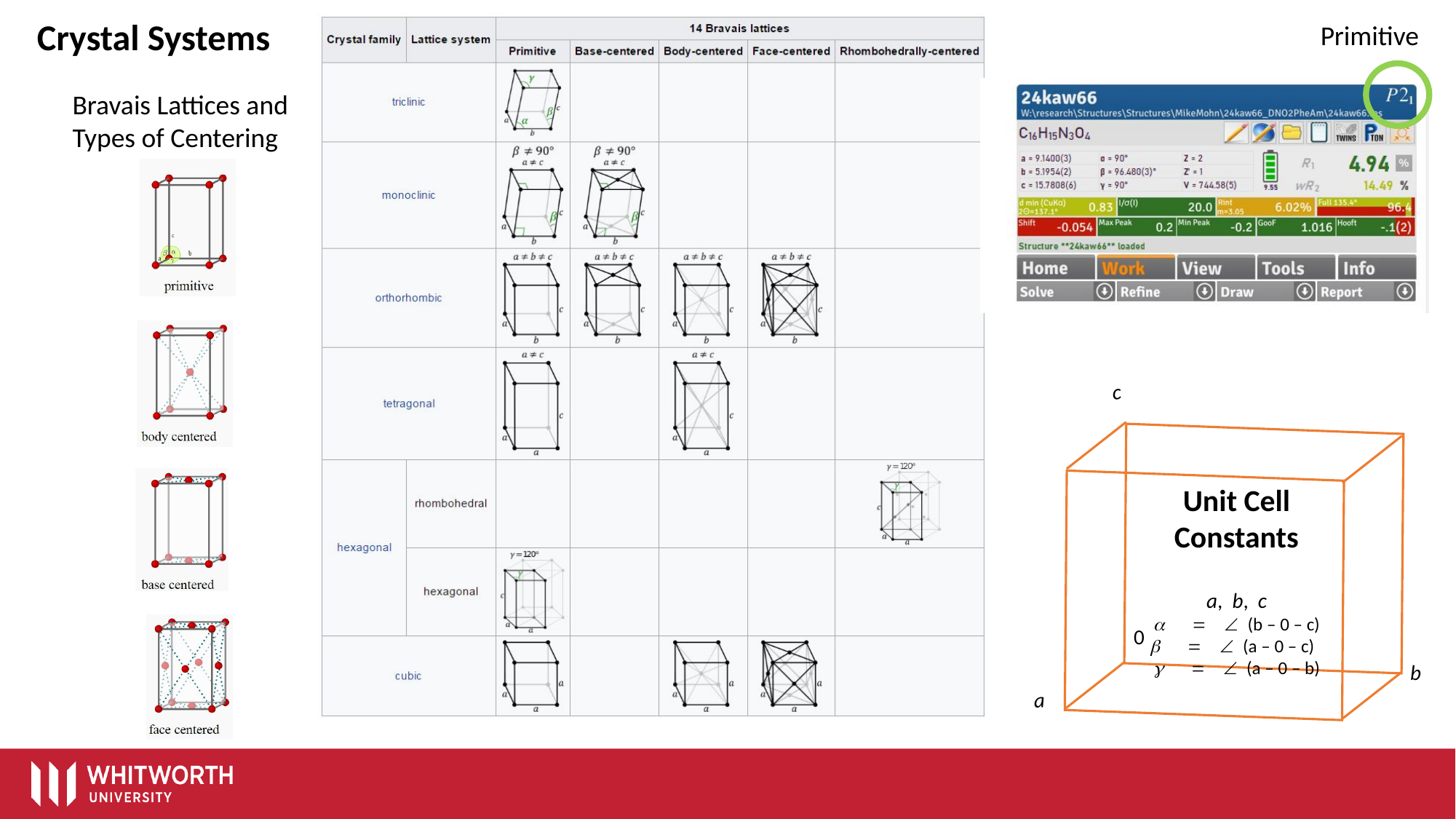

Crystal Systems
Primitive
Bravais Lattices and Types of Centering
c
0
a
b
Unit Cell Constants
a, b, c
a =  (b – 0 – c)
b =  (a – 0 – c)
g =  (a – 0 – b)

## Slide 9
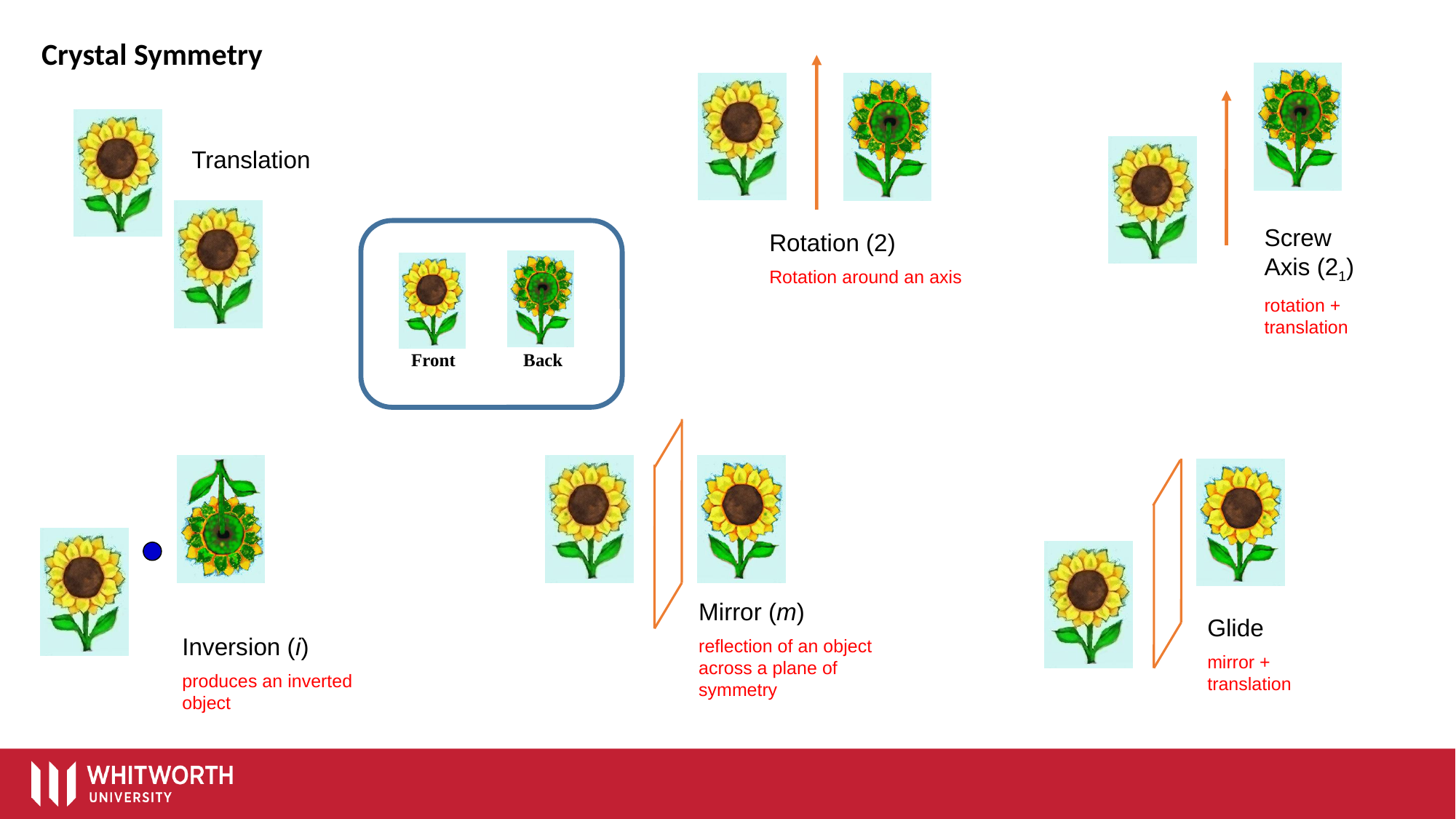

Crystal Symmetry
Translation
Screw Axis (21)
rotation + translation
Rotation (2)
Rotation around an axis
Front
Back
Mirror (m)
reflection of an object across a plane of symmetry
Glide
mirror + translation
Inversion (i)
produces an inverted object

## Slide 10
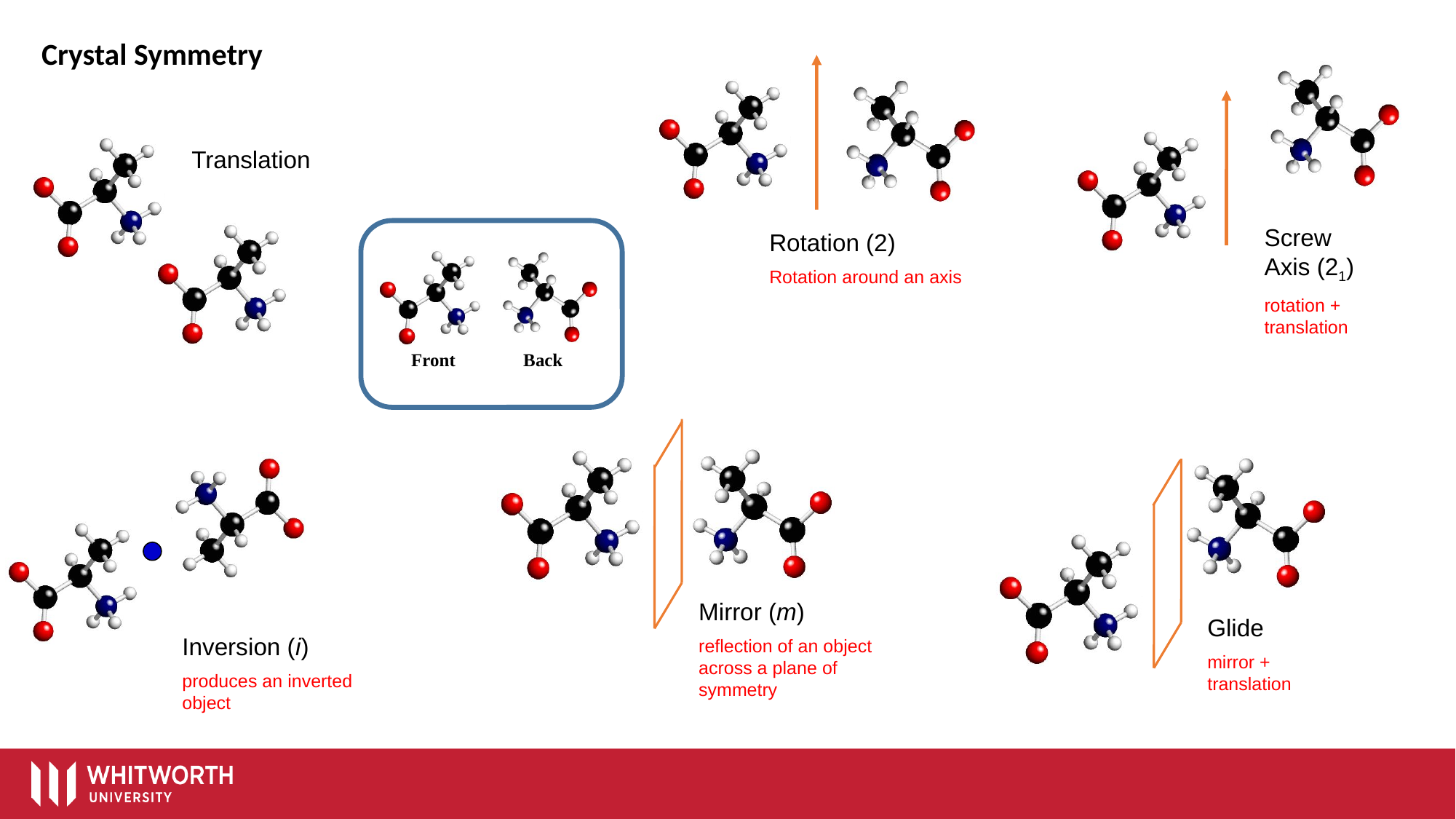

Crystal Symmetry
Translation
Screw Axis (21)
rotation + translation
Rotation (2)
Rotation around an axis
Front
Back
Mirror (m)
reflection of an object across a plane of symmetry
Glide
mirror + translation
Inversion (i)
produces an inverted object

## Slide 11
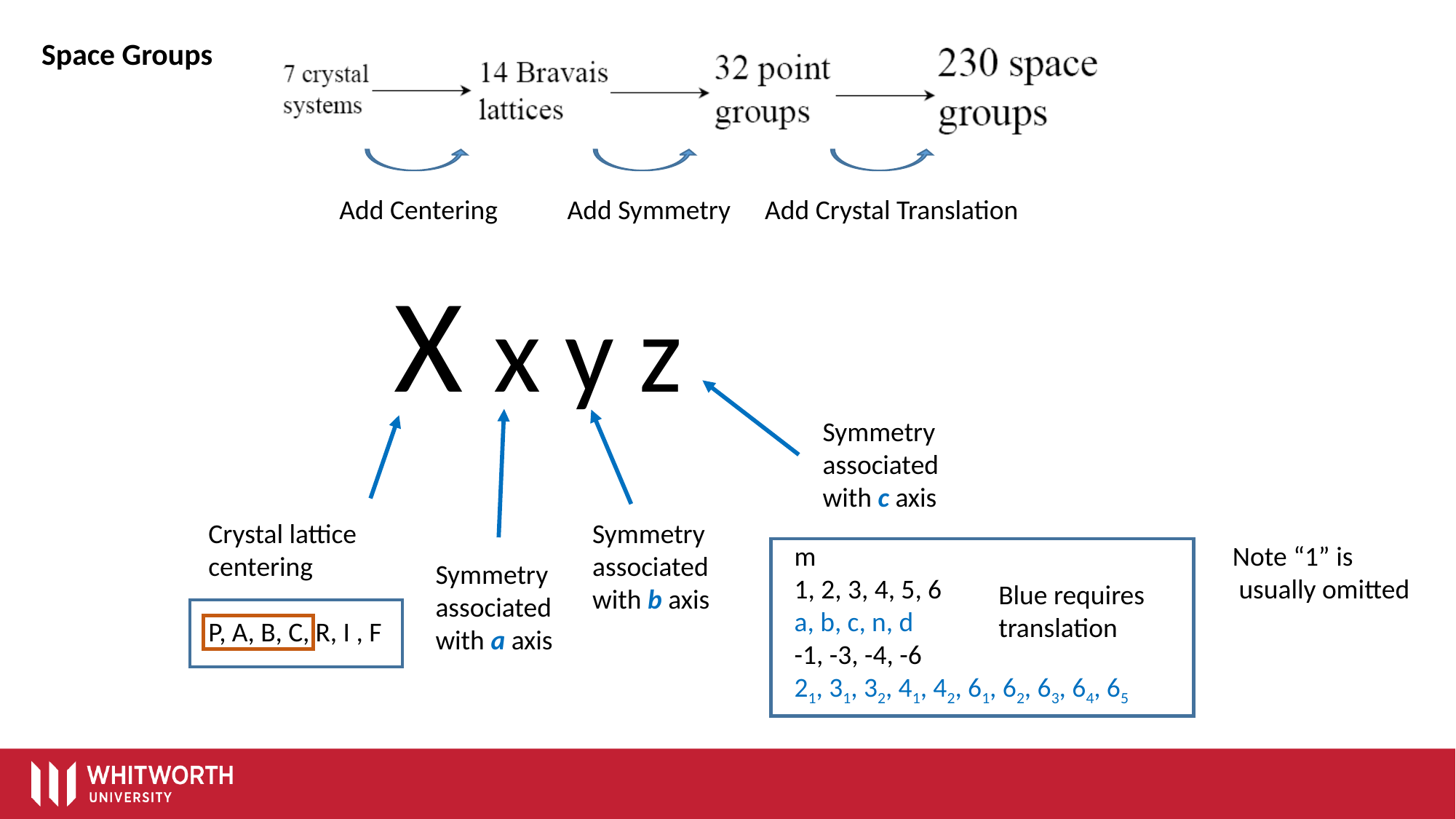

Space Groups
Add Centering
Add Symmetry
Add Crystal Translation
X x y z
Symmetry associated with c axis
Crystal lattice centering
P, A, B, C, R, I , F
Symmetry associated with b axis
m
1, 2, 3, 4, 5, 6
a, b, c, n, d
-1, -3, -4, -6
21, 31, 32, 41, 42, 61, 62, 63, 64, 65
Note “1” is
 usually omitted
Symmetry associated with a axis
Blue requires translation

## Slide 12
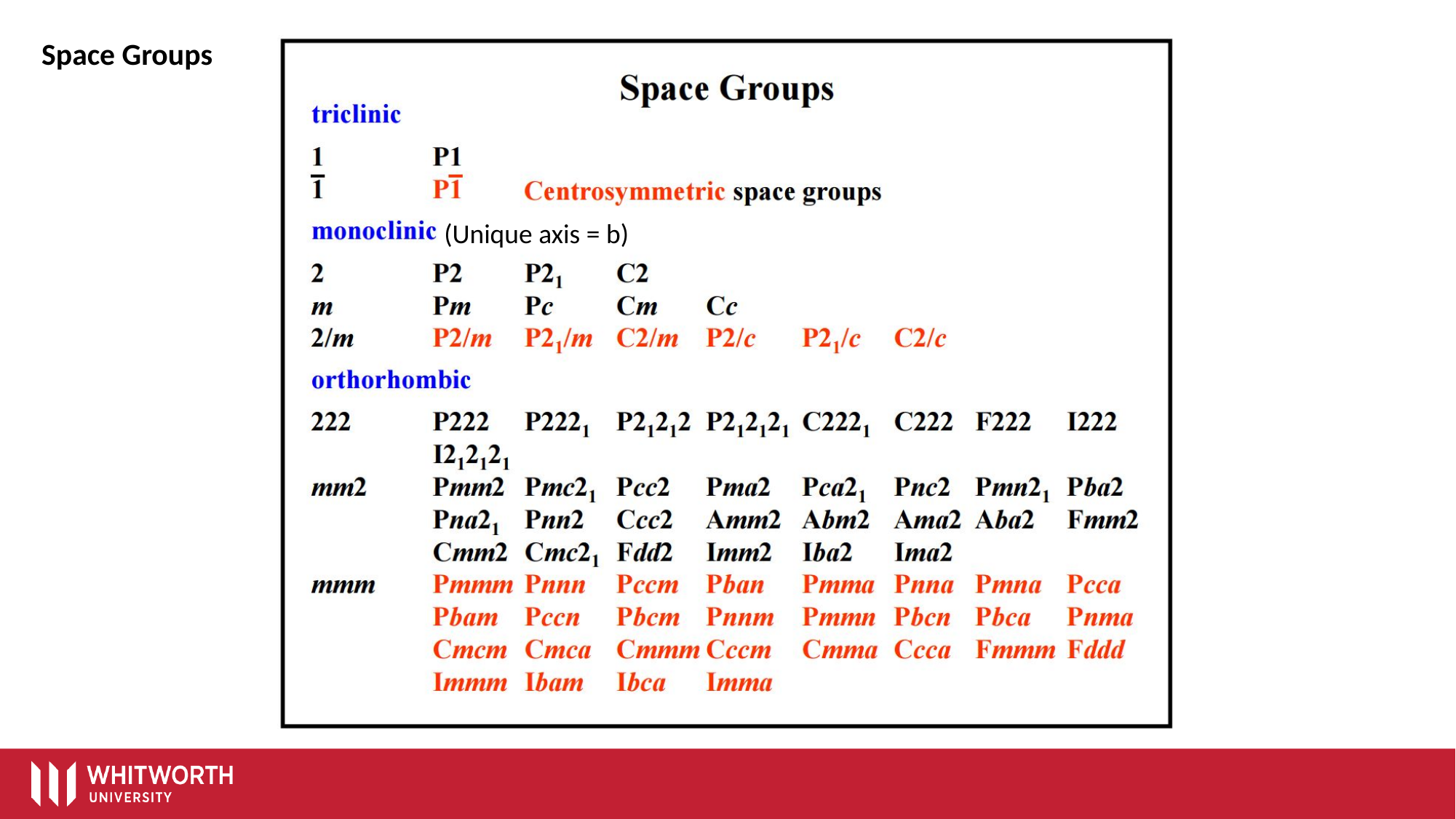

Space Groups
(Unique axis = b)

## Slide 13
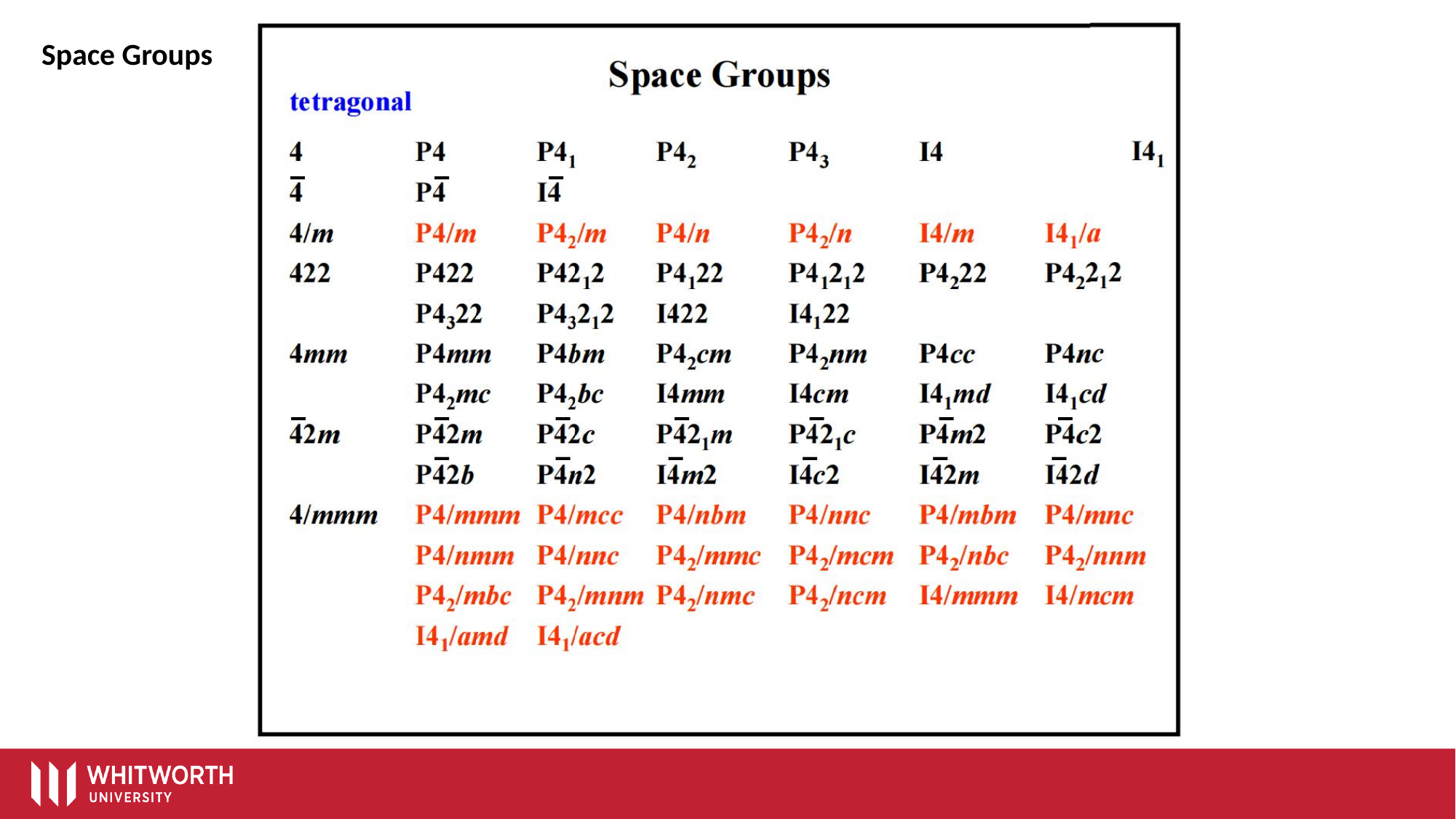

Space Groups

## Slide 14
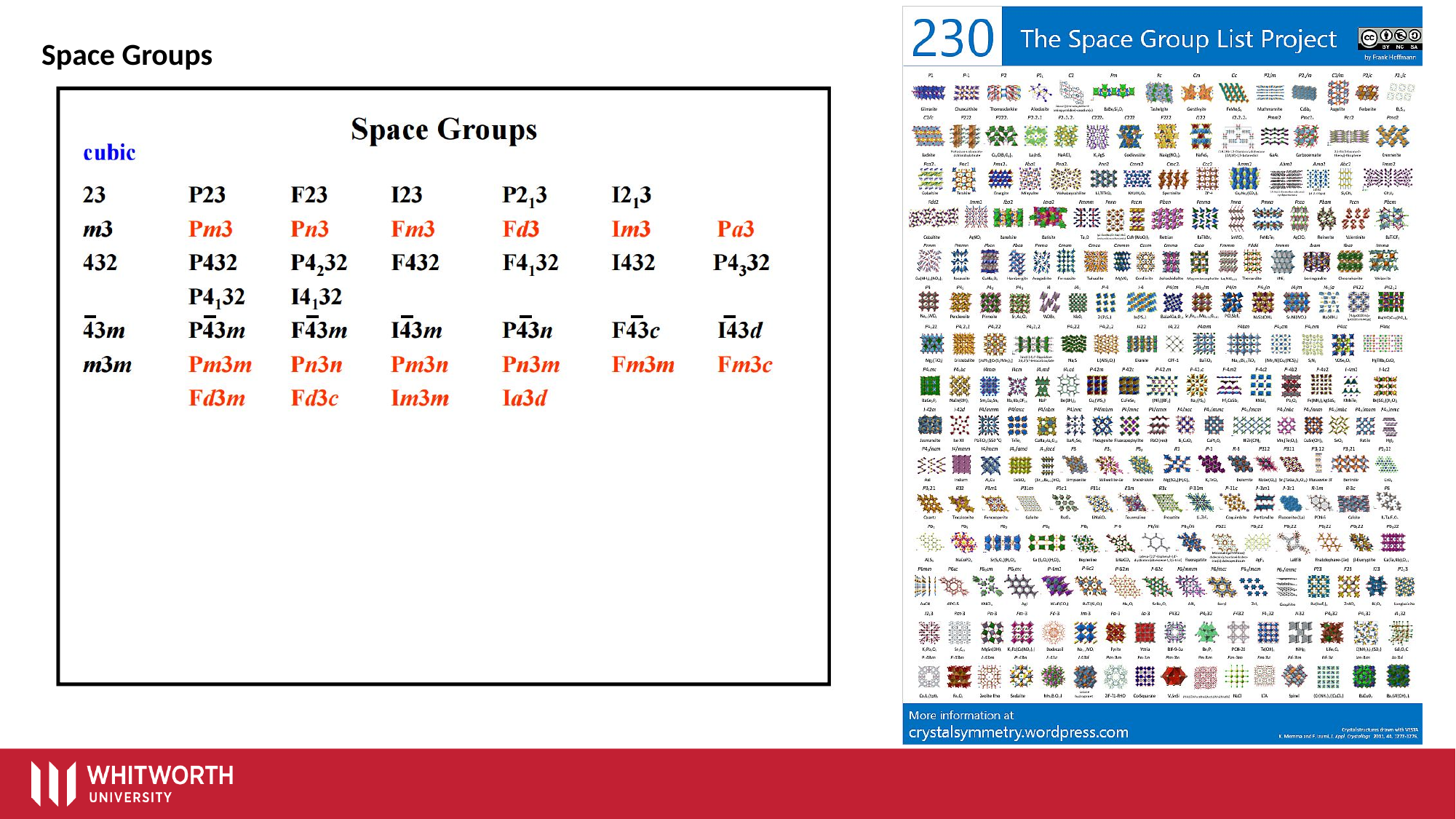

Space Groups

## Slide 15
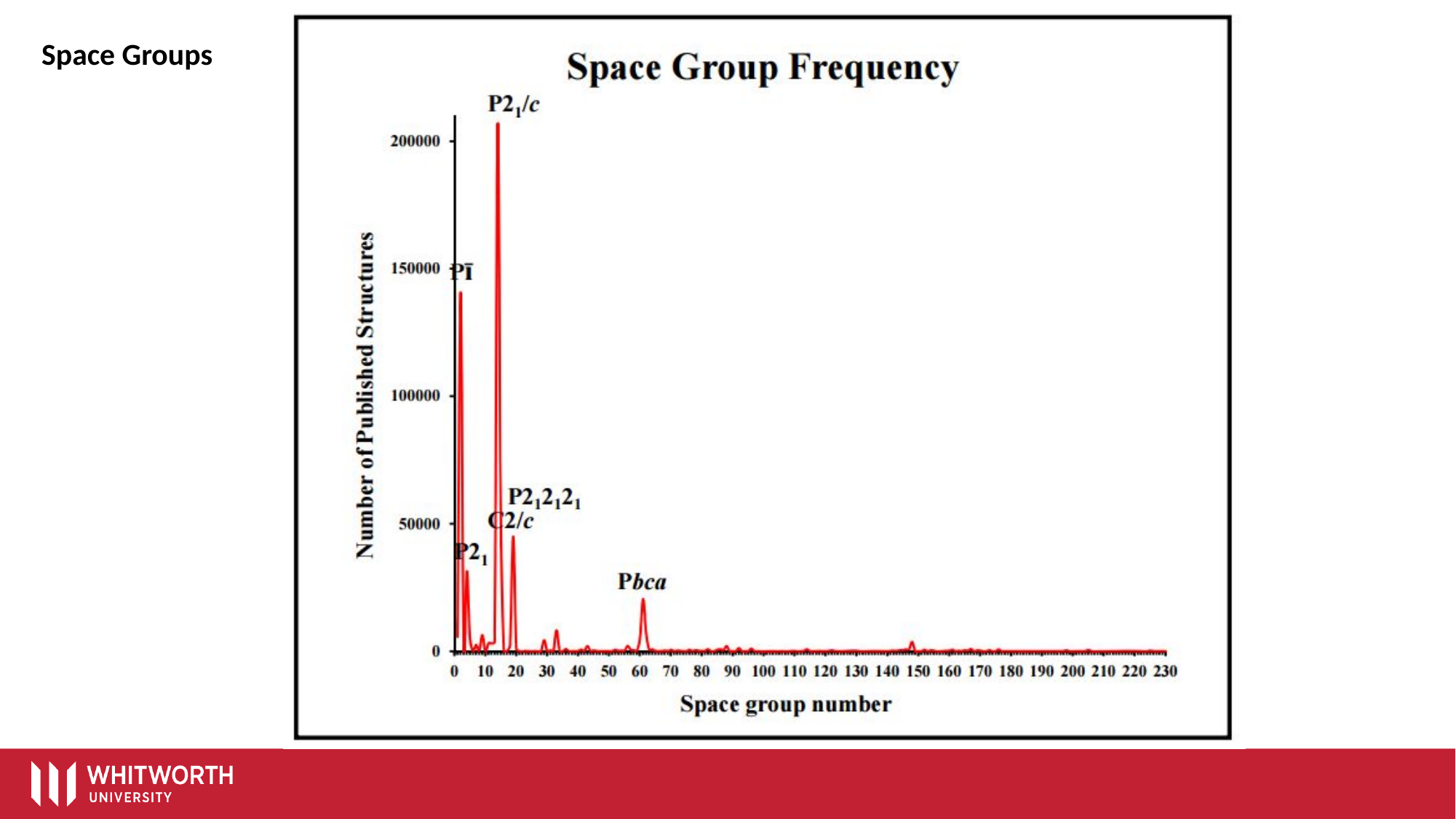

Space Groups

## Slide 16
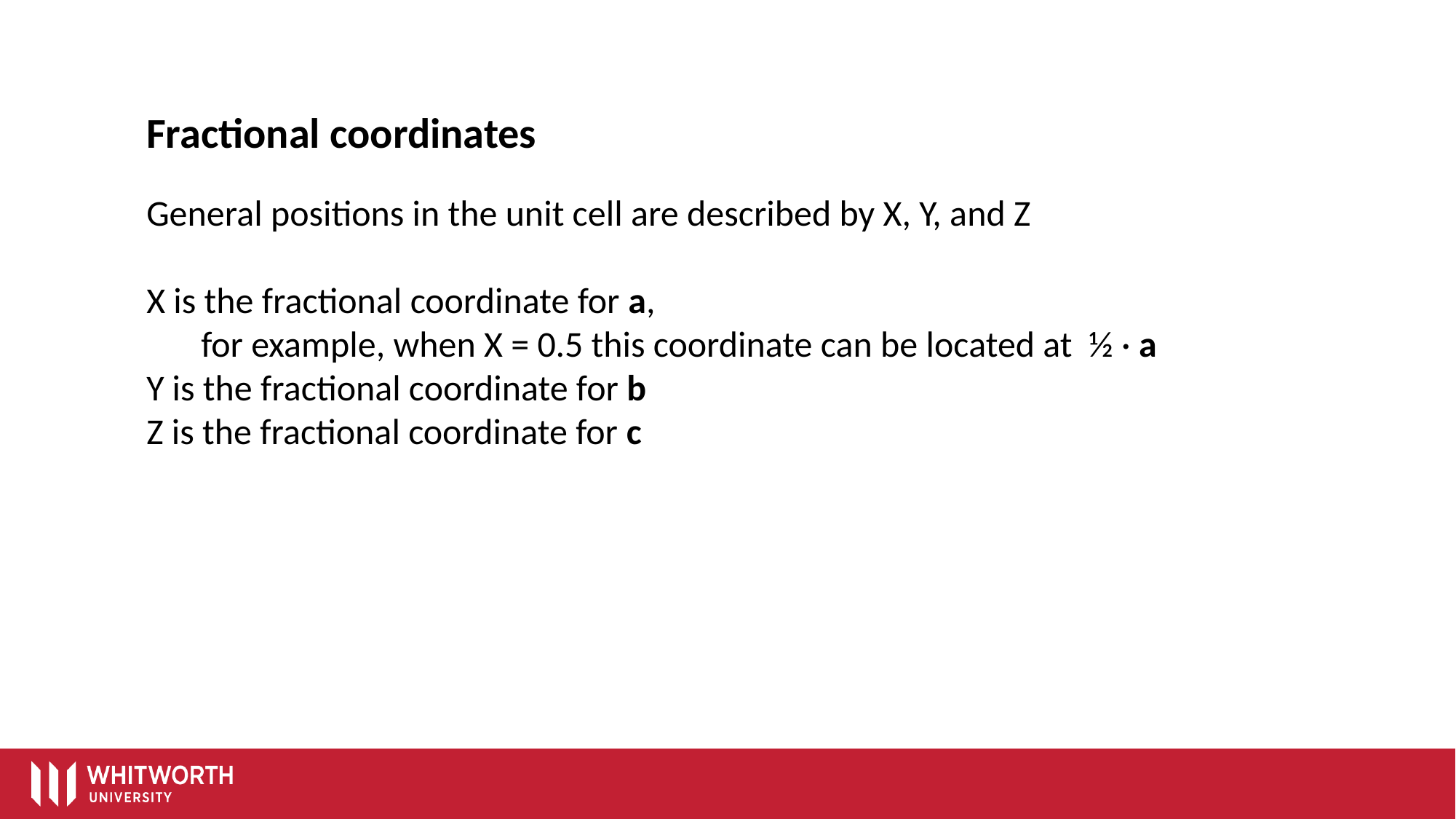

Fractional coordinates
General positions in the unit cell are described by X, Y, and Z
X is the fractional coordinate for a,
	for example, when X = 0.5 this coordinate can be located at ½ · a
Y is the fractional coordinate for b
Z is the fractional coordinate for c

## Slide 17
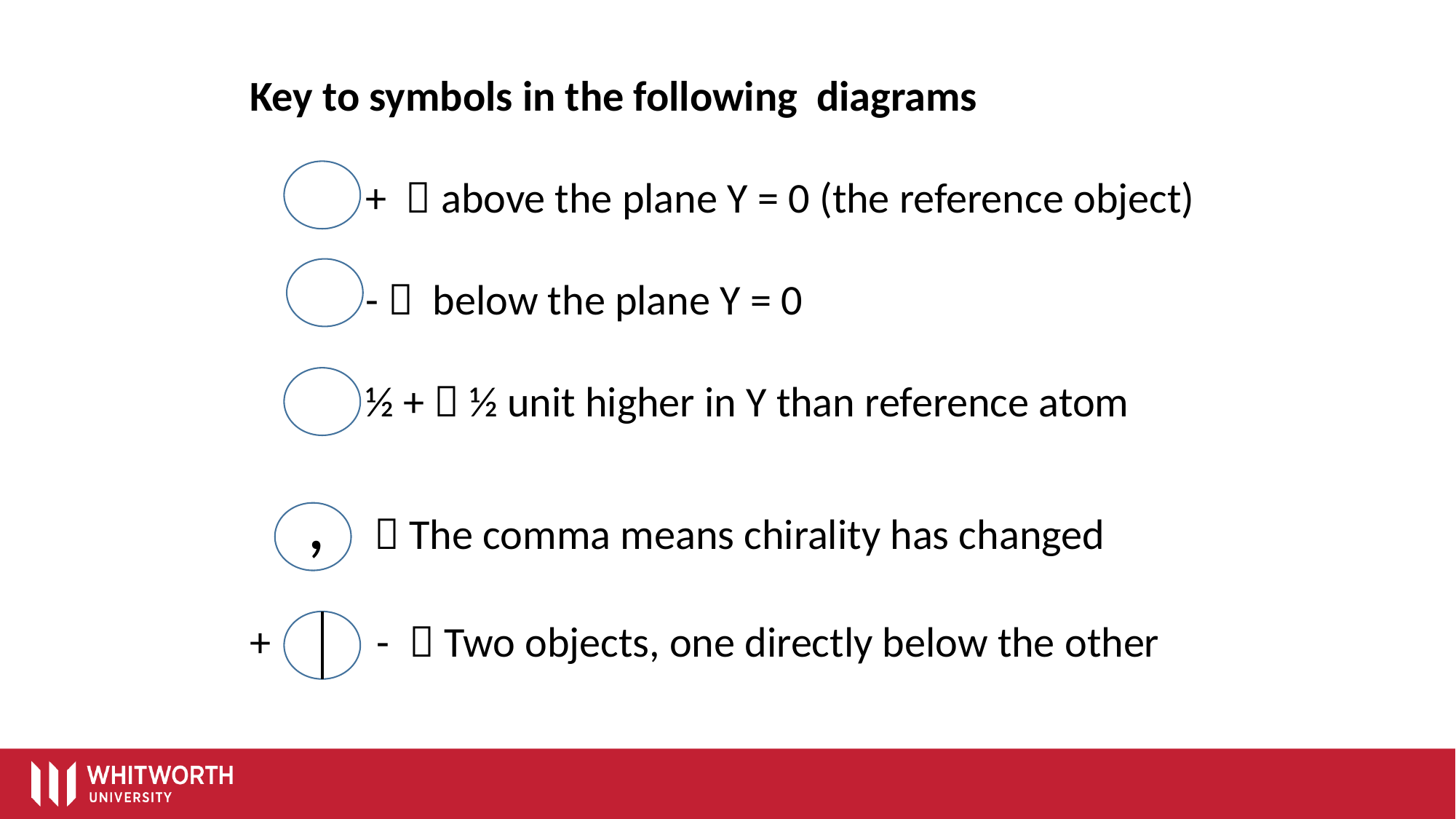

Key to symbols in the following diagrams
 +  above the plane Y = 0 (the reference object)
 -  below the plane Y = 0
 ½ +  ½ unit higher in Y than reference atom
 ,  The comma means chirality has changed
+ -  Two objects, one directly below the other

## Slide 18
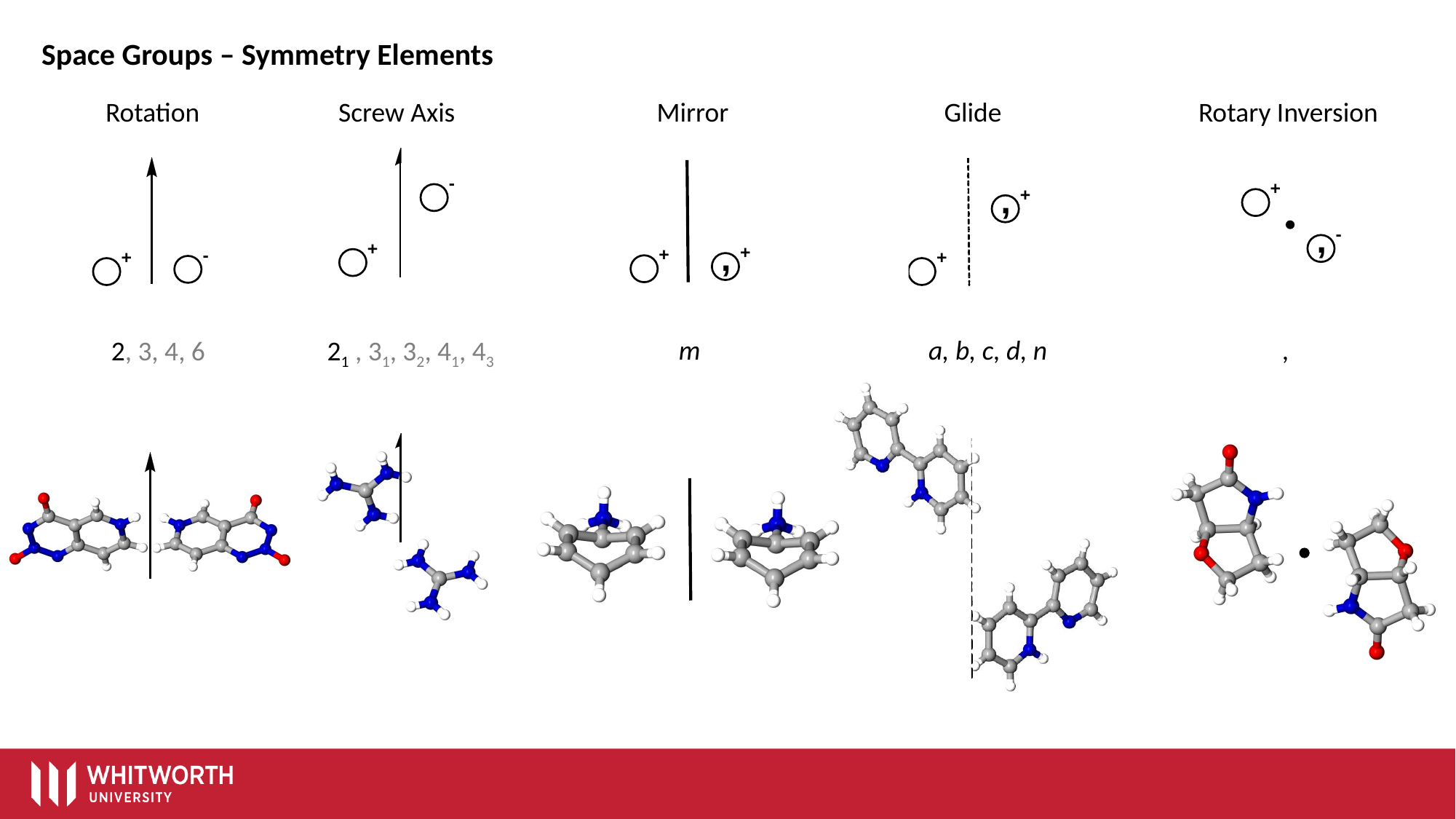

Space Groups – Symmetry Elements
Rotary Inversion
Rotation
Screw Axis
Mirror
Glide
m
a, b, c, d, n
2, 3, 4, 6
21 , 31, 32, 41, 43

## Slide 19
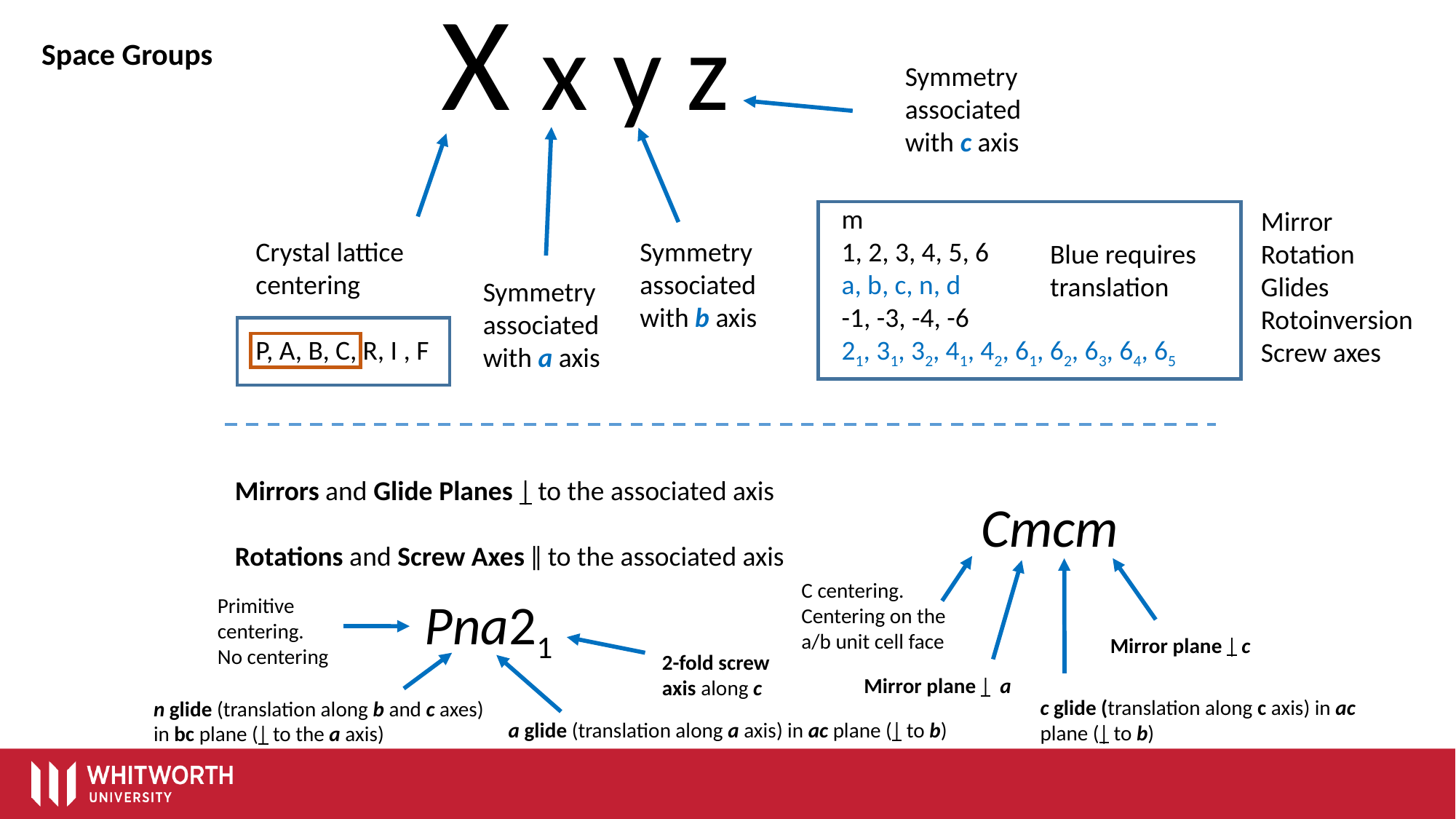

X x y z
Space Groups
Symmetry associated with c axis
m
1, 2, 3, 4, 5, 6
a, b, c, n, d
-1, -3, -4, -6
21, 31, 32, 41, 42, 61, 62, 63, 64, 65
Mirror
Rotation
Glides
Rotoinversion
Screw axes
Crystal lattice centering
P, A, B, C, R, I , F
Symmetry associated with b axis
Blue requires translation
Symmetry associated with a axis
Mirrors and Glide Planes | to the associated axis
Rotations and Screw Axes ǁ to the associated axis
Cmcm
C centering. Centering on the a/b unit cell face
Pna21
Primitive centering. No centering
Mirror plane | c
2-fold screw axis along c
Mirror plane | a
c glide (translation along c axis) in ac plane (| to b)
n glide (translation along b and c axes) in bc plane (| to the a axis)
a glide (translation along a axis) in ac plane (| to b)

## Slide 20
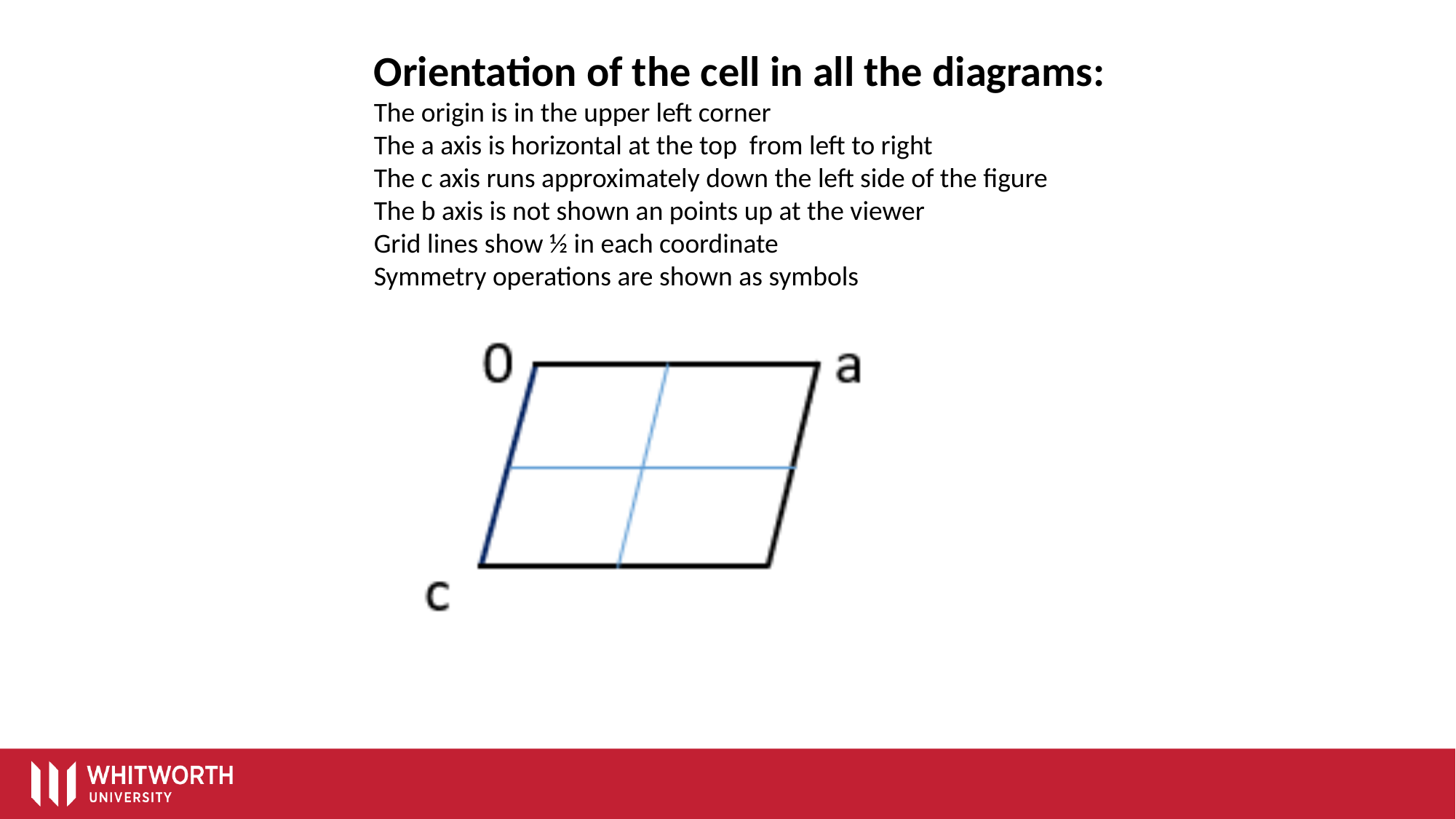

Orientation of the cell in all the diagrams:
The origin is in the upper left corner
The a axis is horizontal at the top from left to right
The c axis runs approximately down the left side of the figure
The b axis is not shown an points up at the viewer
Grid lines show ½ in each coordinate
Symmetry operations are shown as symbols
 0 a
 c

## Slide 21
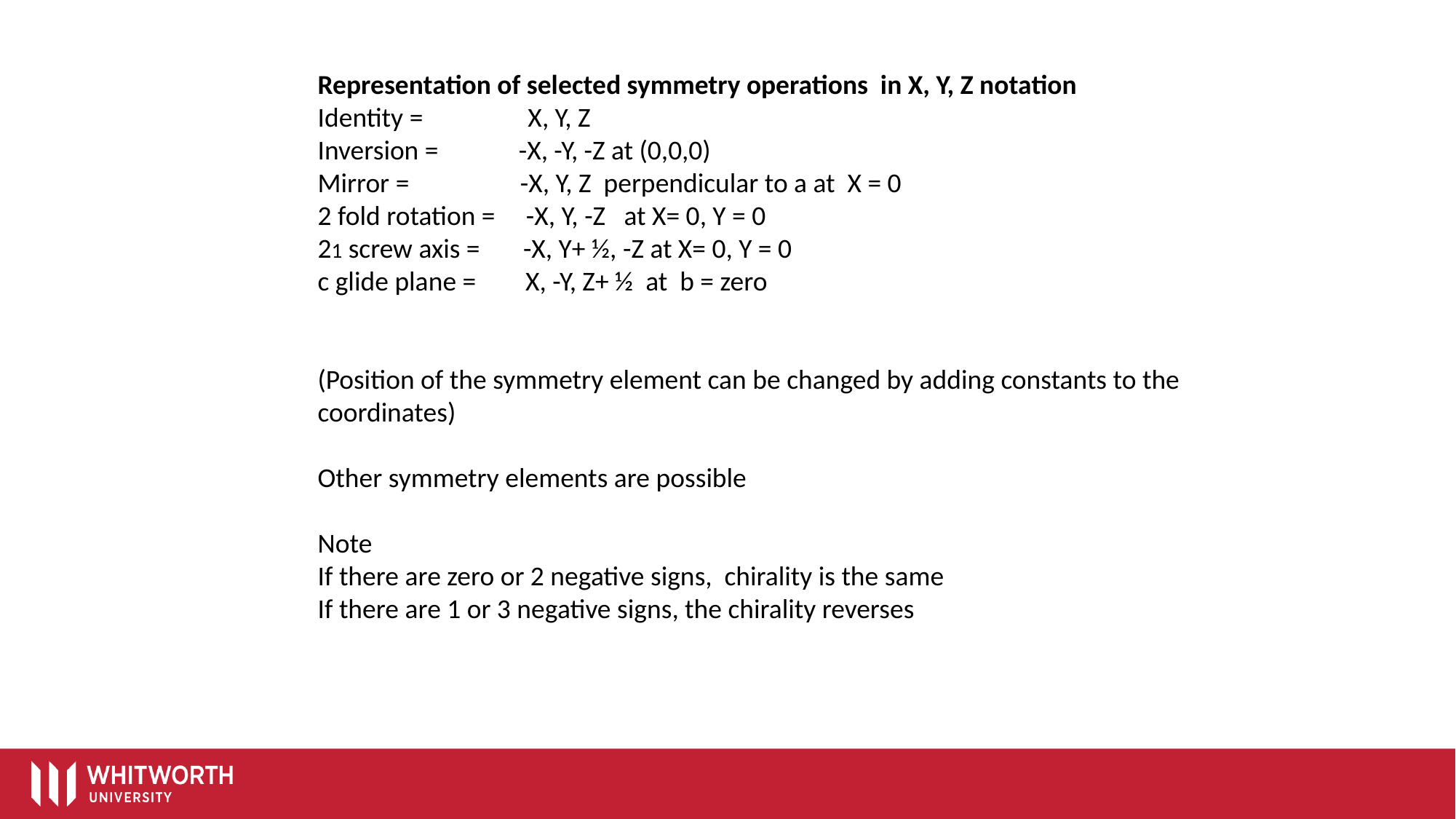

Representation of selected symmetry operations in X, Y, Z notation
Identity = X, Y, Z
Inversion = -X, -Y, -Z at (0,0,0)
Mirror = -X, Y, Z perpendicular to a at X = 0
2 fold rotation = -X, Y, -Z at X= 0, Y = 0
21 screw axis = -X, Y+ ½, -Z at X= 0, Y = 0
c glide plane = X, -Y, Z+ ½ at b = zero
(Position of the symmetry element can be changed by adding constants to the coordinates)
Other symmetry elements are possible
Note
If there are zero or 2 negative signs, chirality is the same
If there are 1 or 3 negative signs, the chirality reverses

## Slide 22
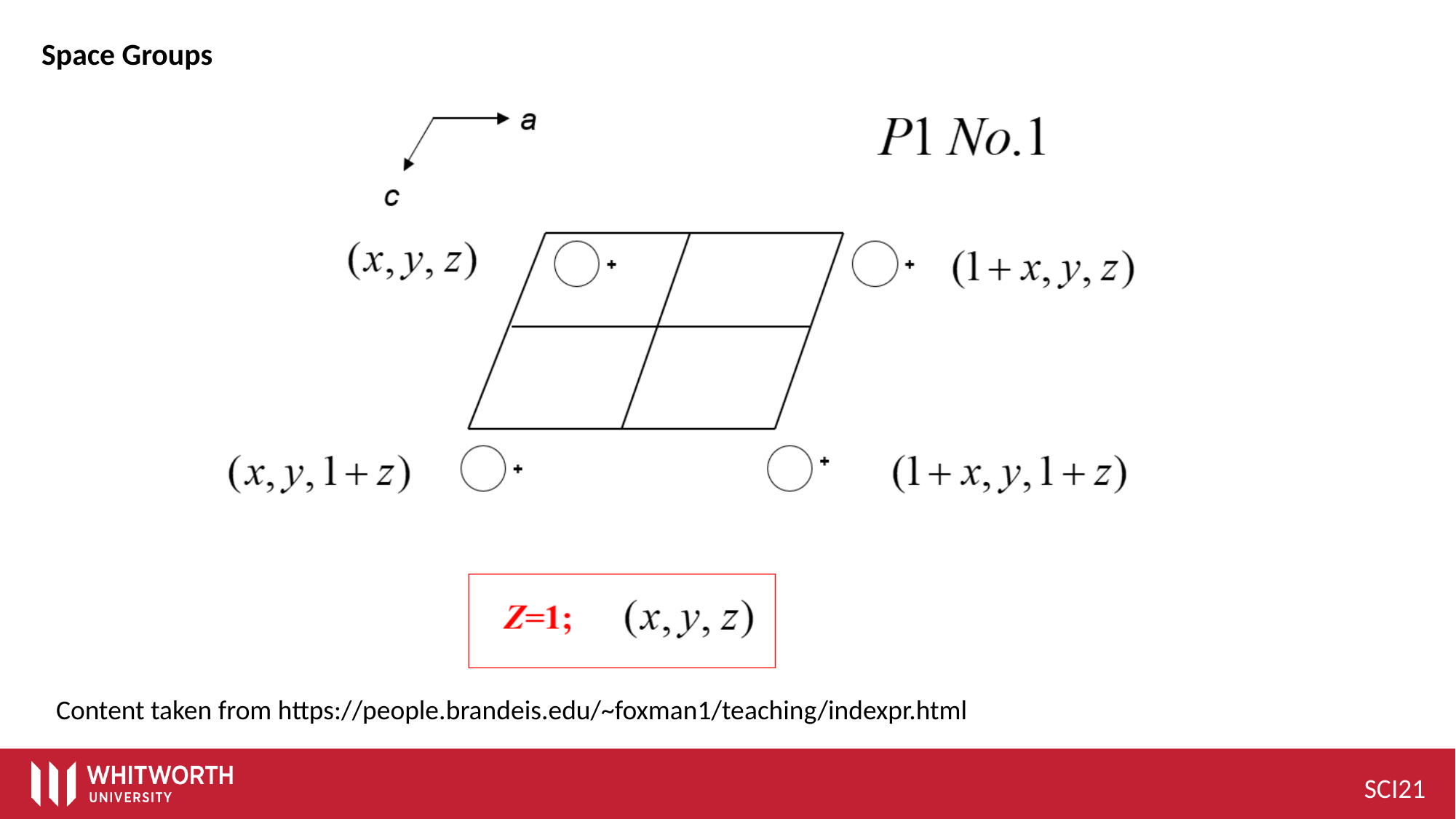

Space Groups
Content taken from https://people.brandeis.edu/~foxman1/teaching/indexpr.html
SCI21

## Slide 23
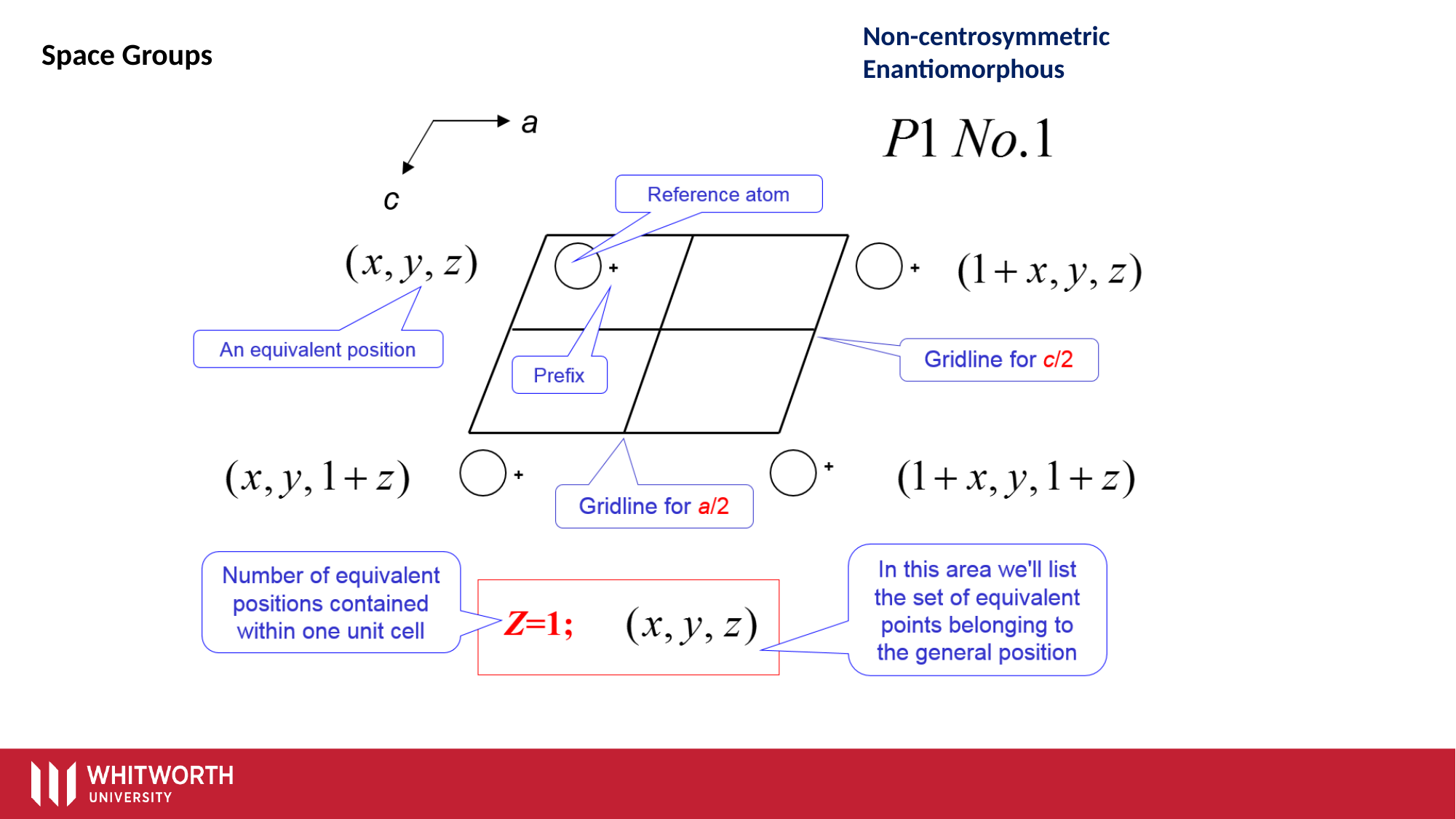

Non-centrosymmetric
Enantiomorphous
Space Groups

## Slide 24
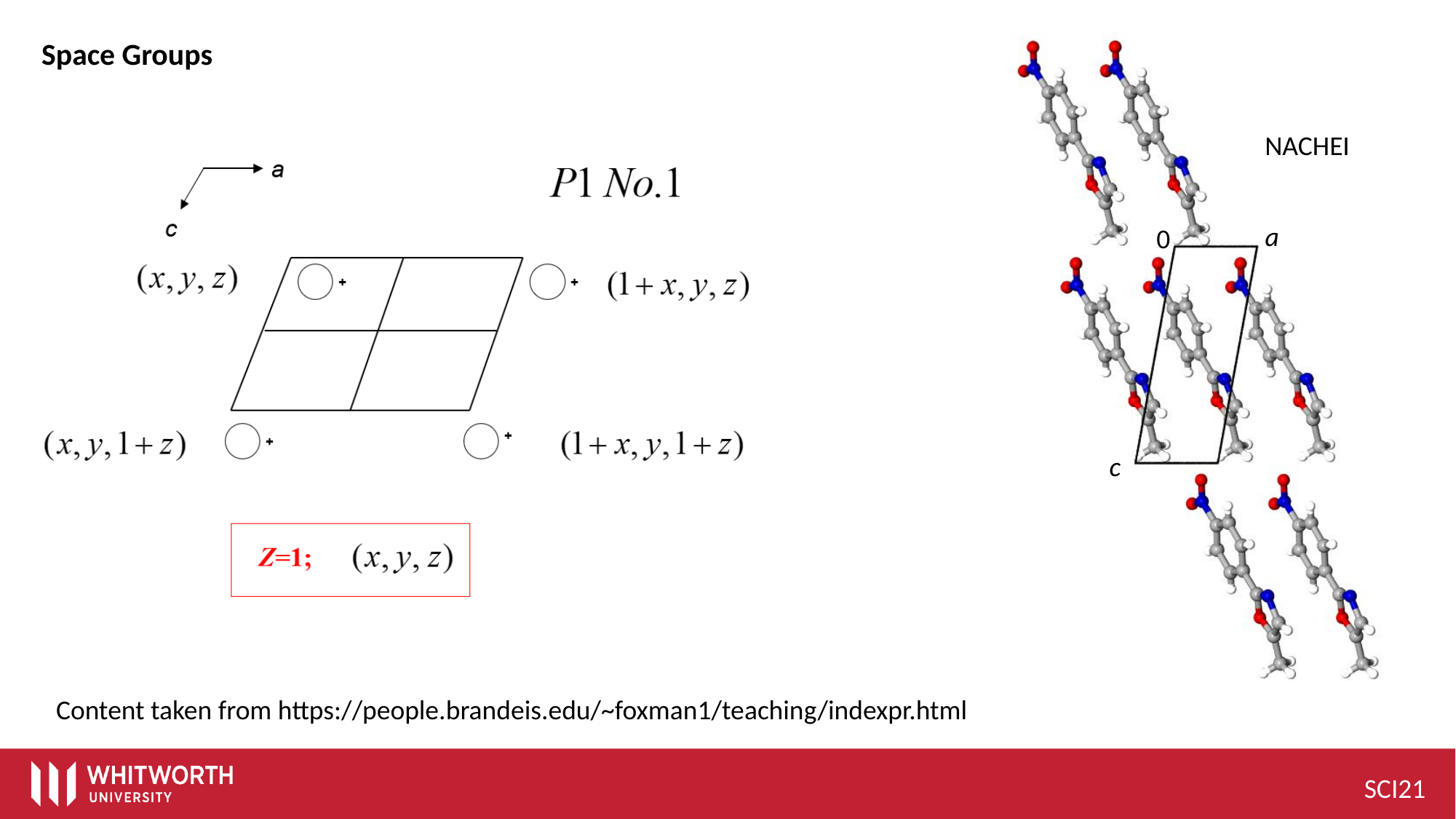

Space Groups
NACHEI
a
0
c
Content taken from https://people.brandeis.edu/~foxman1/teaching/indexpr.html
SCI21

## Slide 25
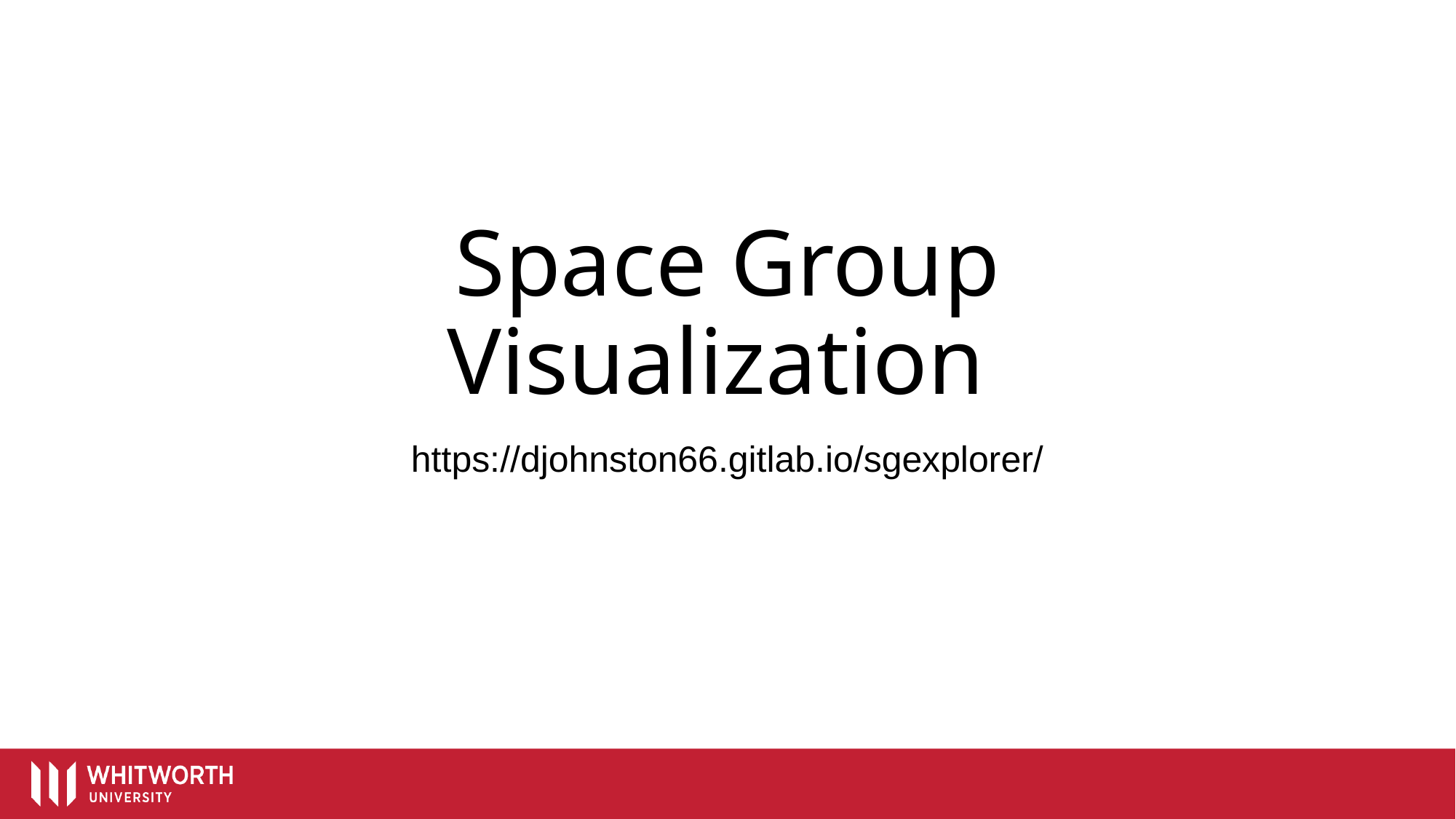

# Space Group Visualization
https://djohnston66.gitlab.io/sgexplorer/

## Slide 26
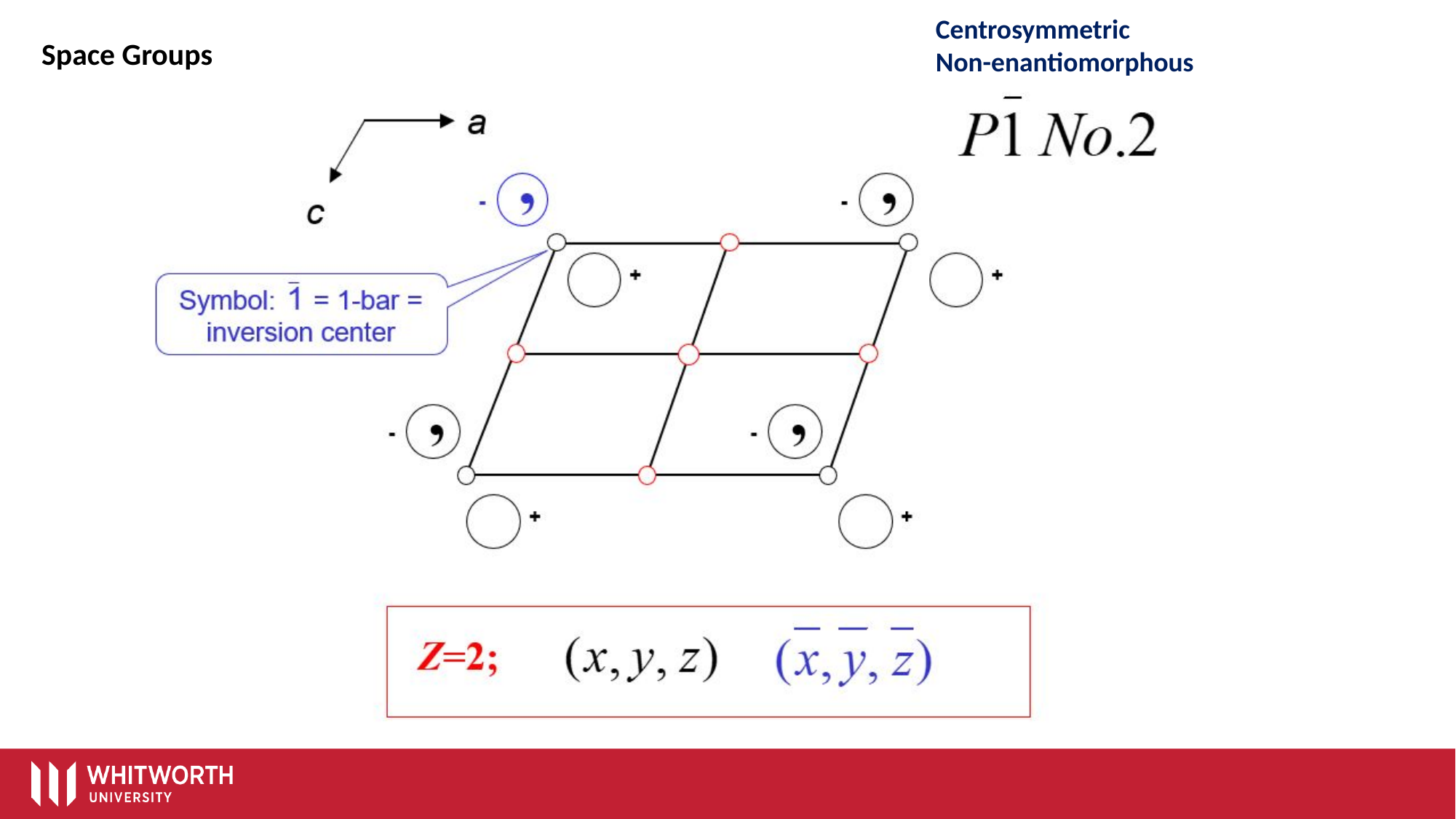

Centrosymmetric
Non-enantiomorphous
Space Groups

## Slide 27
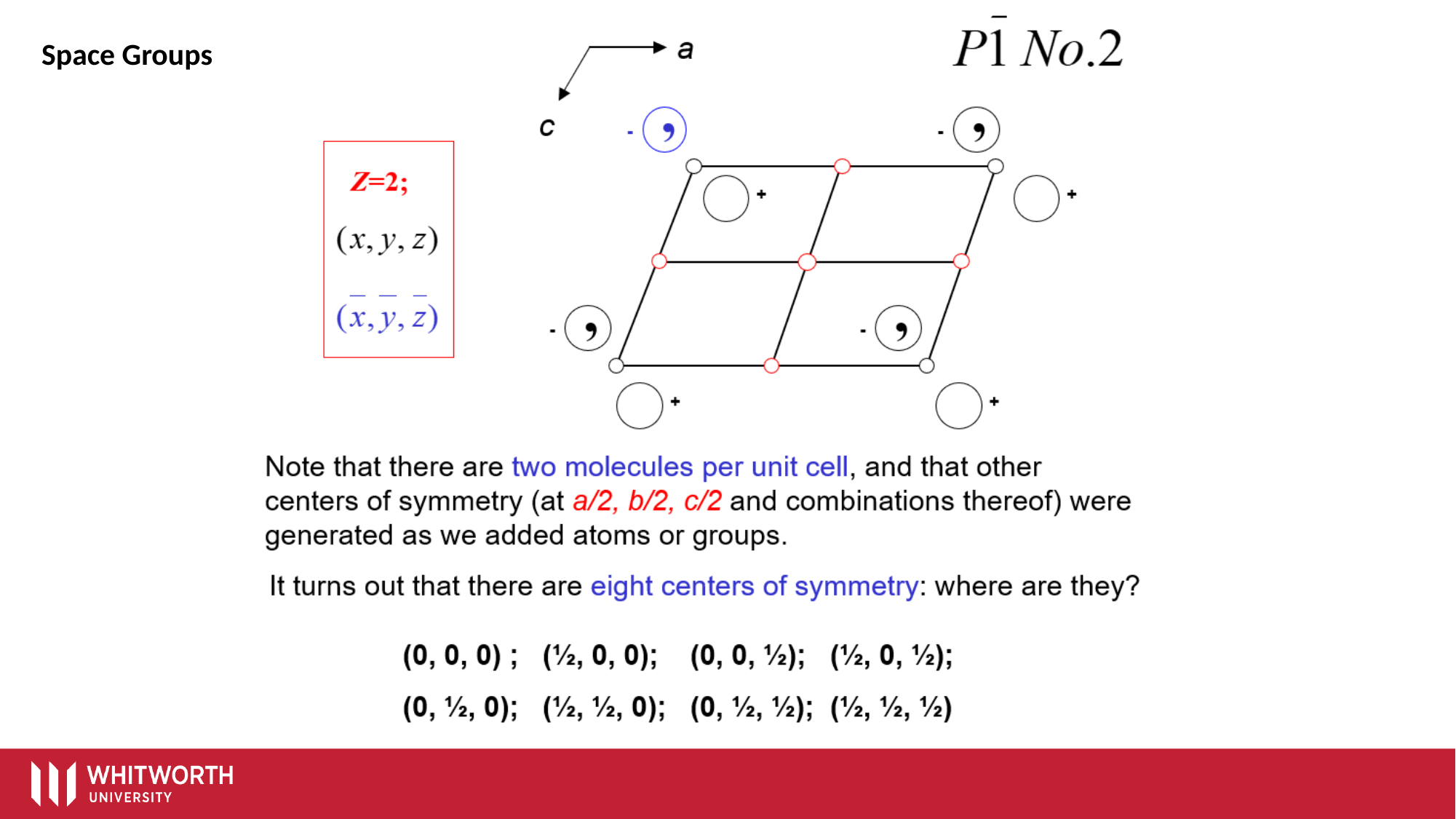

Space Groups

## Slide 28
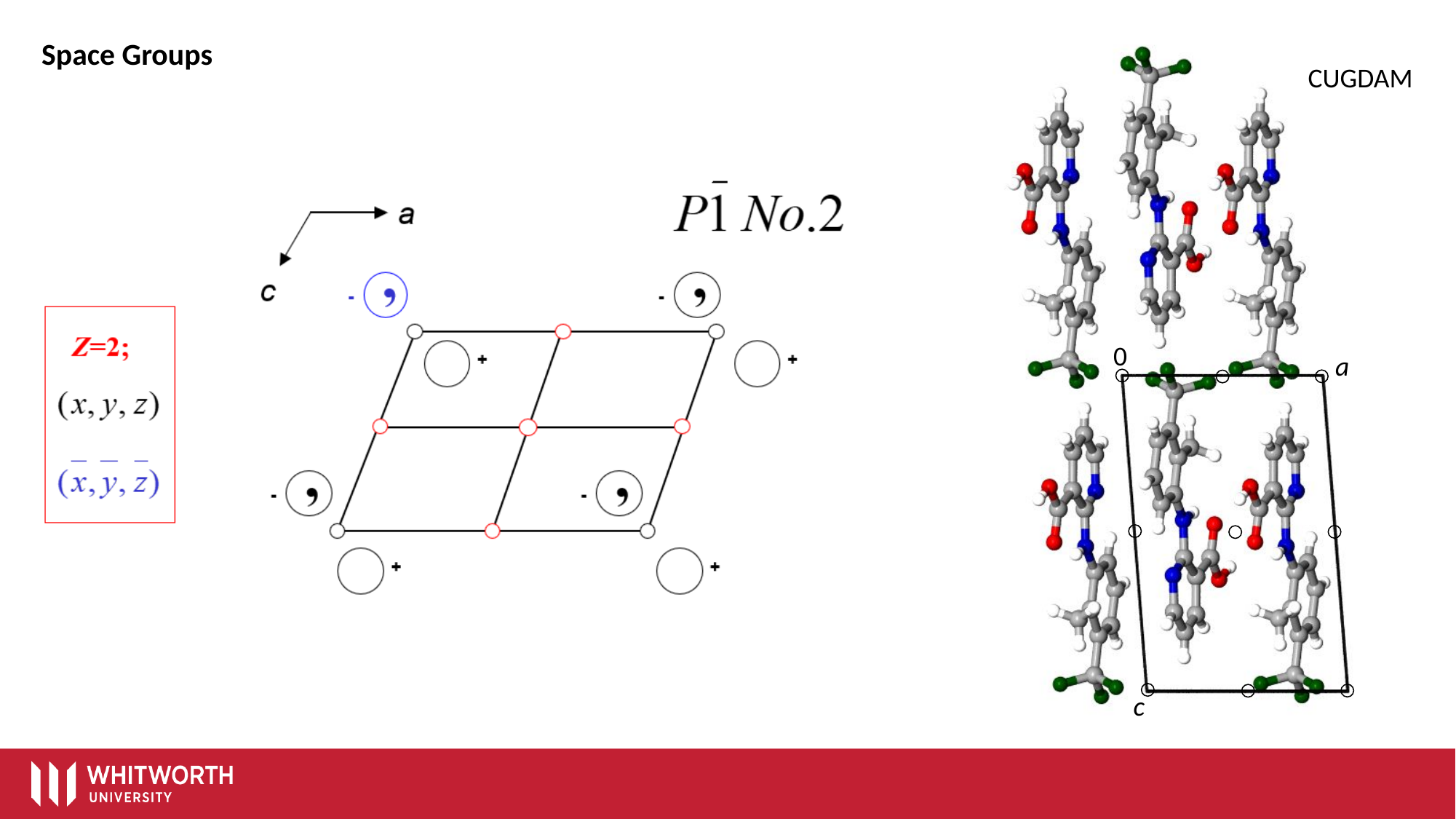

Space Groups
CUGDAM
0
a
c

## Slide 29
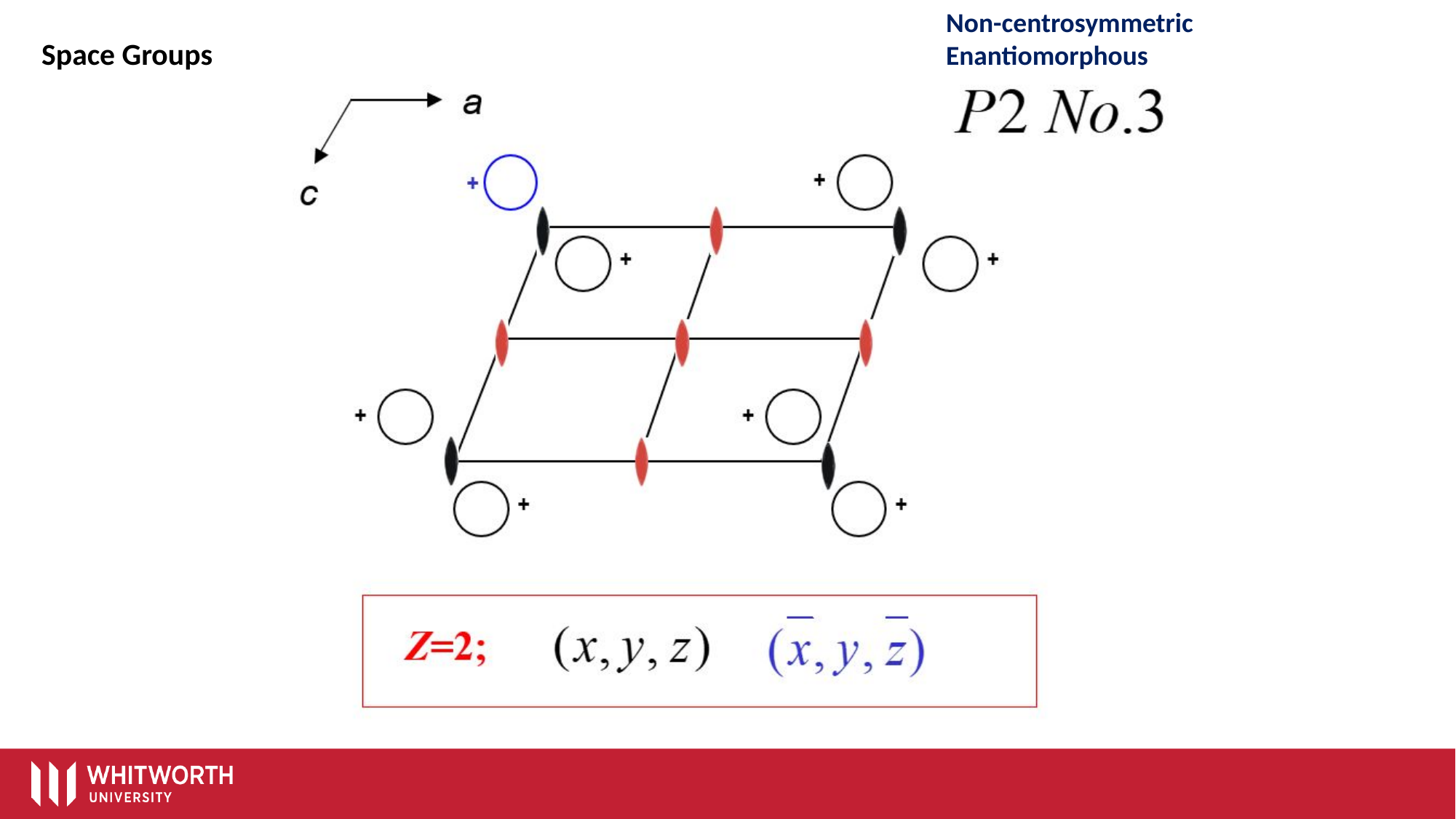

Non-centrosymmetric
Enantiomorphous
Space Groups

## Slide 30
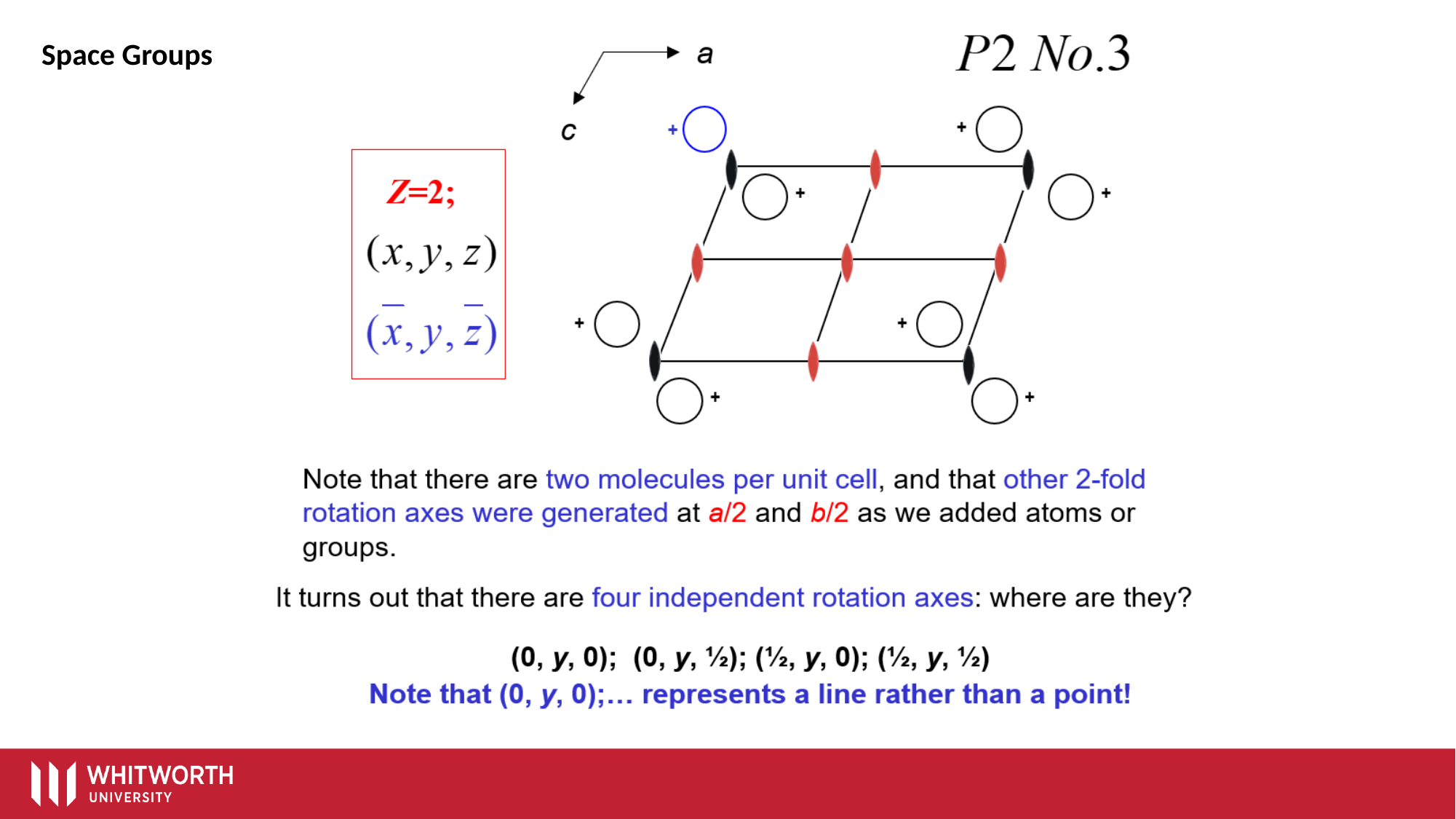

Space Groups

## Slide 31
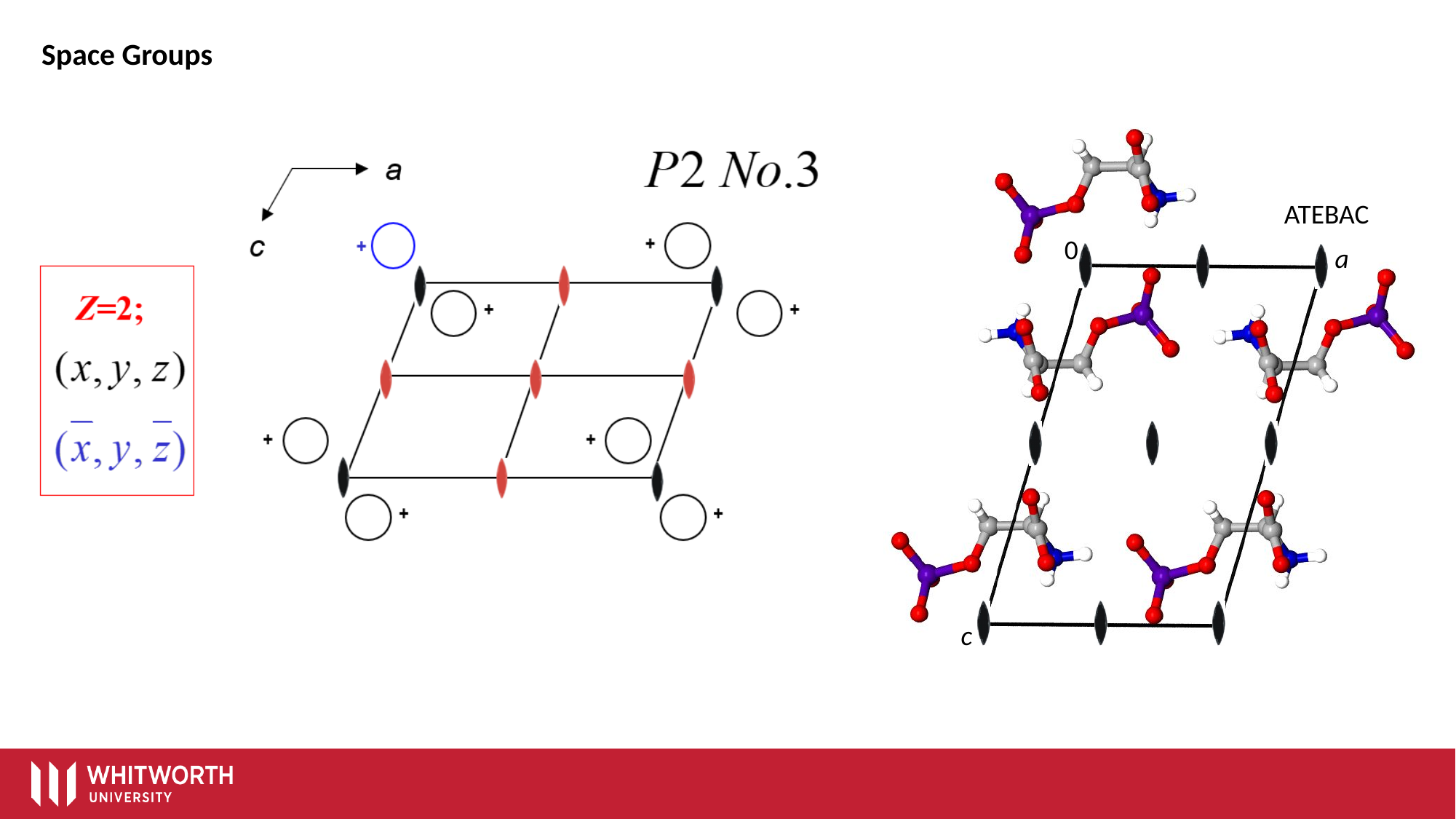

Space Groups
ATEBAC
0
a
c

## Slide 32
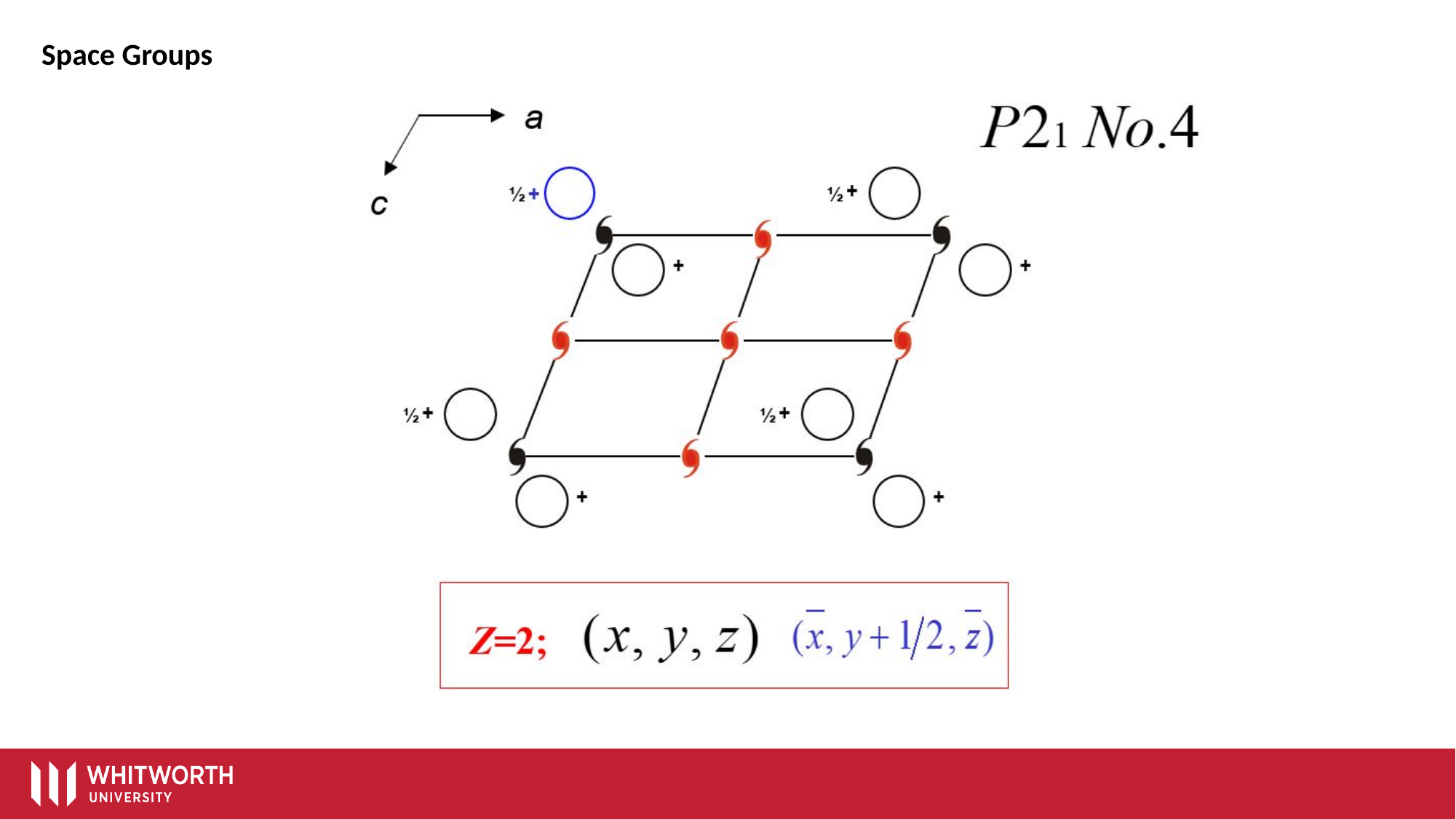

Space Groups

## Slide 33
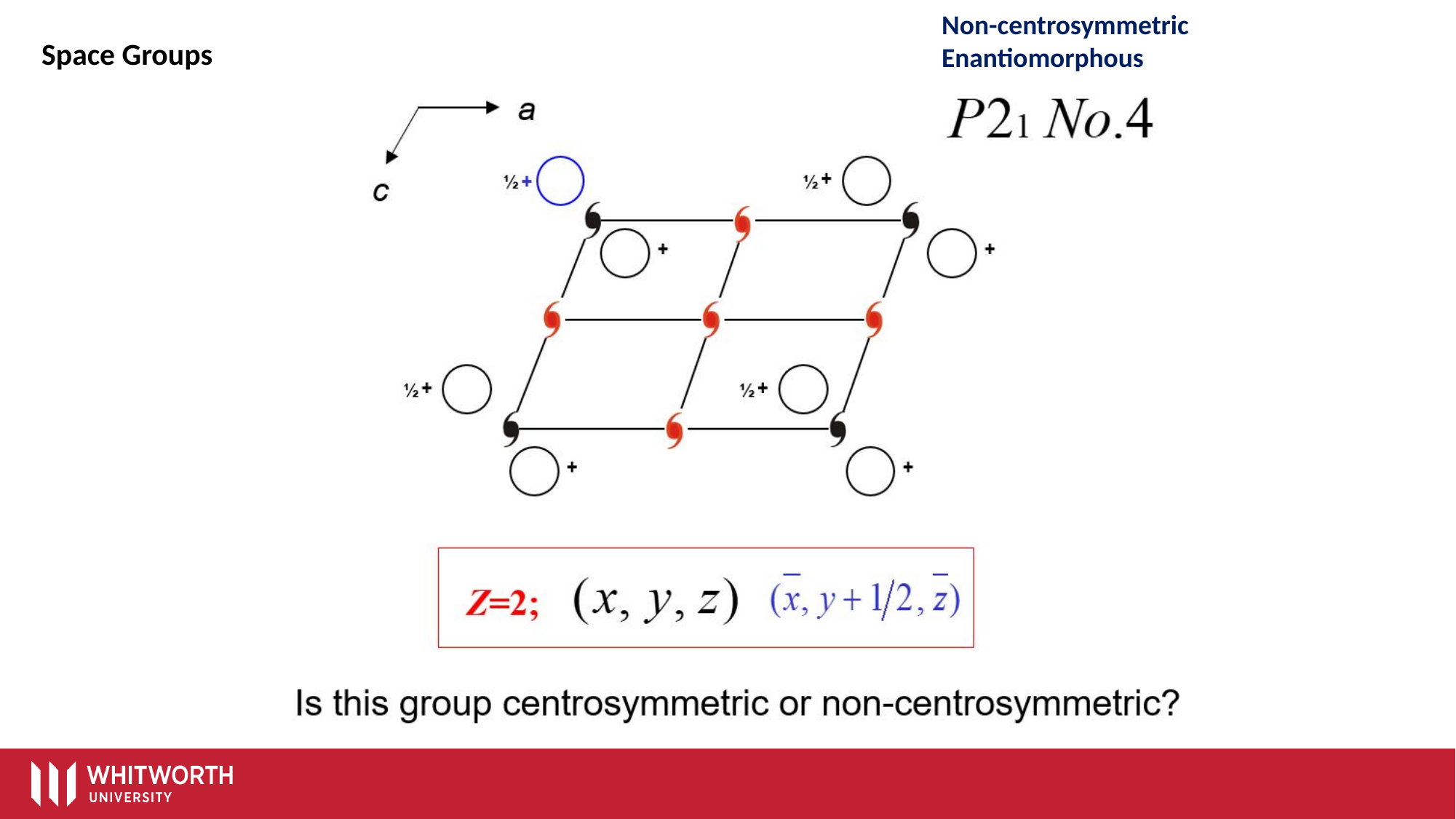

Non-centrosymmetric
Enantiomorphous
Space Groups

## Slide 34
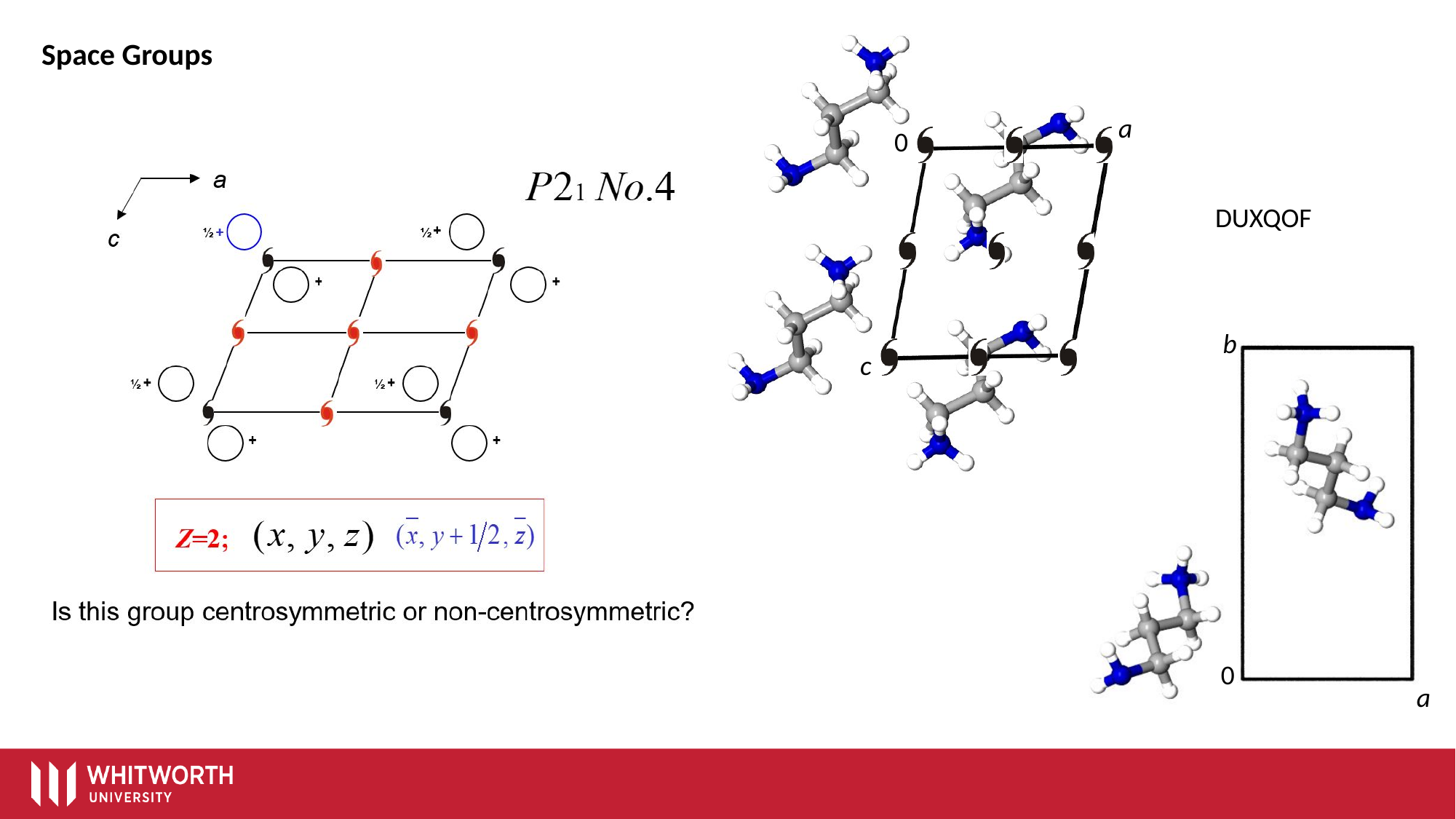

Space Groups
a
0
DUXQOF
b
c
0
a

## Slide 35
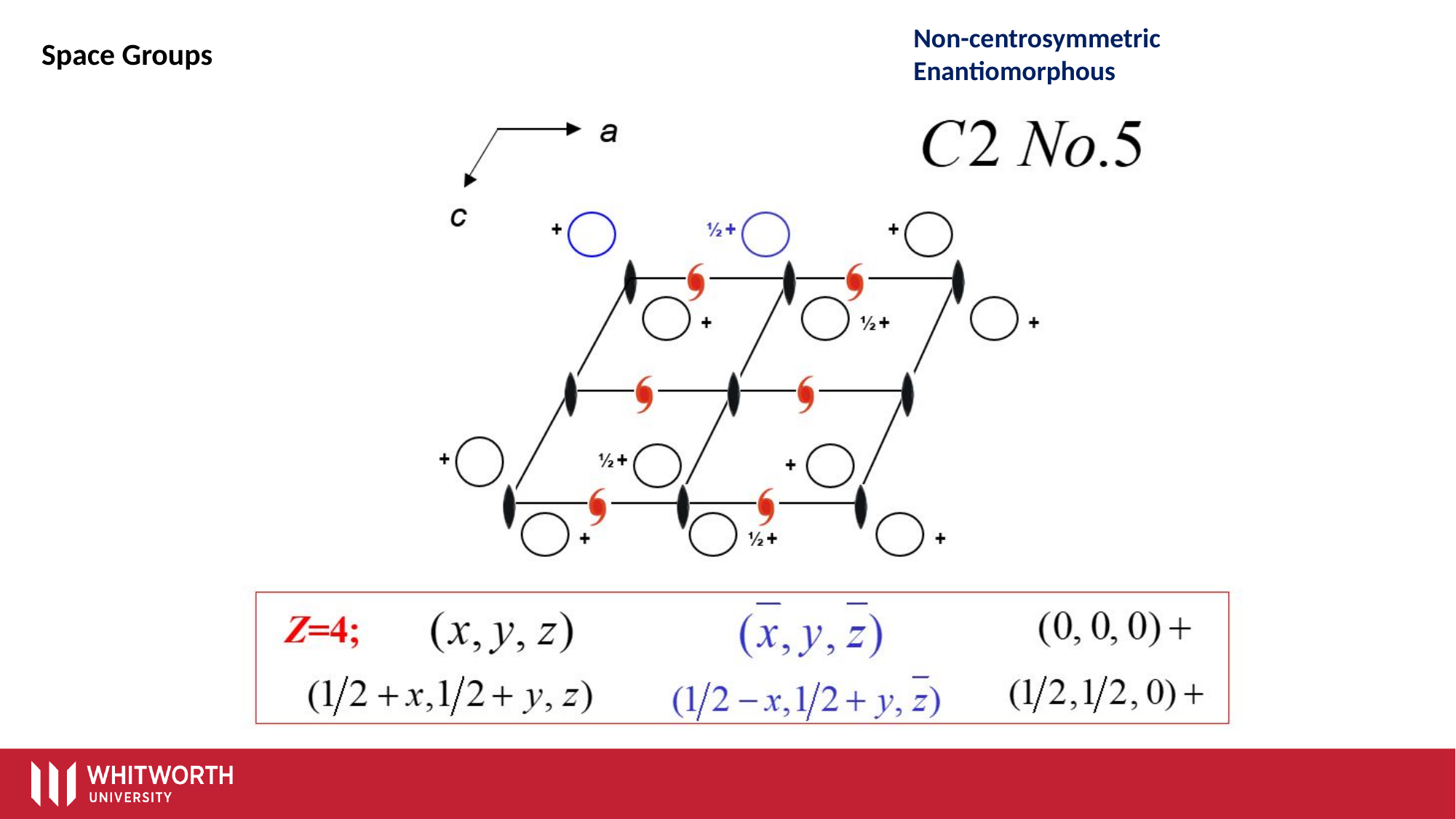

Non-centrosymmetric
Enantiomorphous
Space Groups

## Slide 36
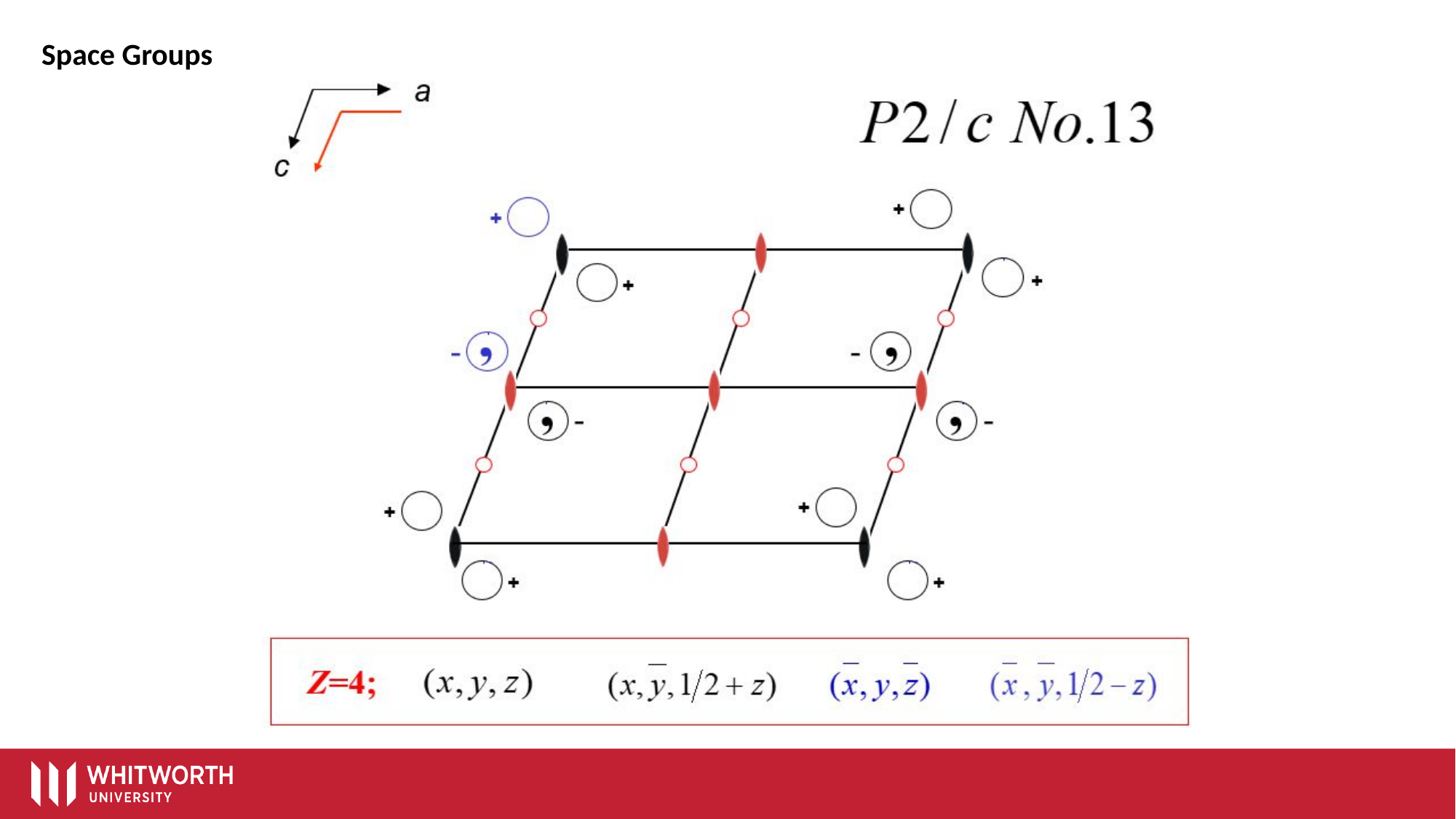

Space Groups

## Slide 37
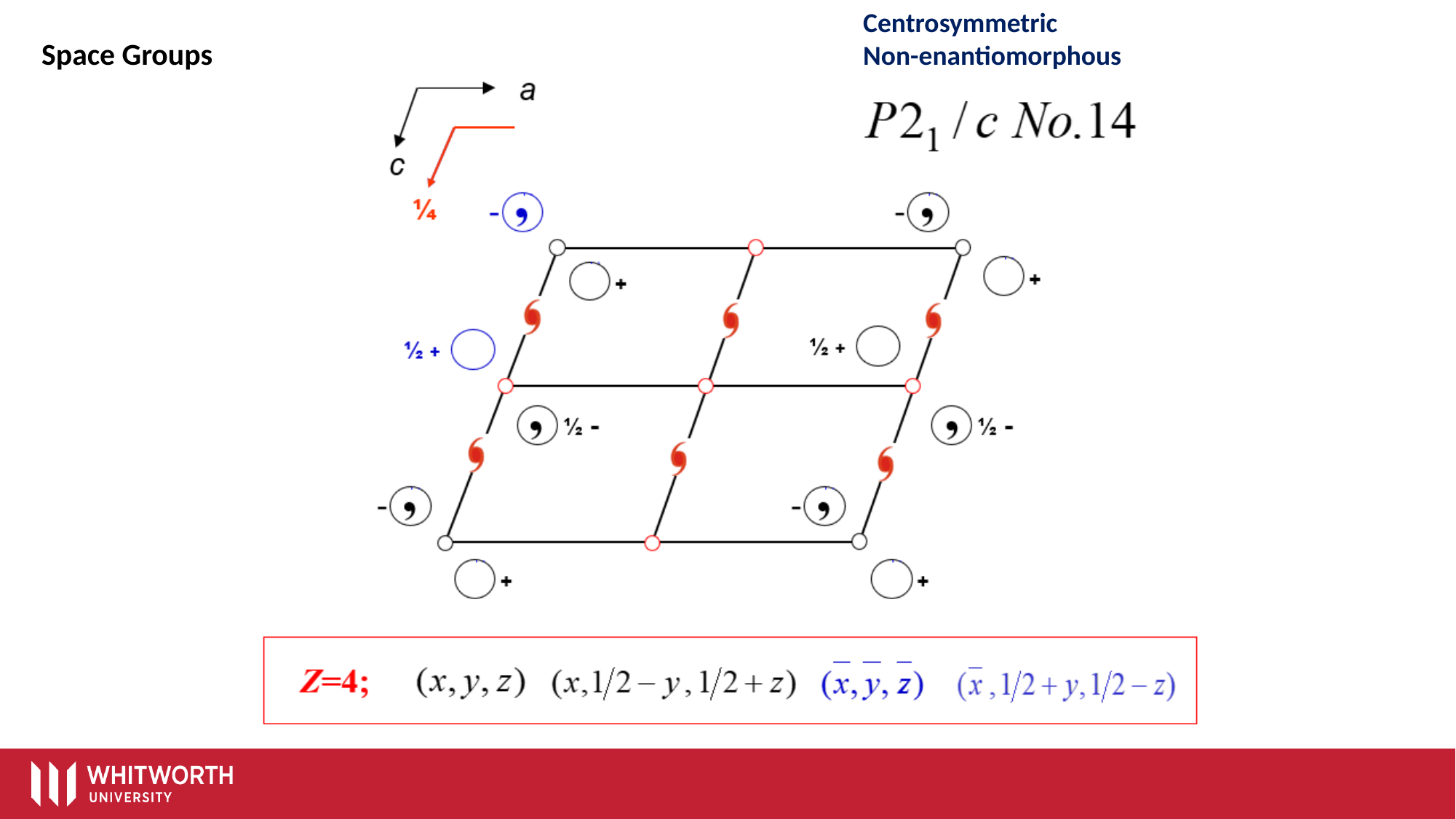

Centrosymmetric
Non-enantiomorphous
Space Groups

## Slide 38
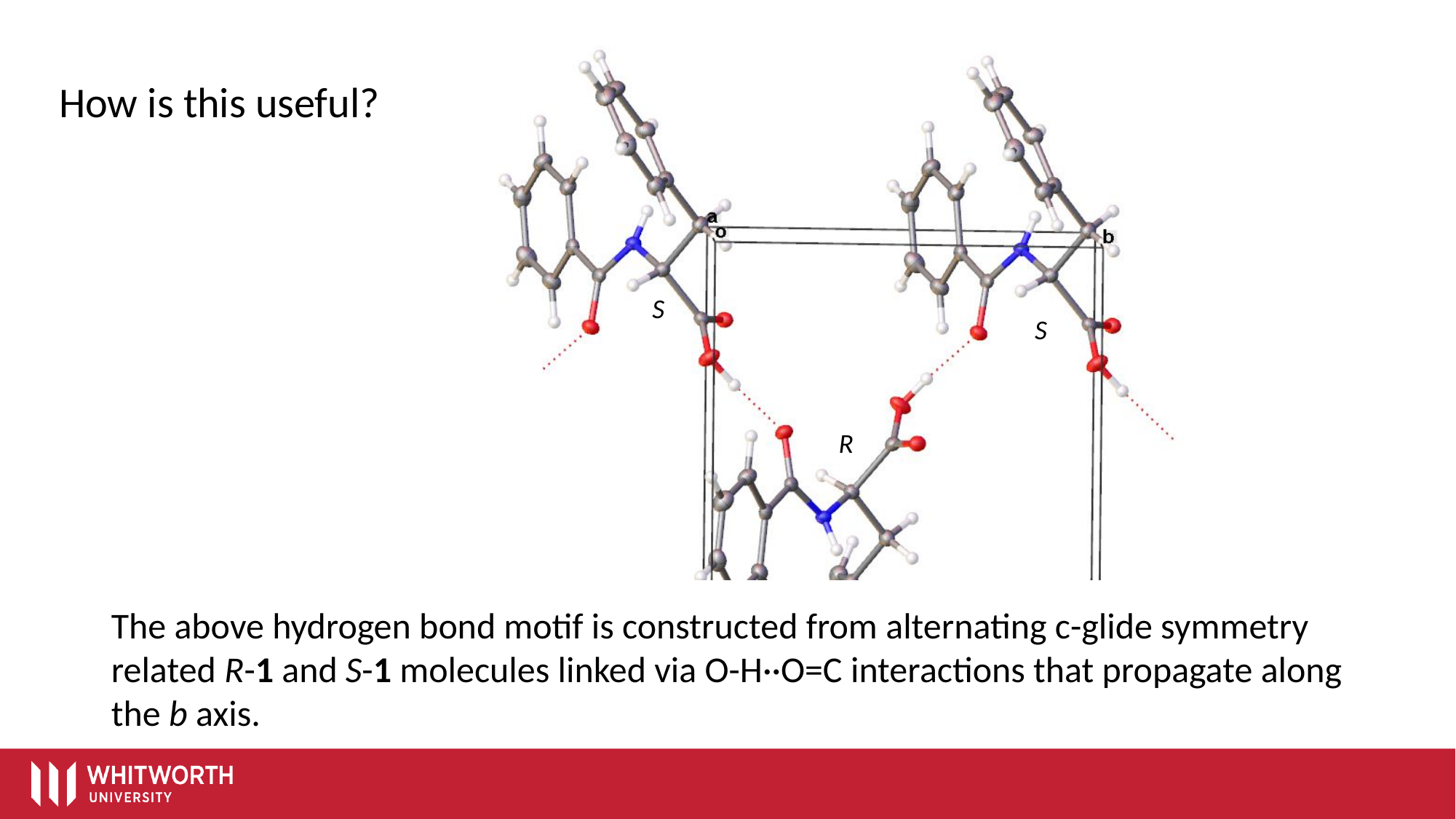

How is this useful?
S
S
R
The above hydrogen bond motif is constructed from alternating c-glide symmetry related R-1 and S-1 molecules linked via O-H··O=C interactions that propagate along the b axis.

## Slide 39
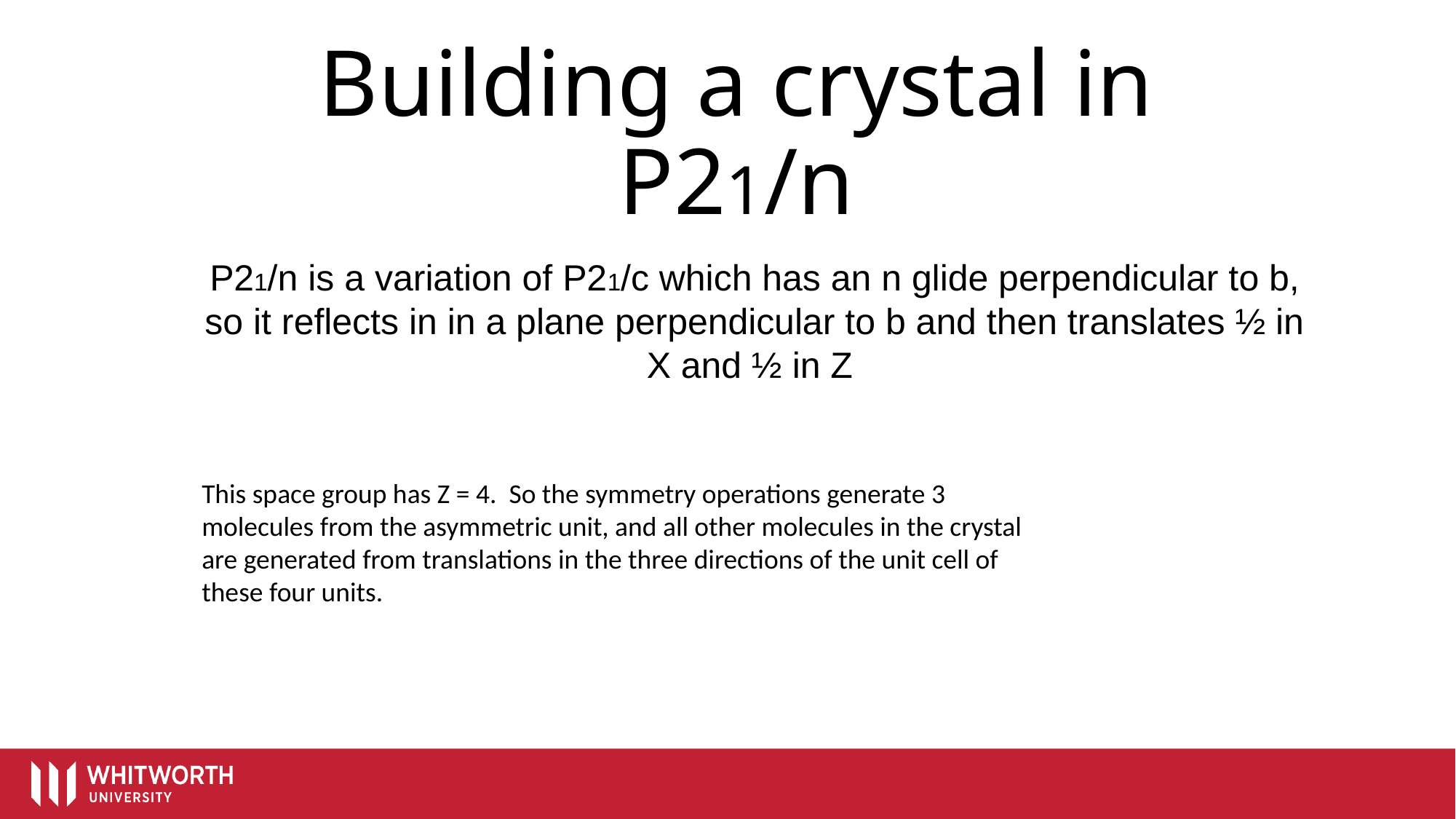

# Building a crystal in P21/n
P21/n is a variation of P21/c which has an n glide perpendicular to b, so it reflects in in a plane perpendicular to b and then translates ½ in X and ½ in Z
This space group has Z = 4. So the symmetry operations generate 3 molecules from the asymmetric unit, and all other molecules in the crystal are generated from translations in the three directions of the unit cell of these four units.
