# Supplementary material for: Crystallographic workshops – a primer and perspective from Whitworth University’s Summer Crystallography Institute
Source: Acta Crystallogr E Crystallogr Commun. 2026 Feb 3;82(Pt 3):313–9. doi: 10.1107/S2056989026000939 (PMC12961664; doi:10.1107/S2056989026000939)
Supplement: Supplementary file 1 [file e-82-00313-sup2.zip › Lectures/SCI_Diffraction.pptx]

## Slide 1
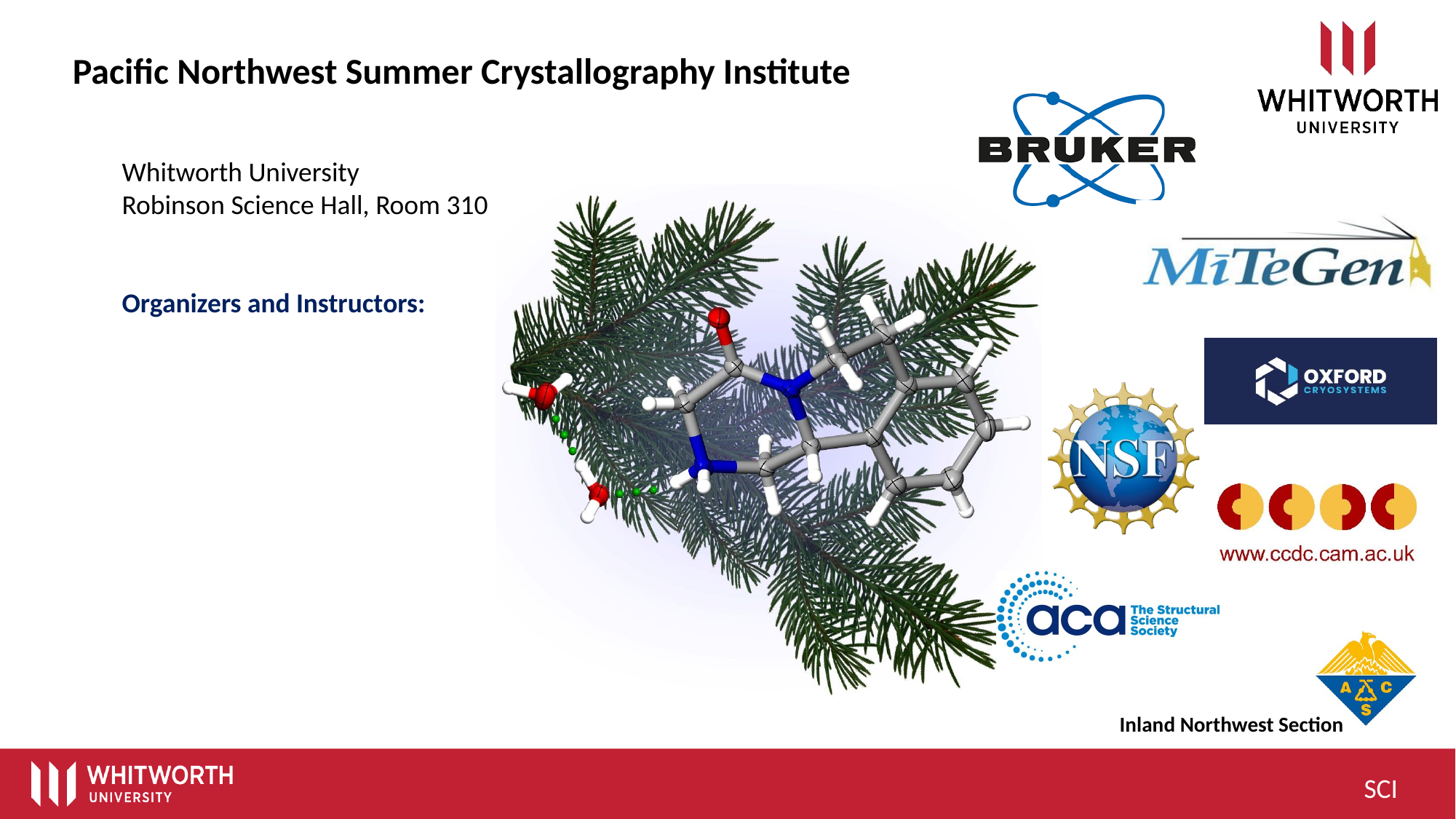

Pacific Northwest Summer Crystallography Institute
Whitworth University
Robinson Science Hall, Room 310
Organizers and Instructors:
Inland Northwest Section
SCI

## Slide 2
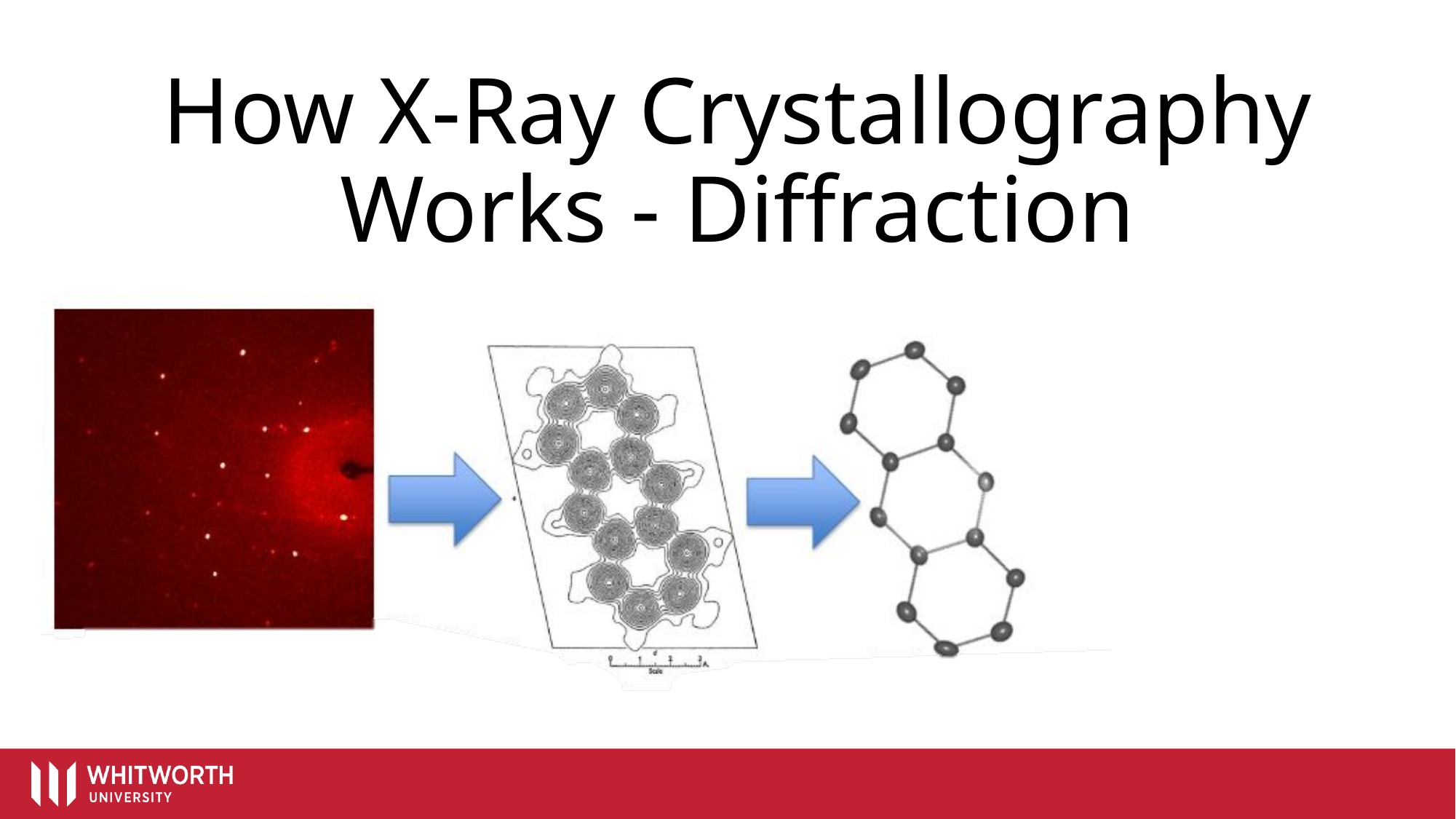

# How X-Ray Crystallography Works - Diffraction

## Slide 3
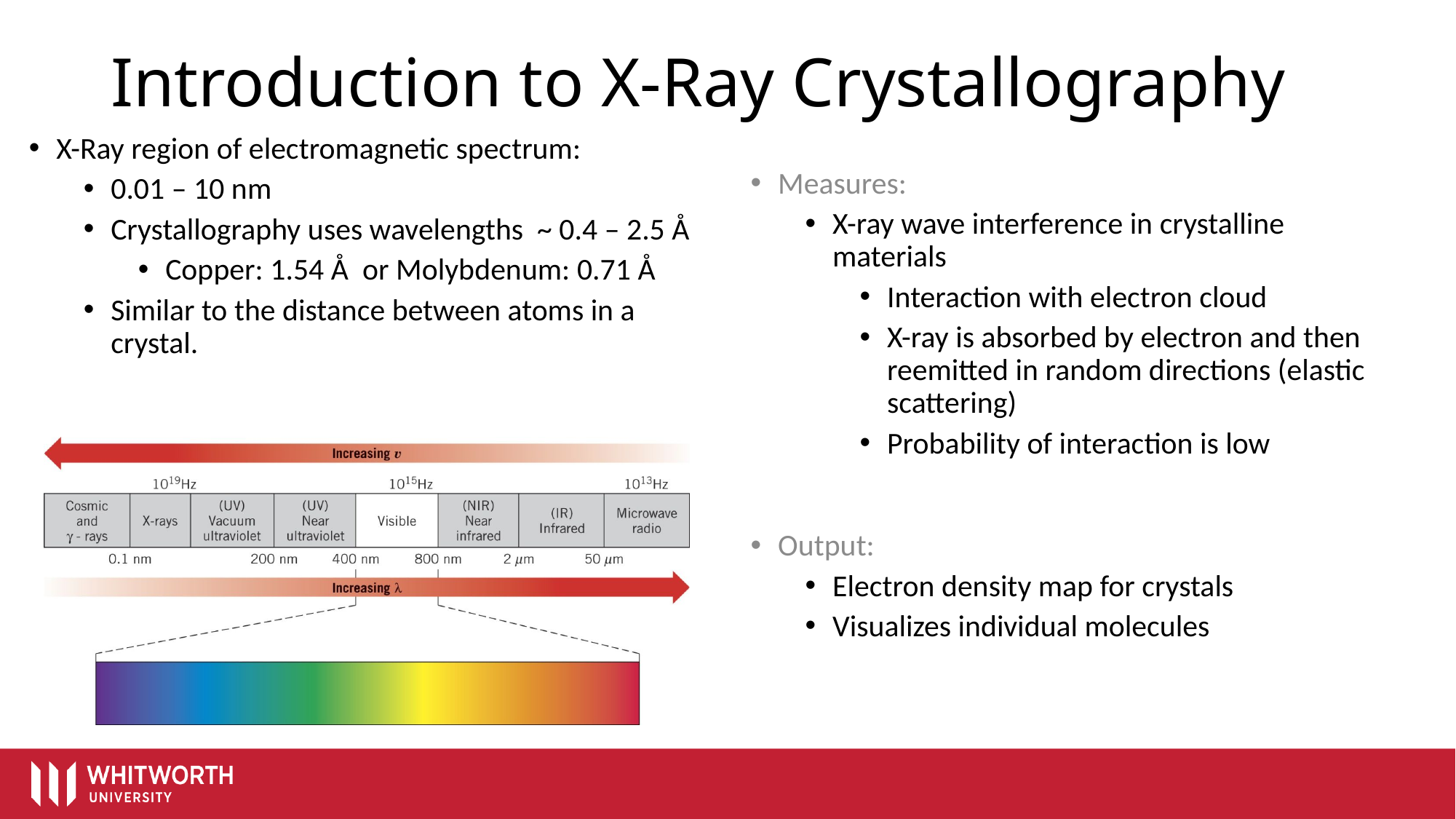

# Introduction to X-Ray Crystallography
X-Ray region of electromagnetic spectrum:
0.01 – 10 nm
Crystallography uses wavelengths ~ 0.4 – 2.5 Å
Copper: 1.54 Å or Molybdenum: 0.71 Å
Similar to the distance between atoms in a crystal.
Measures:
X-ray wave interference in crystalline materials
Interaction with electron cloud
X-ray is absorbed by electron and then reemitted in random directions (elastic scattering)
Probability of interaction is low
Output:
Electron density map for crystals
Visualizes individual molecules

## Slide 4
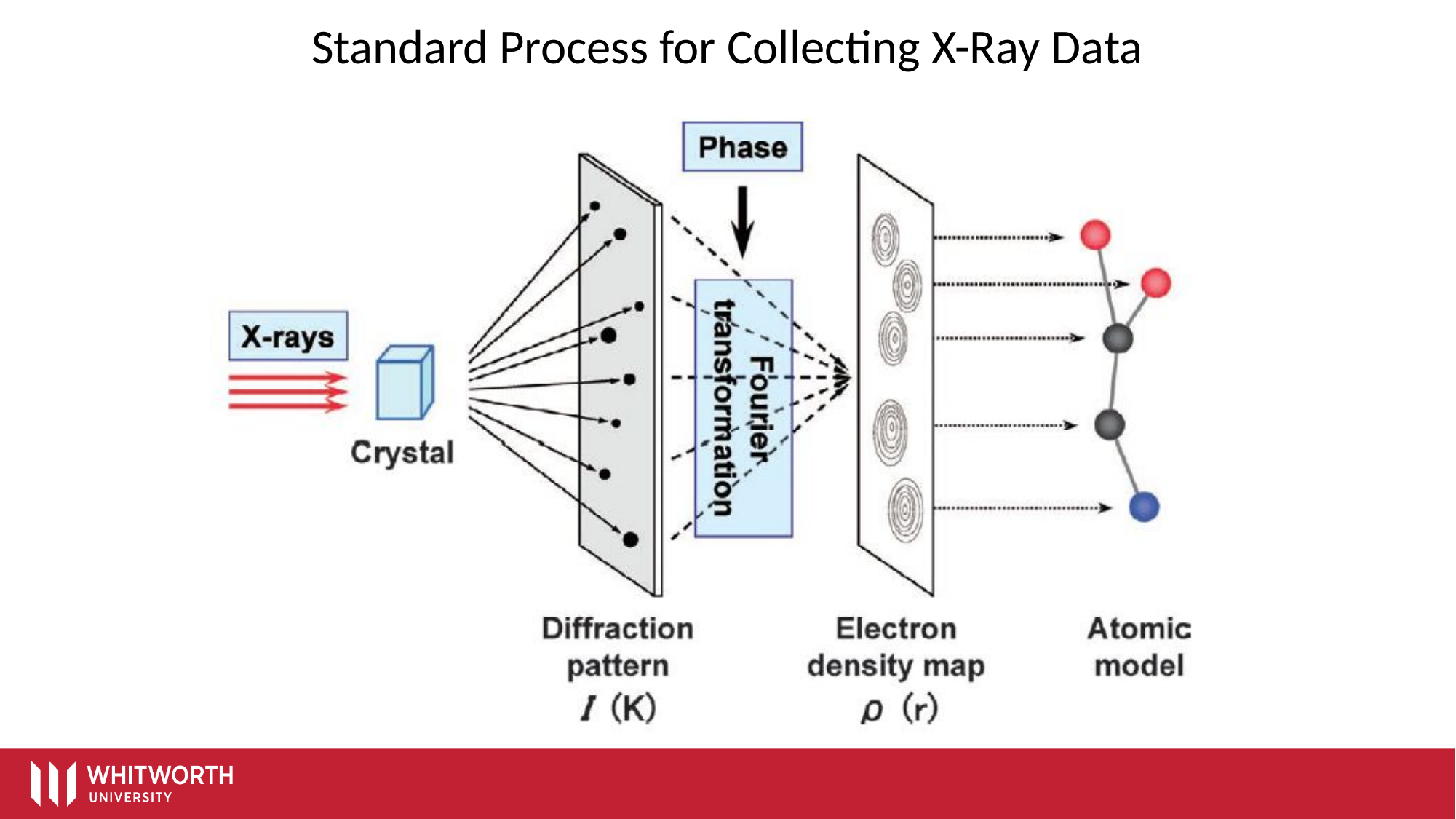

# Standard Process for Collecting X-Ray Data

## Slide 5
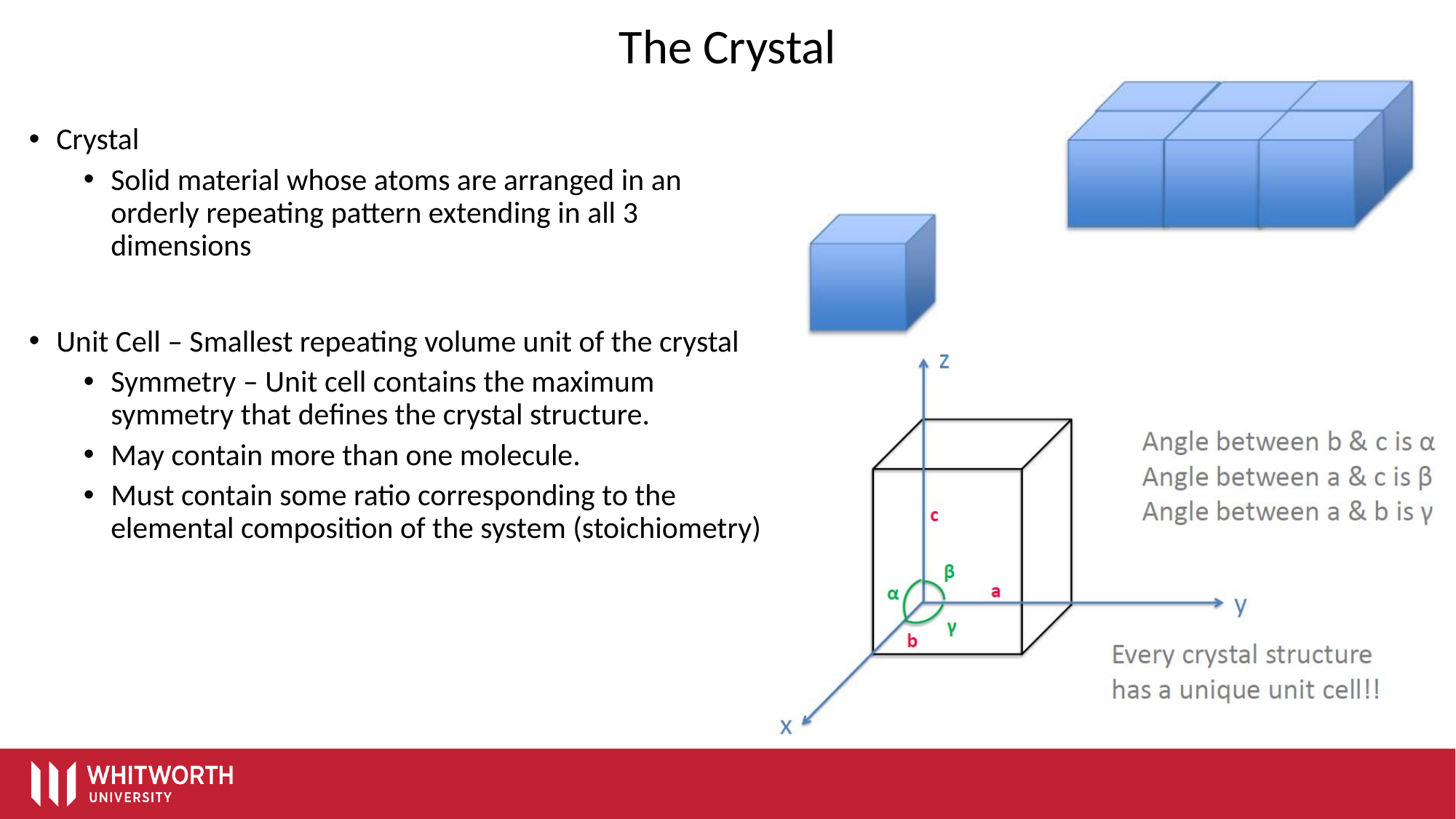

# The Crystal
Crystal
Solid material whose atoms are arranged in an orderly repeating pattern extending in all 3 dimensions
Unit Cell – Smallest repeating volume unit of the crystal
Symmetry – Unit cell contains the maximum symmetry that defines the crystal structure.
May contain more than one molecule.
Must contain some ratio corresponding to the elemental composition of the system (stoichiometry)

## Slide 6
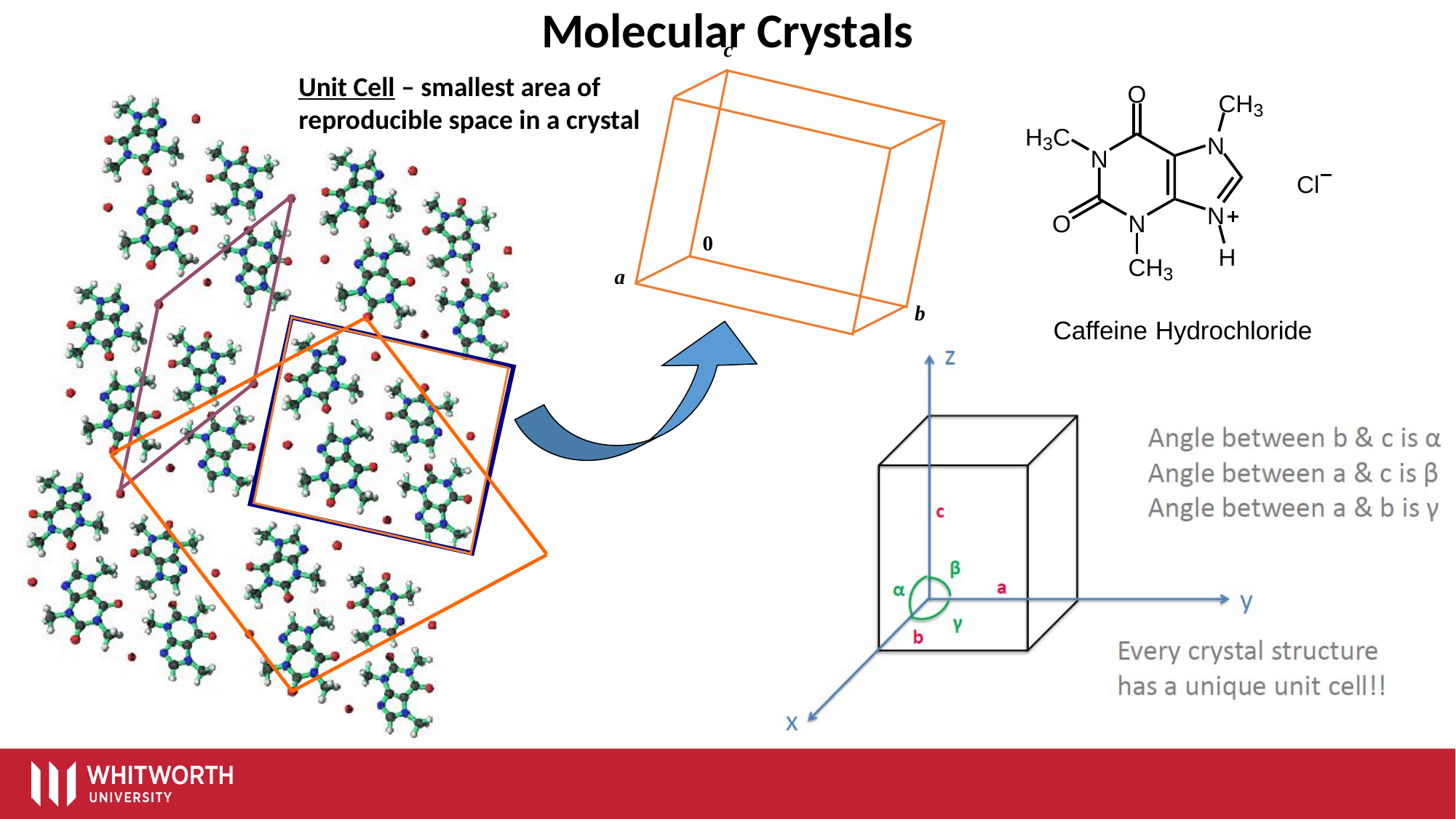

# Molecular Crystals
c
0
a
b
Unit Cell – smallest area of reproducible space in a crystal

## Slide 7
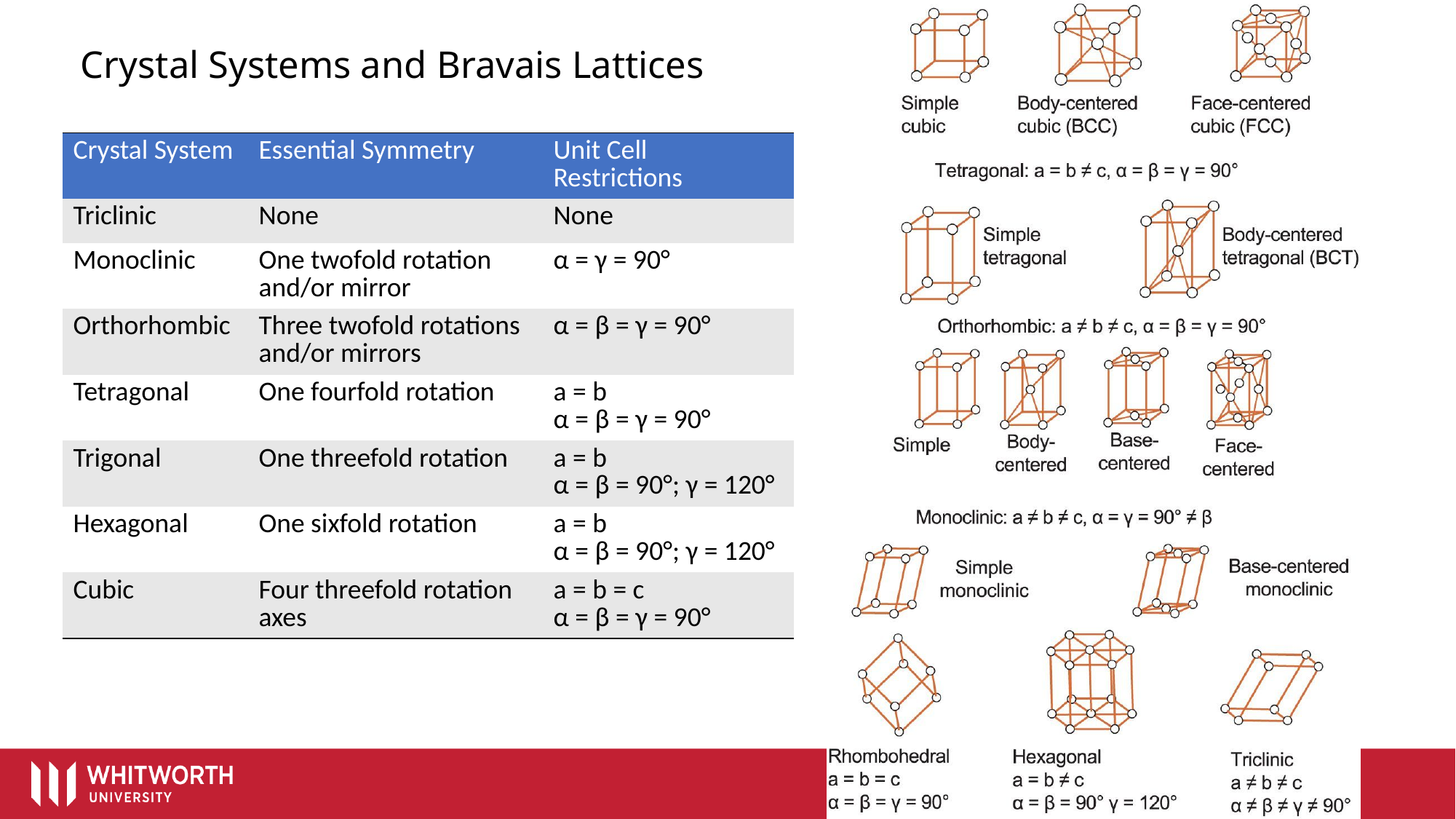

Crystal Systems and Bravais Lattices
| Crystal System | Essential Symmetry | Unit Cell Restrictions |
| --- | --- | --- |
| Triclinic | None | None |
| Monoclinic | One twofold rotation and/or mirror | α = γ = 90° |
| Orthorhombic | Three twofold rotations and/or mirrors | α = β = γ = 90° |
| Tetragonal | One fourfold rotation | a = b α = β = γ = 90° |
| Trigonal | One threefold rotation | a = b α = β = 90°; γ = 120° |
| Hexagonal | One sixfold rotation | a = b α = β = 90°; γ = 120° |
| Cubic | Four threefold rotation axes | a = b = c α = β = γ = 90° |

## Slide 8
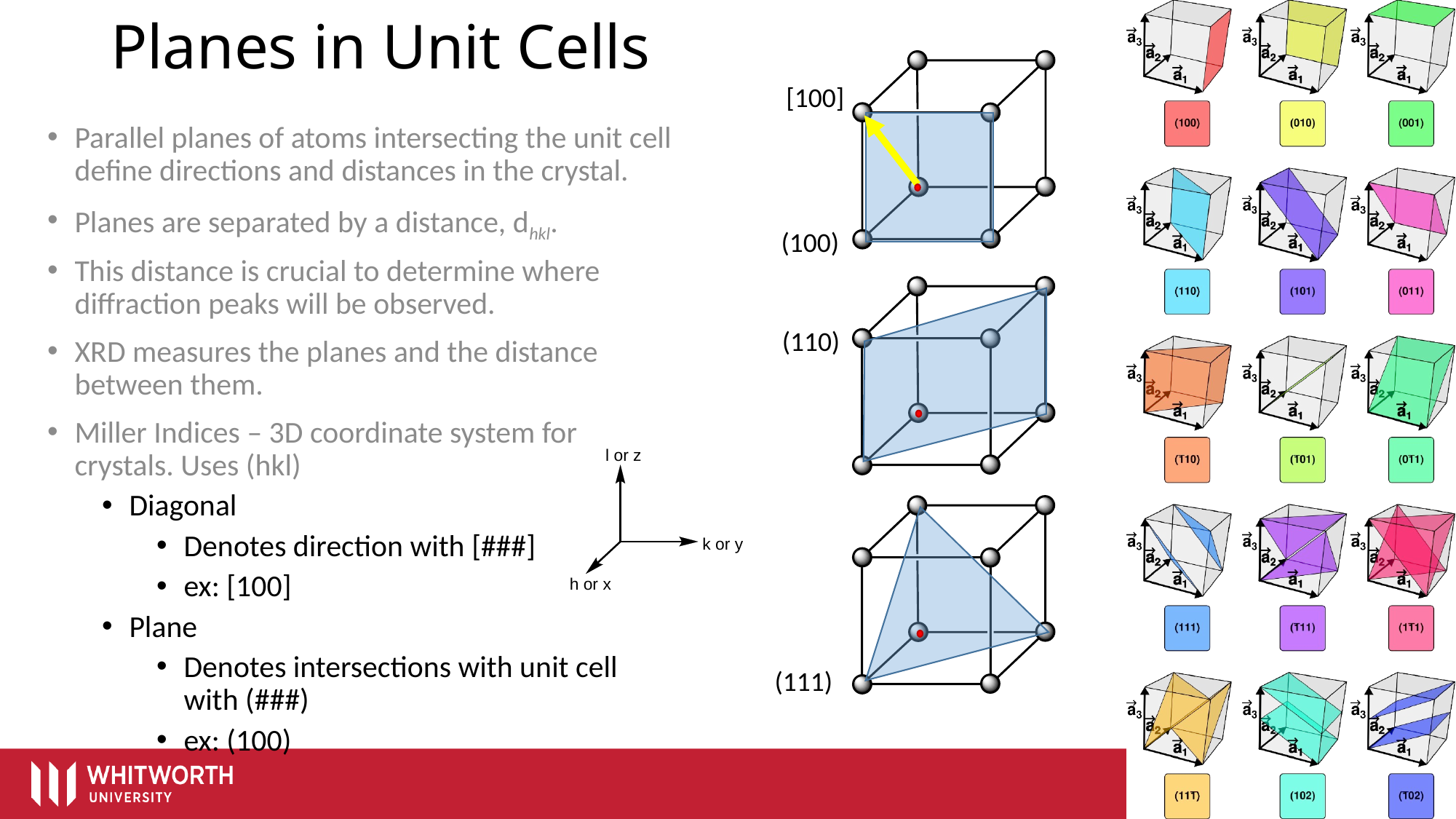

# Planes in Unit Cells
[100]
Parallel planes of atoms intersecting the unit cell define directions and distances in the crystal.
Planes are separated by a distance, dhkl.
This distance is crucial to determine where diffraction peaks will be observed.
XRD measures the planes and the distance between them.
Miller Indices – 3D coordinate system for crystals. Uses (hkl)
Diagonal
Denotes direction with [###]
ex: [100]
Plane
Denotes intersections with unit cell with (###)
ex: (100)
(100)
(110)
(111)

## Slide 9
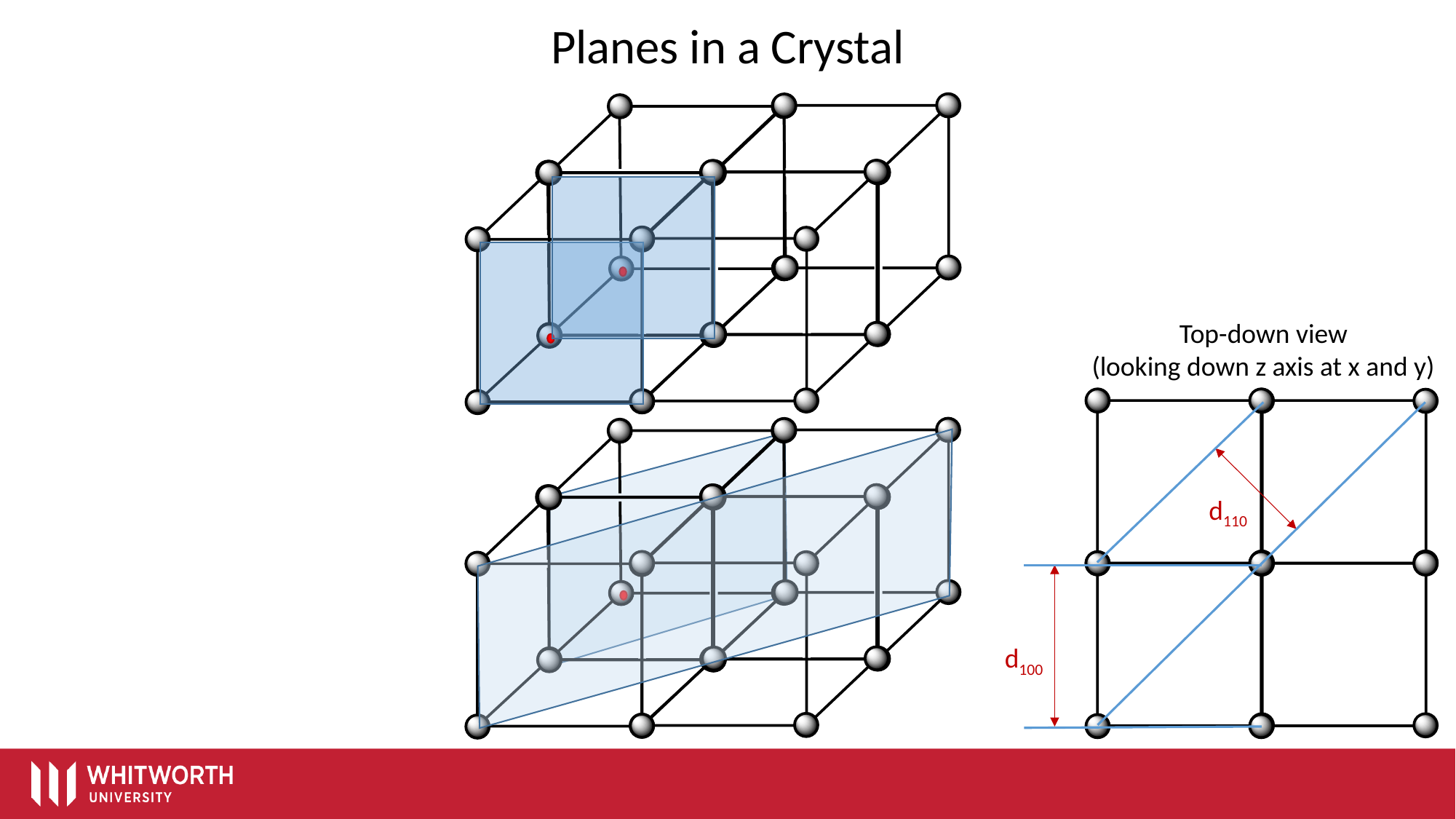

# Planes in a Crystal
Top-down view
(looking down z axis at x and y)
d110
d100

## Slide 10
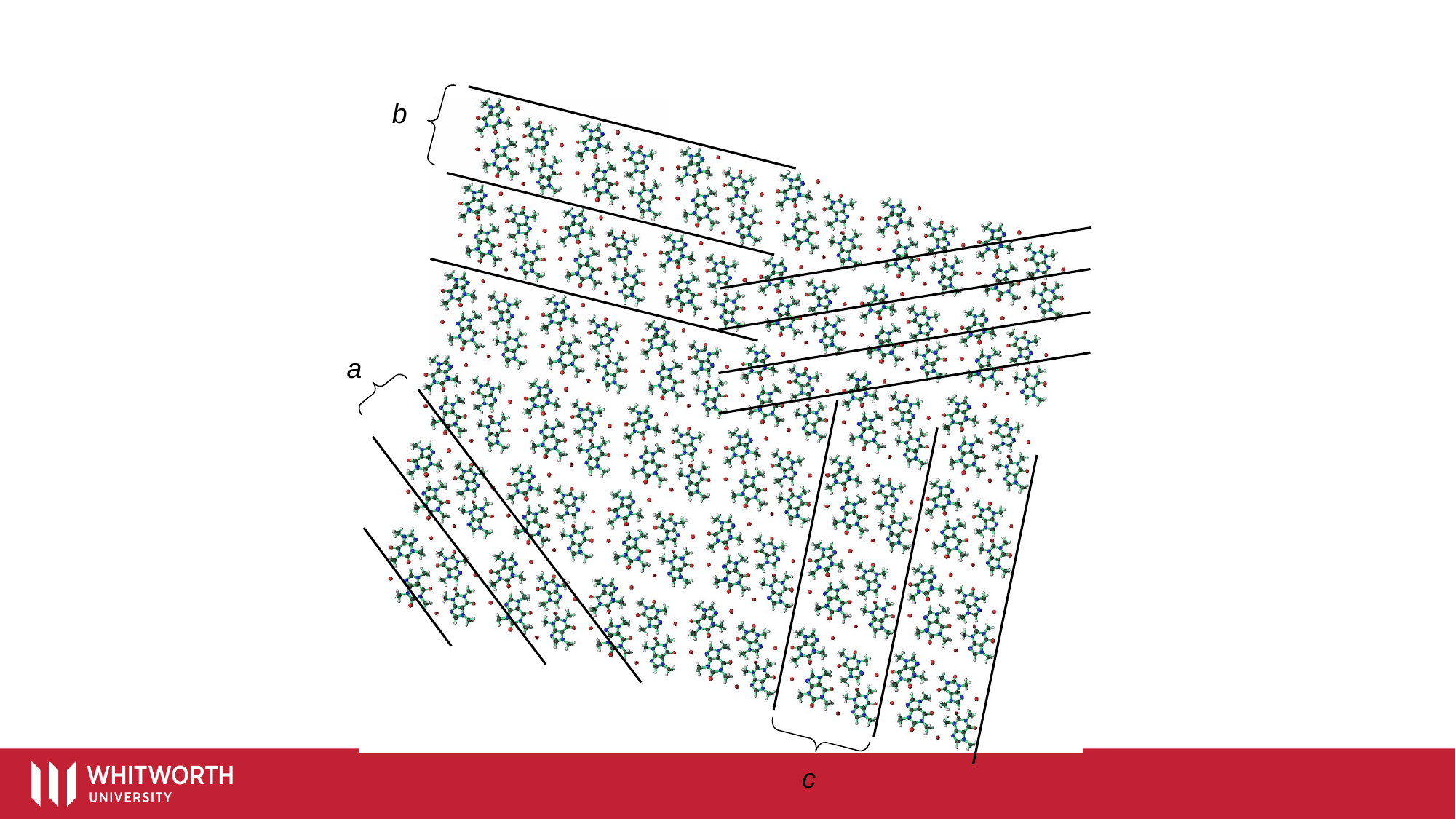

b
a
c

## Slide 11
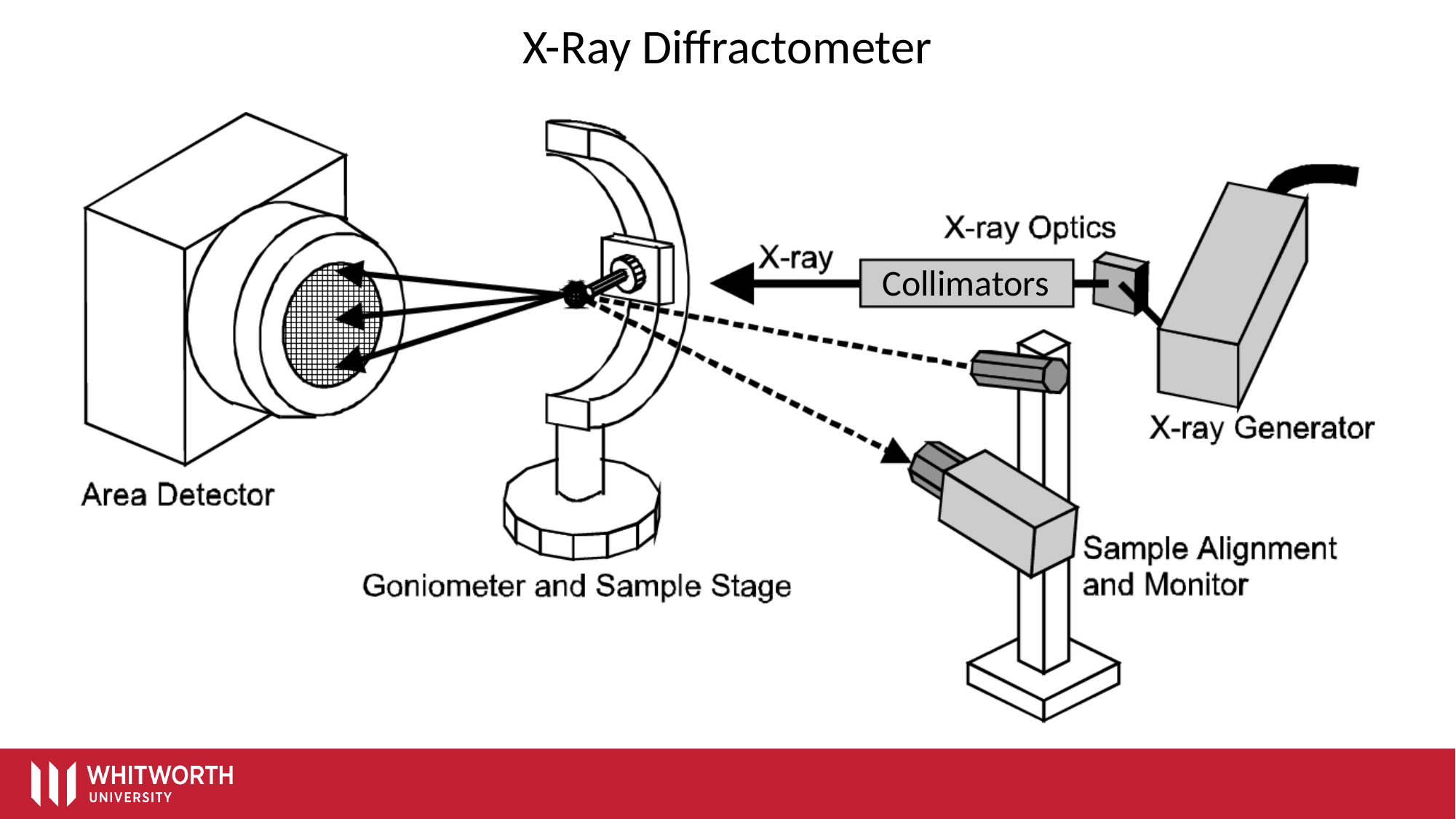

# X-Ray Diffractometer
Collimators

## Slide 12
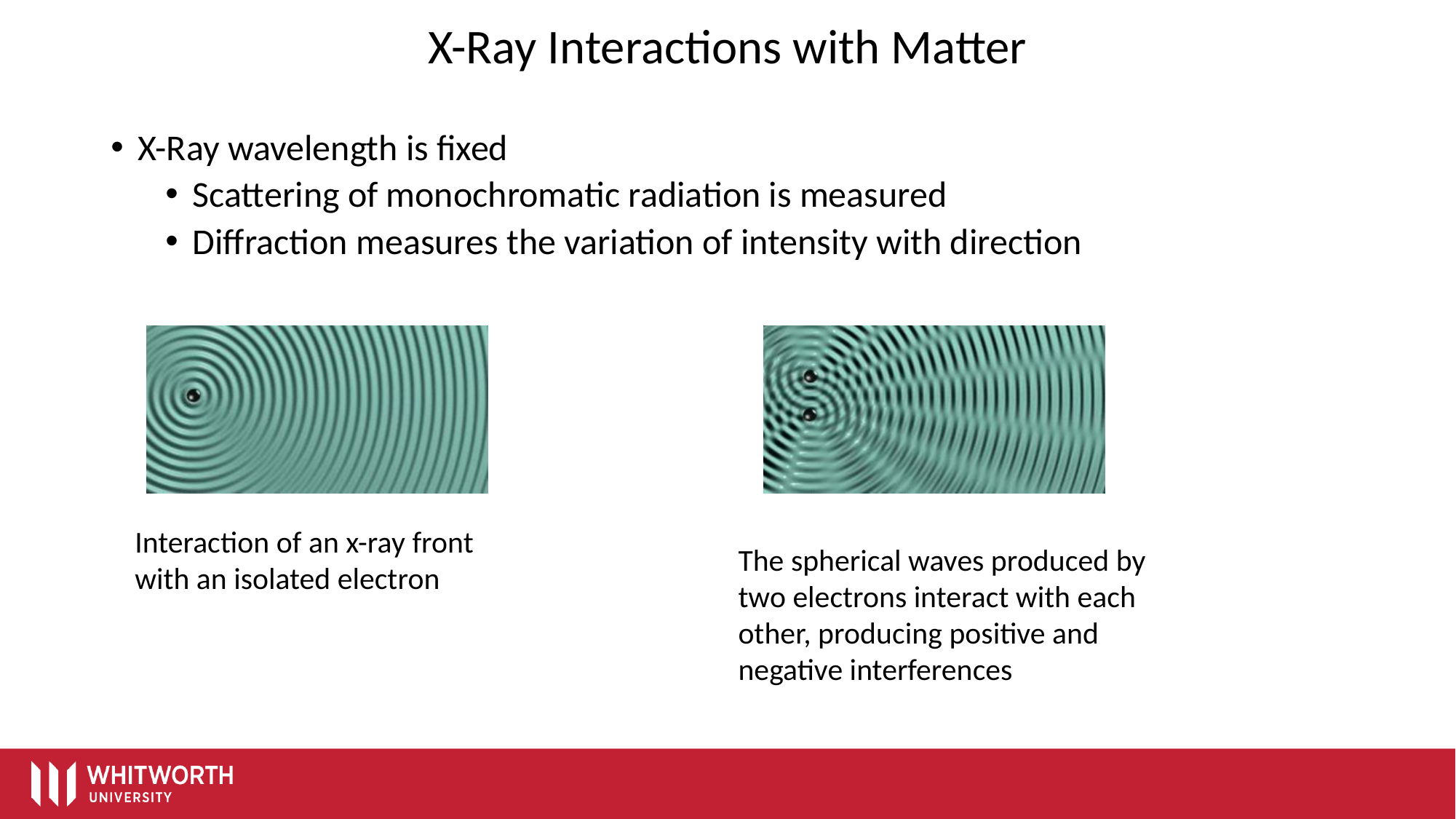

# X-Ray Interactions with Matter
X-Ray wavelength is fixed
Scattering of monochromatic radiation is measured
Diffraction measures the variation of intensity with direction
Interaction of an x-ray front with an isolated electron
The spherical waves produced by two electrons interact with each other, producing positive and negative interferences

## Slide 13
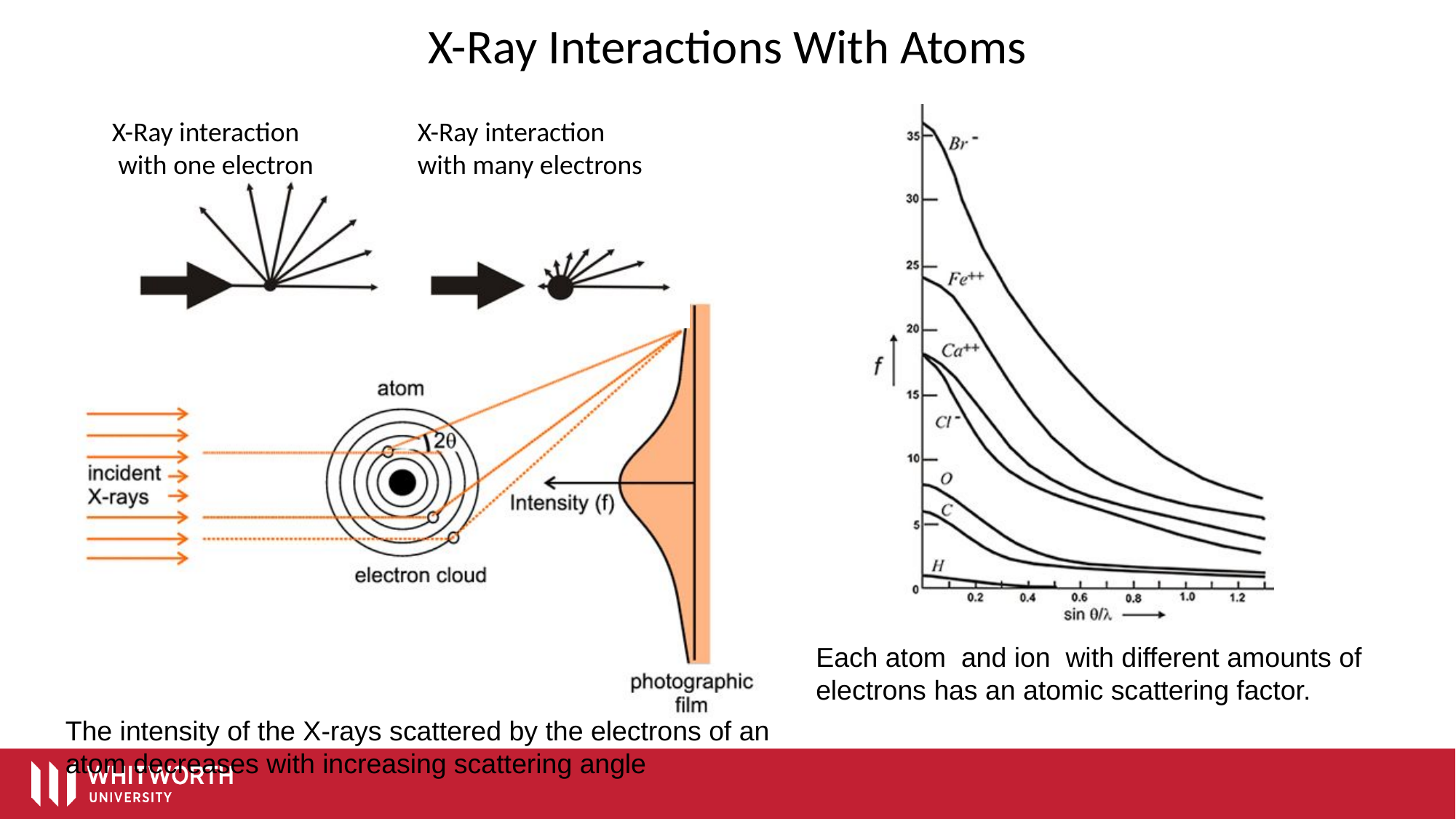

# X-Ray Interactions With Atoms
X-Ray interaction
with many electrons
X-Ray interaction
 with one electron
Each atom and ion with different amounts of electrons has an atomic scattering factor.
The intensity of the X-rays scattered by the electrons of an atom decreases with increasing scattering angle

## Slide 14
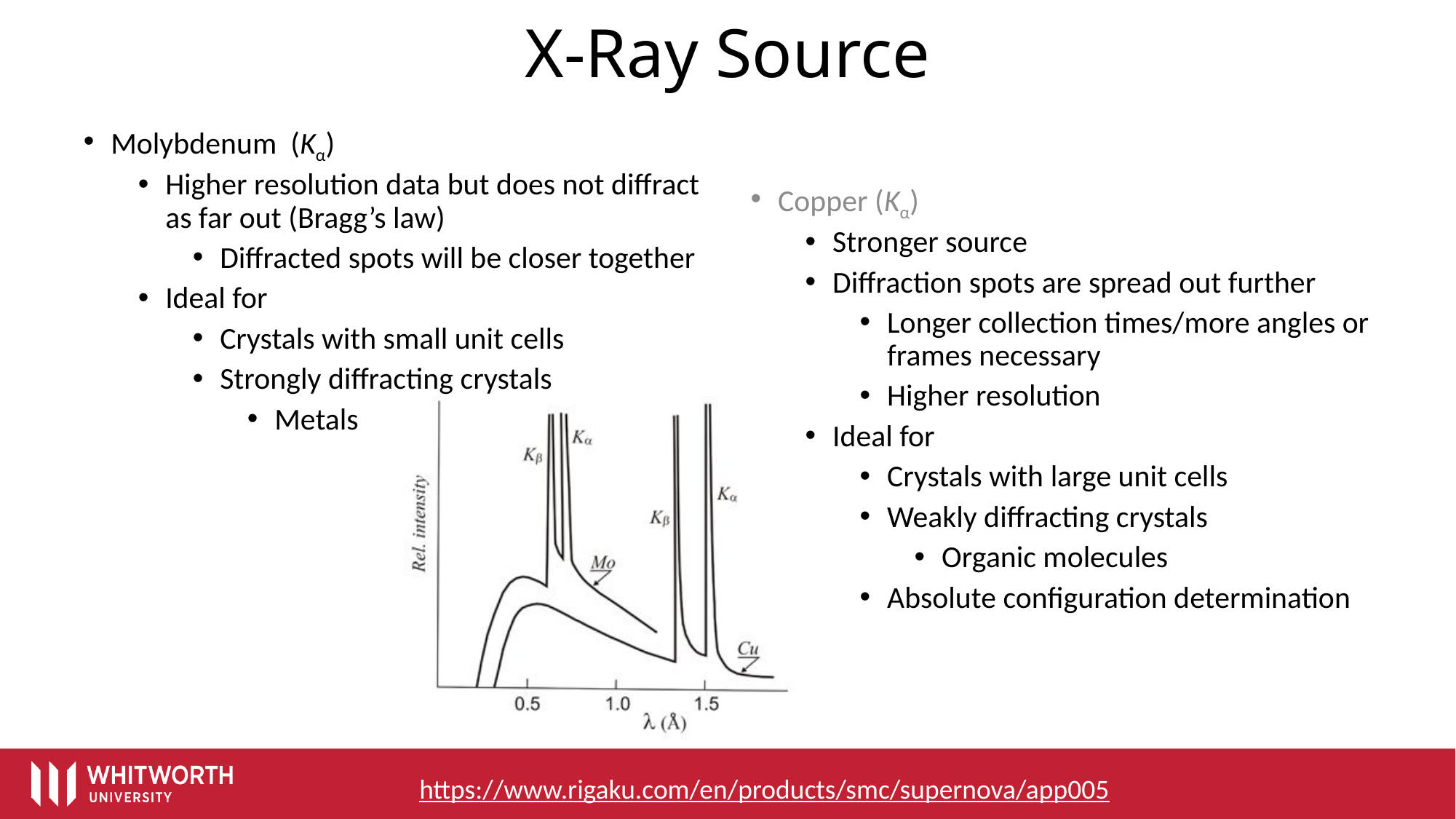

# X-Ray Source
Molybdenum (Kα)
Higher resolution data but does not diffract as far out (Bragg’s law)
Diffracted spots will be closer together
Ideal for
Crystals with small unit cells
Strongly diffracting crystals
Metals
Copper (Kα)
Stronger source
Diffraction spots are spread out further
Longer collection times/more angles or frames necessary
Higher resolution
Ideal for
Crystals with large unit cells
Weakly diffracting crystals
Organic molecules
Absolute configuration determination
https://www.rigaku.com/en/products/smc/supernova/app005

## Slide 15
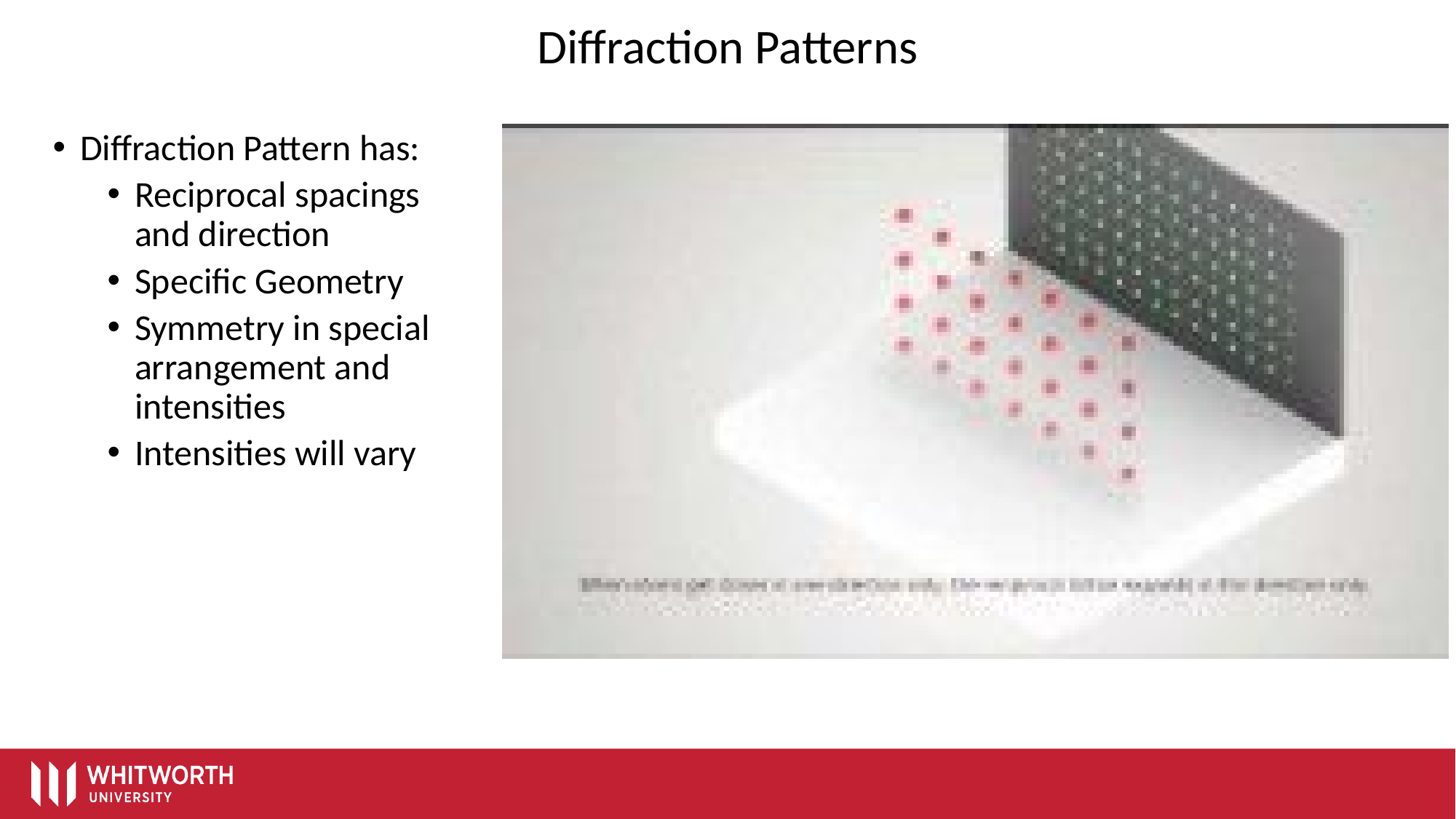

# Diffraction Patterns
Diffraction Pattern has:
Reciprocal spacings and direction
Specific Geometry
Symmetry in special arrangement and intensities
Intensities will vary

## Slide 16
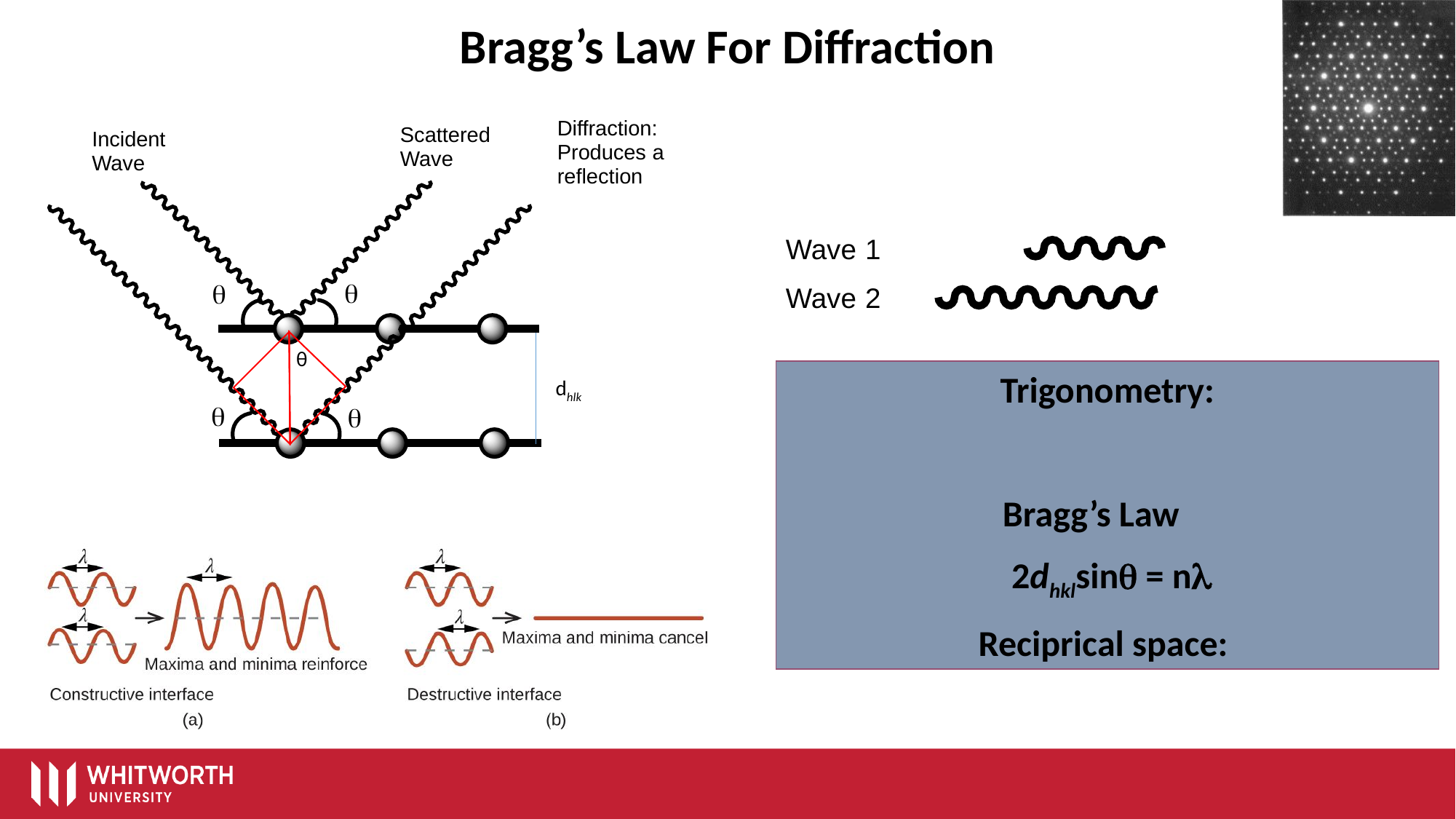

# Bragg’s Law For Diffraction
θ
dhlk

## Slide 17
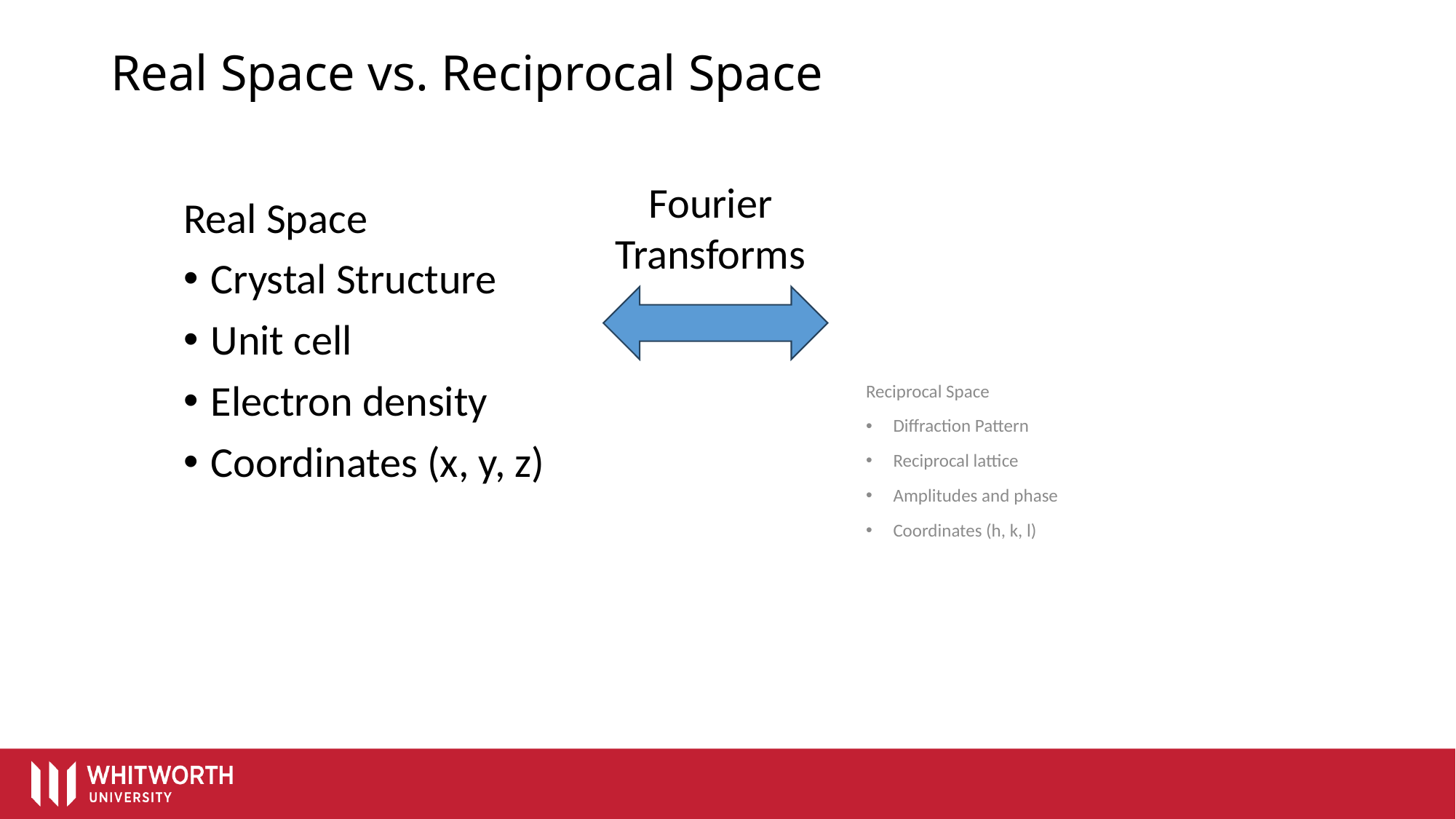

# Real Space vs. Reciprocal Space
Fourier
Transforms
Real Space
Crystal Structure
Unit cell
Electron density
Coordinates (x, y, z)
Reciprocal Space
Diffraction Pattern
Reciprocal lattice
Amplitudes and phase
Coordinates (h, k, l)

## Slide 18
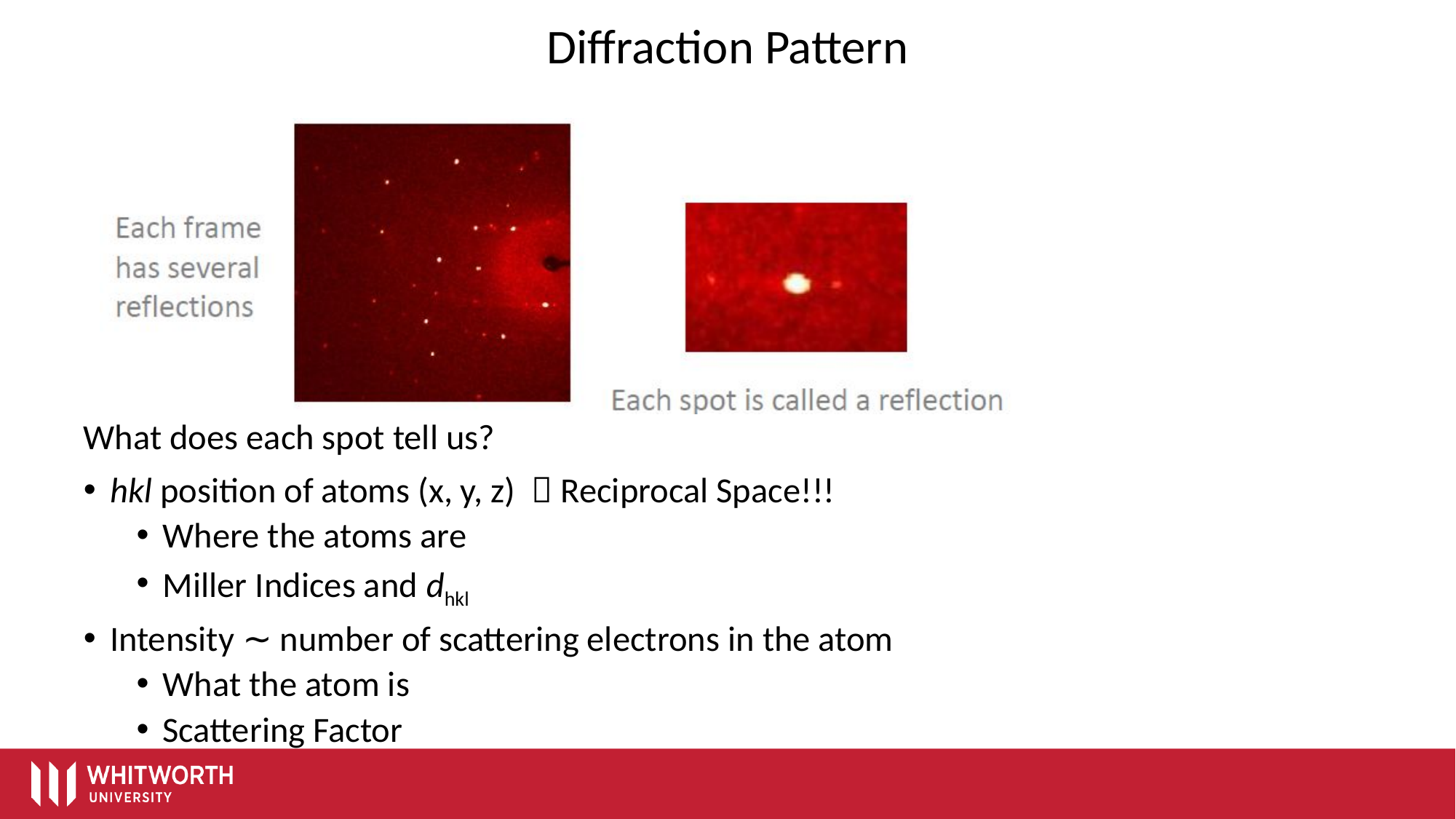

# Diffraction Pattern
What does each spot tell us?
hkl position of atoms (x, y, z)  Reciprocal Space!!!
Where the atoms are
Miller Indices and dhkl
Intensity ∼ number of scattering electrons in the atom
What the atom is
Scattering Factor

## Slide 19
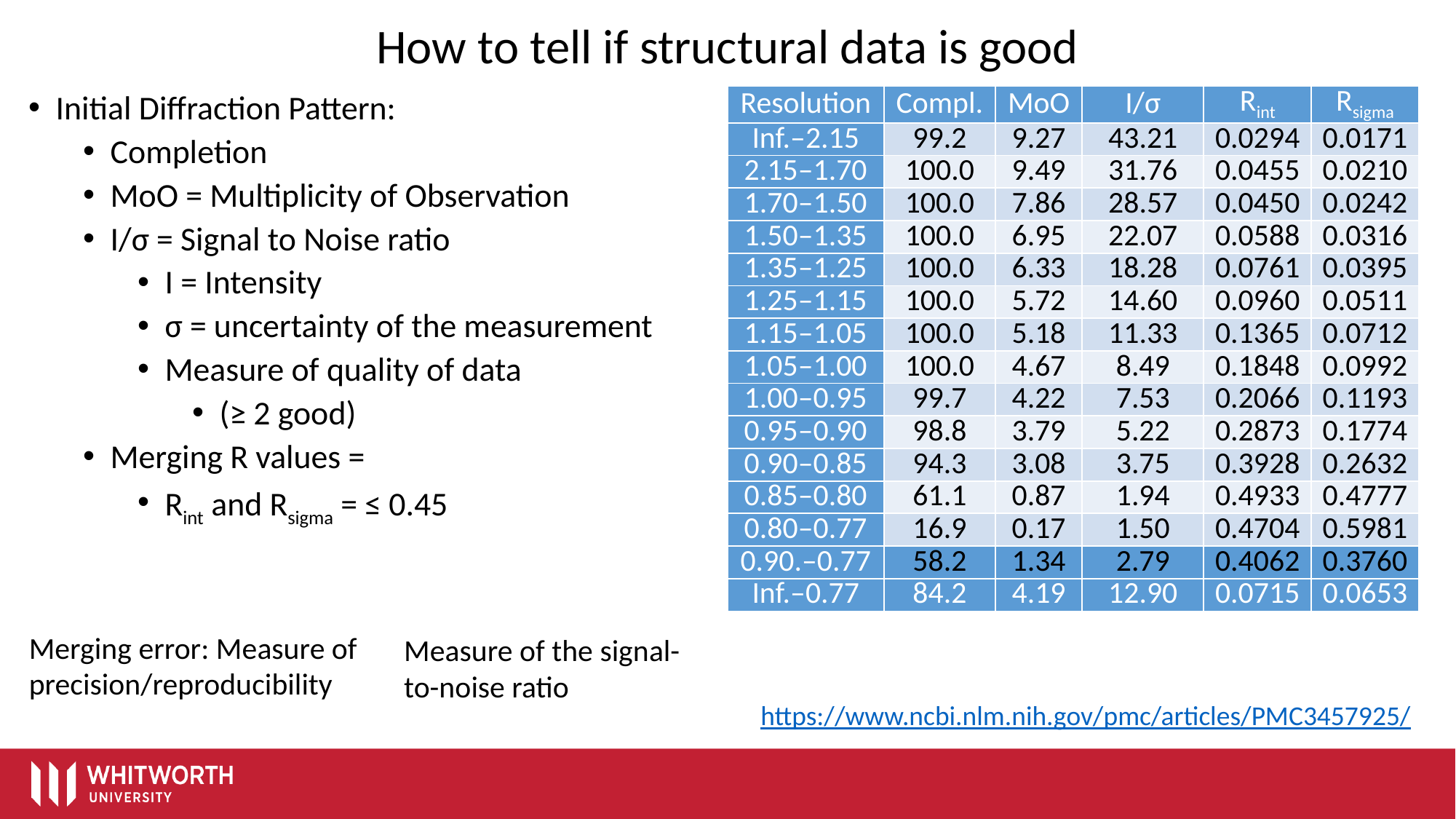

# How to tell if structural data is good
Initial Diffraction Pattern:
Completion
MoO = Multiplicity of Observation
I/σ = Signal to Noise ratio
I = Intensity
σ = uncertainty of the measurement
Measure of quality of data
(≥ 2 good)
Merging R values =
Rint and Rsigma = ≤ 0.45
| Resolution | Compl. | MoO | I/σ | Rint | Rsigma |
| --- | --- | --- | --- | --- | --- |
| Inf.–2.15 | 99.2 | 9.27 | 43.21 | 0.0294 | 0.0171 |
| 2.15–1.70 | 100.0 | 9.49 | 31.76 | 0.0455 | 0.0210 |
| 1.70–1.50 | 100.0 | 7.86 | 28.57 | 0.0450 | 0.0242 |
| 1.50–1.35 | 100.0 | 6.95 | 22.07 | 0.0588 | 0.0316 |
| 1.35–1.25 | 100.0 | 6.33 | 18.28 | 0.0761 | 0.0395 |
| 1.25–1.15 | 100.0 | 5.72 | 14.60 | 0.0960 | 0.0511 |
| 1.15–1.05 | 100.0 | 5.18 | 11.33 | 0.1365 | 0.0712 |
| 1.05–1.00 | 100.0 | 4.67 | 8.49 | 0.1848 | 0.0992 |
| 1.00–0.95 | 99.7 | 4.22 | 7.53 | 0.2066 | 0.1193 |
| 0.95–0.90 | 98.8 | 3.79 | 5.22 | 0.2873 | 0.1774 |
| 0.90–0.85 | 94.3 | 3.08 | 3.75 | 0.3928 | 0.2632 |
| 0.85–0.80 | 61.1 | 0.87 | 1.94 | 0.4933 | 0.4777 |
| 0.80–0.77 | 16.9 | 0.17 | 1.50 | 0.4704 | 0.5981 |
| 0.90.–0.77 | 58.2 | 1.34 | 2.79 | 0.4062 | 0.3760 |
| Inf.–0.77 | 84.2 | 4.19 | 12.90 | 0.0715 | 0.0653 |
https://www.ncbi.nlm.nih.gov/pmc/articles/PMC3457925/

## Slide 20
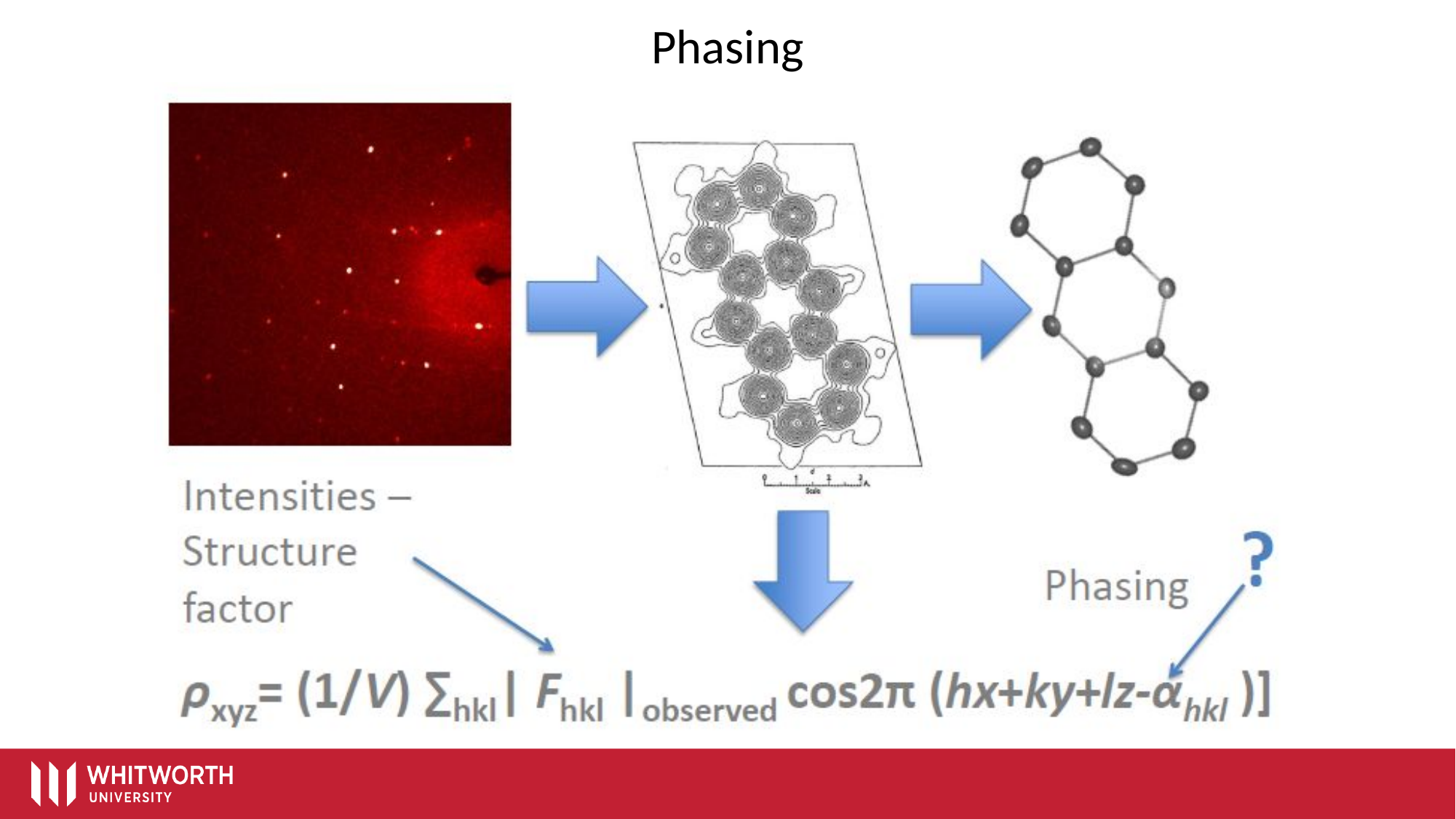

# Phasing

## Slide 21
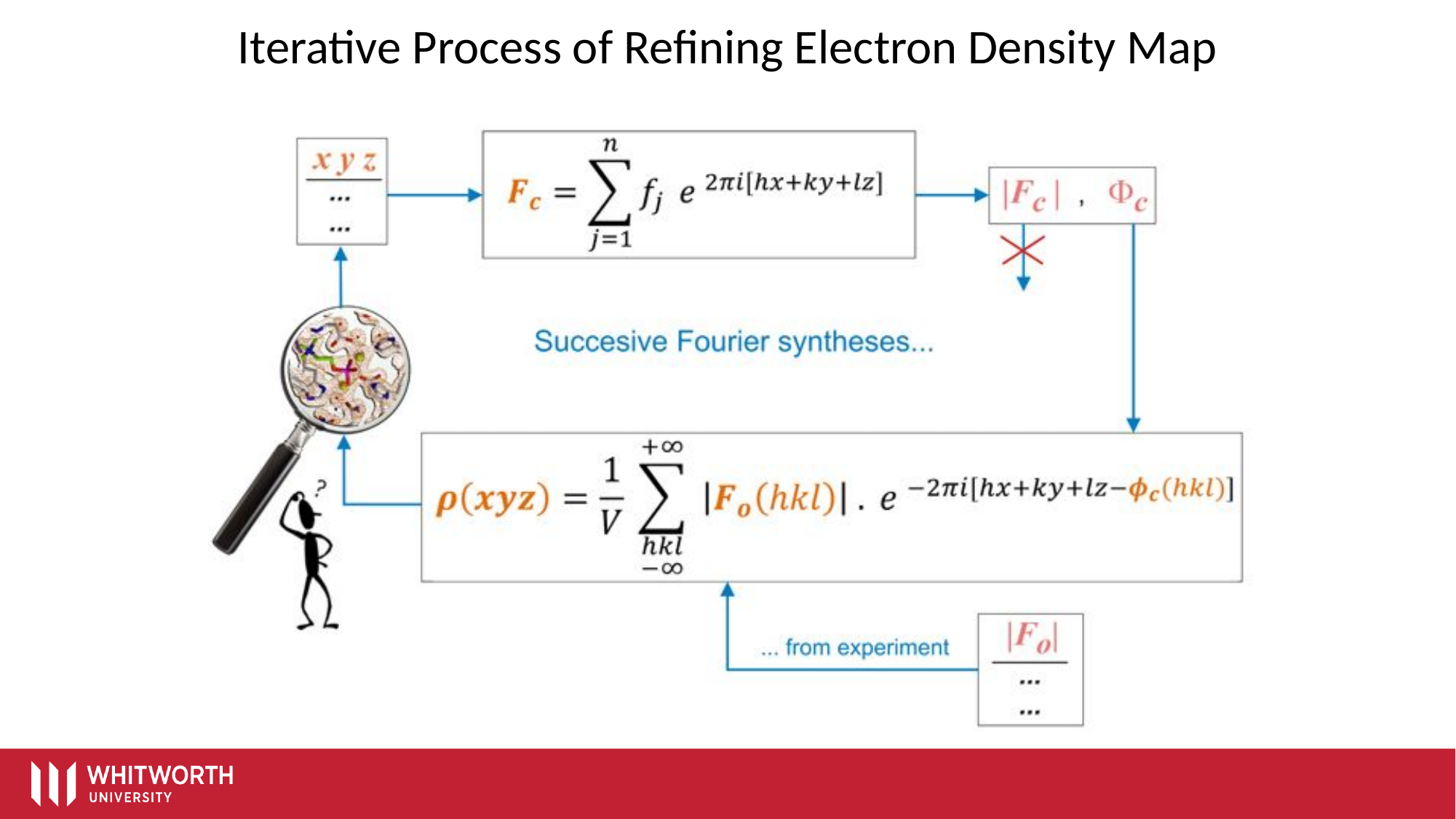

# Iterative Process of Refining Electron Density Map

## Slide 22
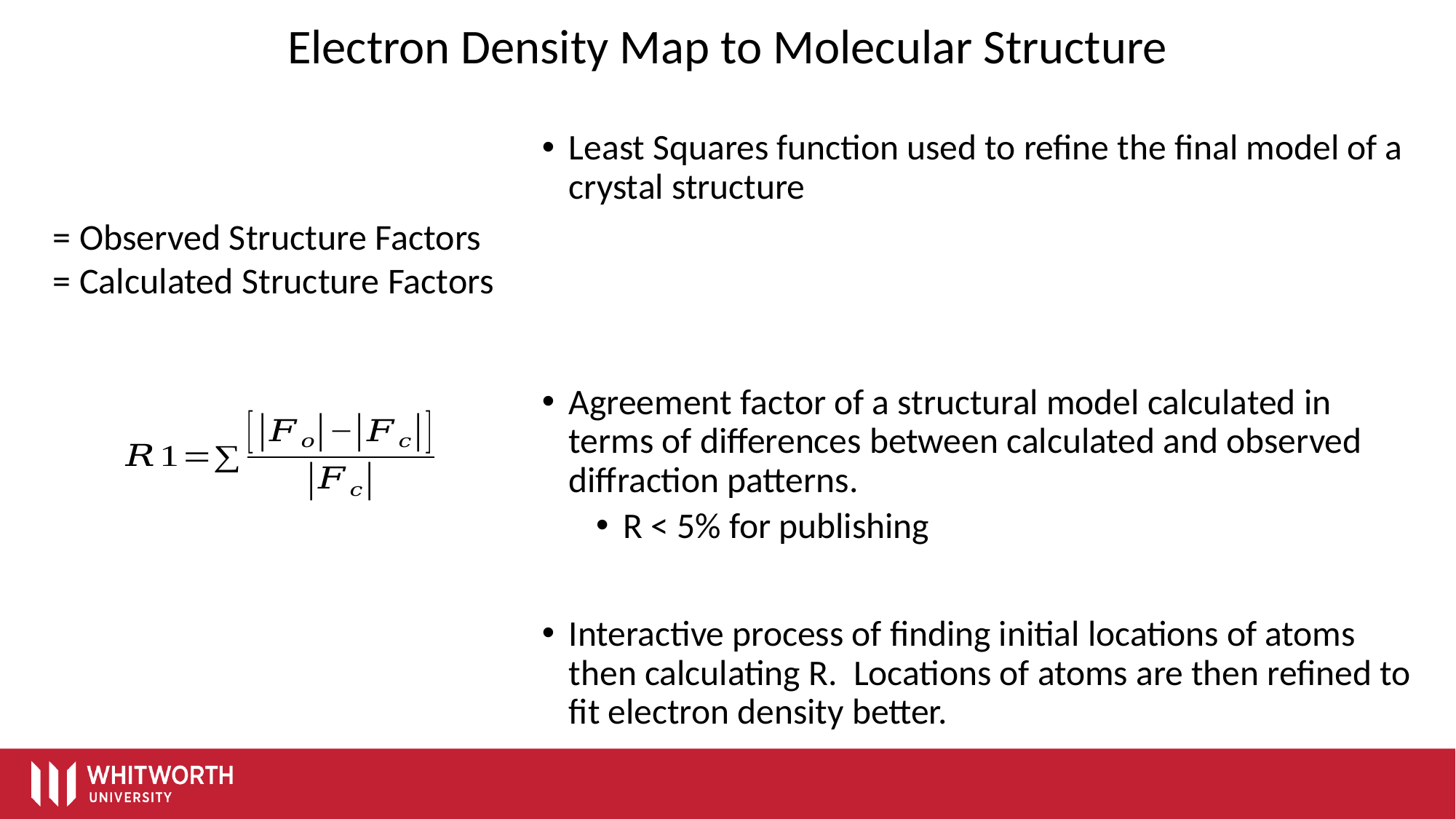

# Electron Density Map to Molecular Structure
Least Squares function used to refine the final model of a crystal structure
Agreement factor of a structural model calculated in terms of differences between calculated and observed diffraction patterns.
R < 5% for publishing
Interactive process of finding initial locations of atoms then calculating R. Locations of atoms are then refined to fit electron density better.

## Slide 23
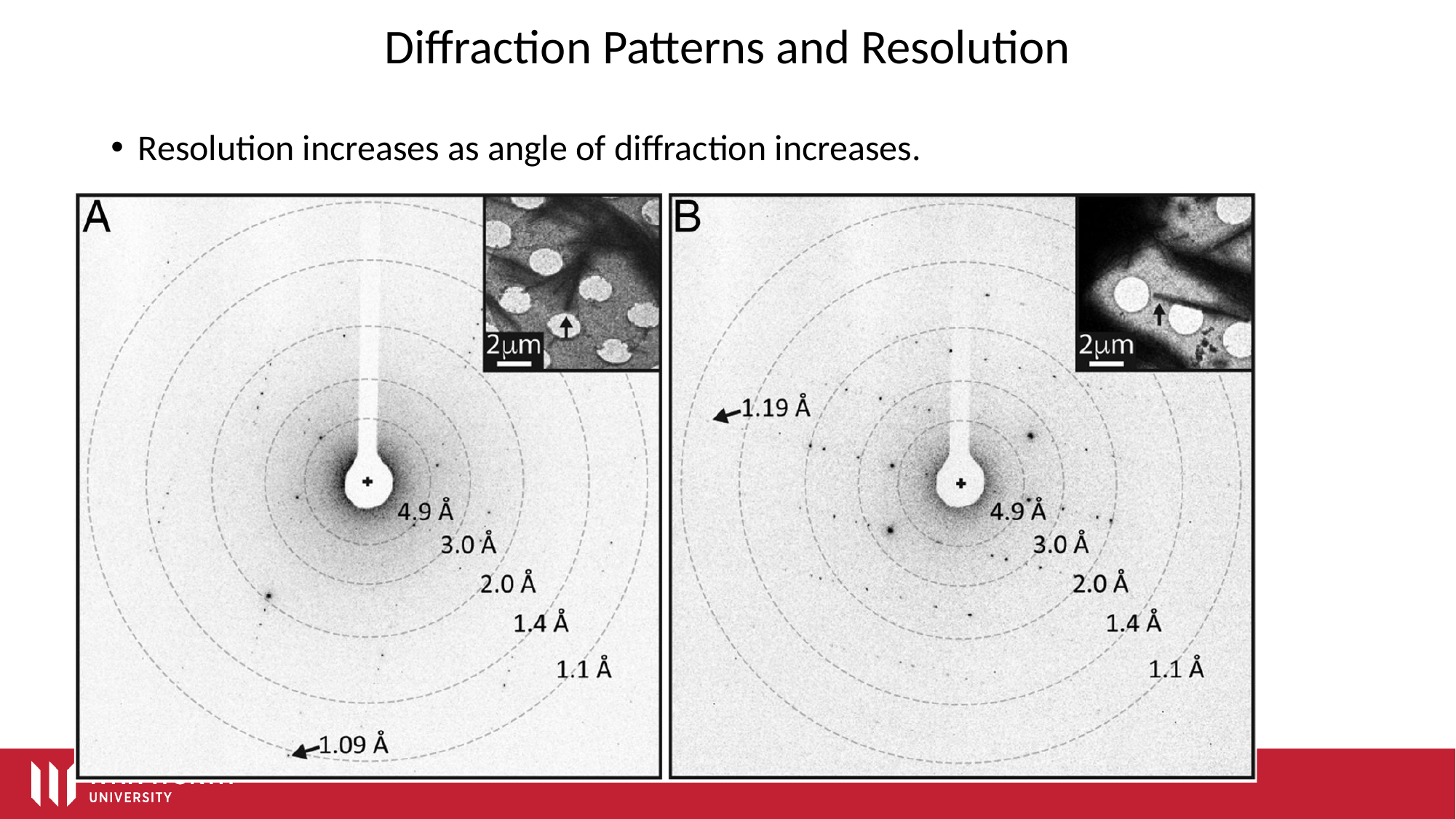

# Diffraction Patterns and Resolution
Resolution increases as angle of diffraction increases.

## Slide 24
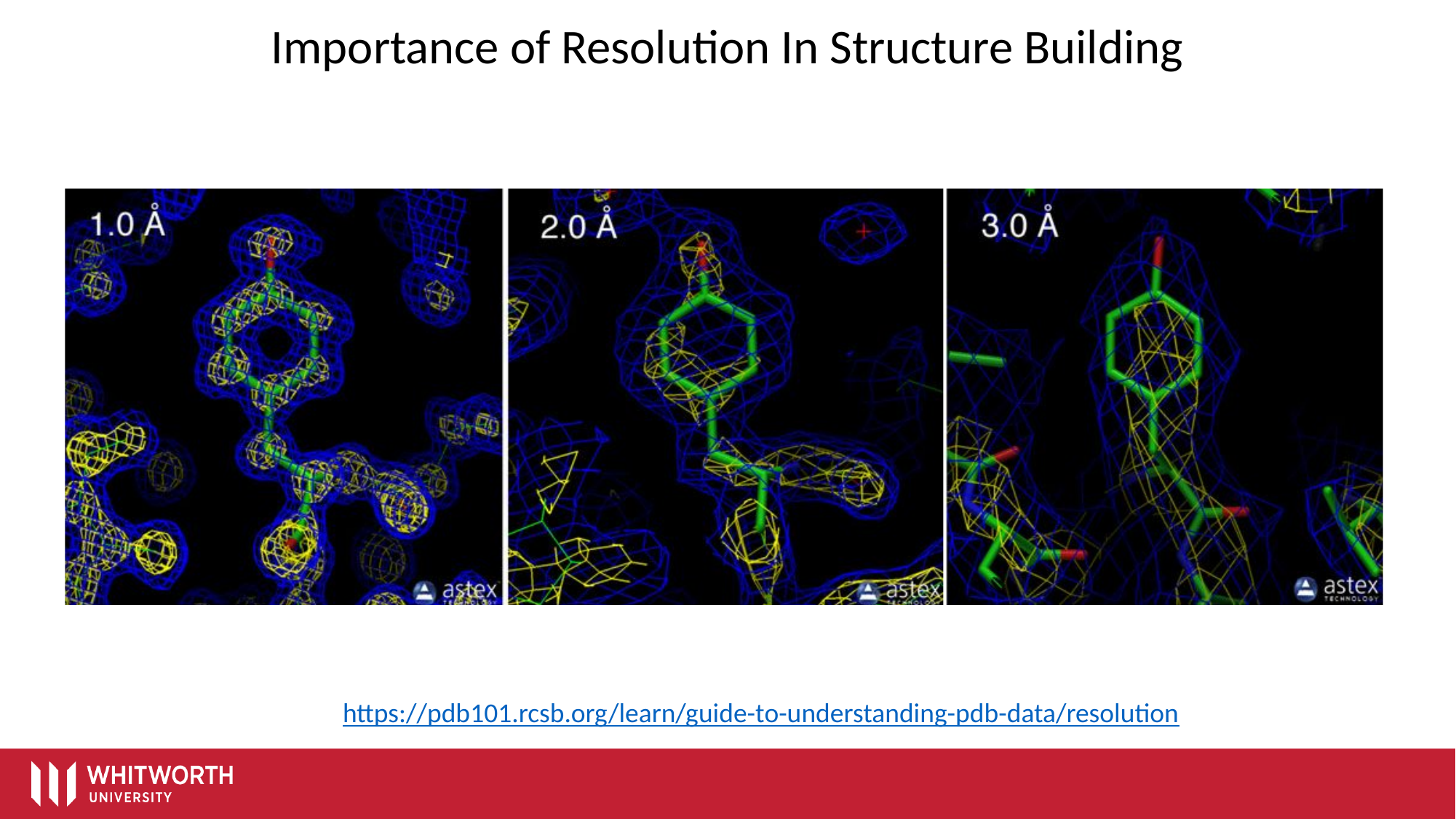

# Importance of Resolution In Structure Building
https://pdb101.rcsb.org/learn/guide-to-understanding-pdb-data/resolution

## Slide 25
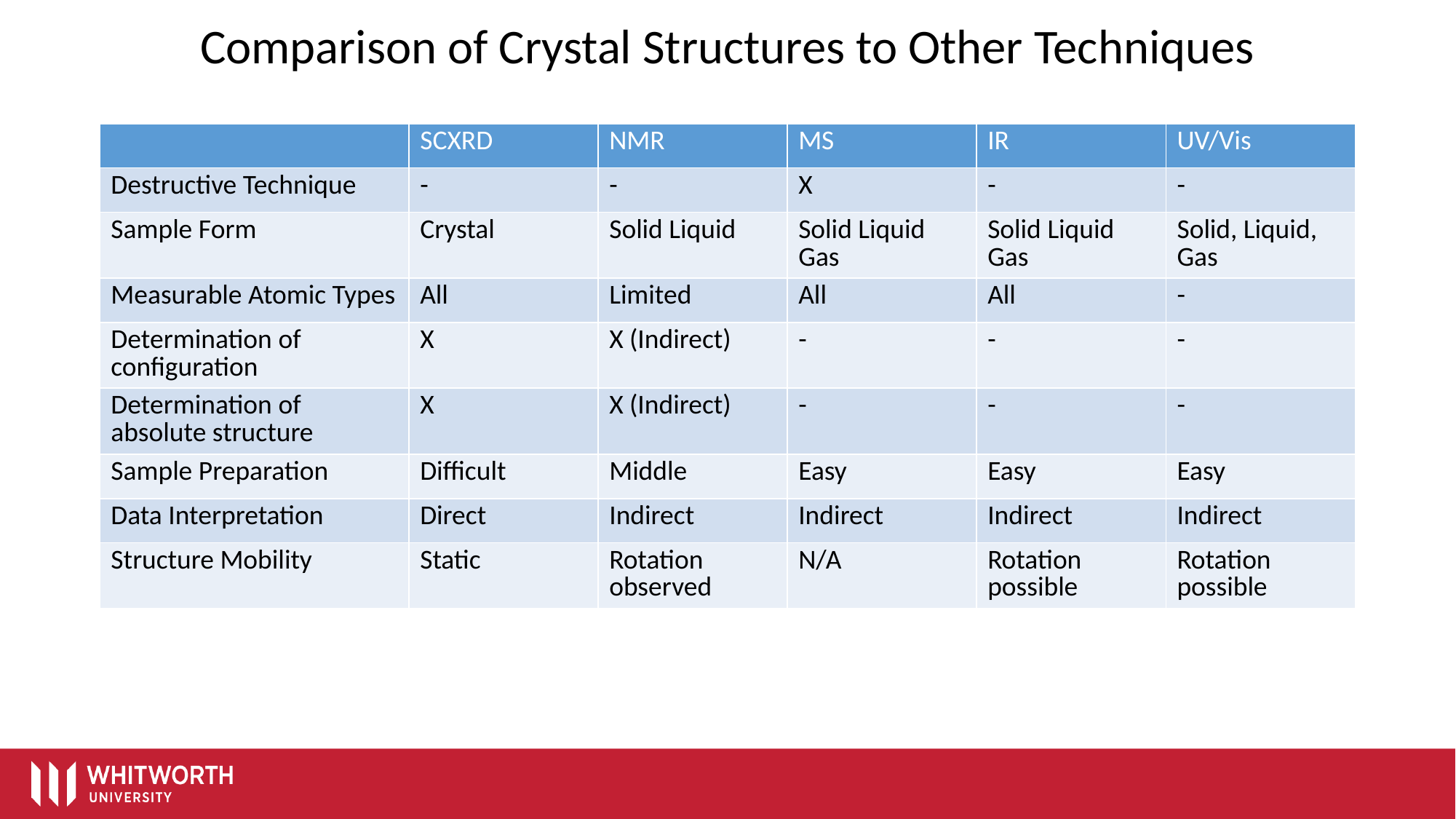

# Comparison of Crystal Structures to Other Techniques
| | SCXRD | NMR | MS | IR | UV/Vis |
| --- | --- | --- | --- | --- | --- |
| Destructive Technique | - | - | X | - | - |
| Sample Form | Crystal | Solid Liquid | Solid Liquid Gas | Solid Liquid Gas | Solid, Liquid, Gas |
| Measurable Atomic Types | All | Limited | All | All | - |
| Determination of configuration | X | X (Indirect) | - | - | - |
| Determination of absolute structure | X | X (Indirect) | - | - | - |
| Sample Preparation | Difficult | Middle | Easy | Easy | Easy |
| Data Interpretation | Direct | Indirect | Indirect | Indirect | Indirect |
| Structure Mobility | Static | Rotation observed | N/A | Rotation possible | Rotation possible |
