# Supplementary material for: Crystallographic workshops – a primer and perspective from Whitworth University’s Summer Crystallography Institute
Source: Acta Crystallogr E Crystallogr Commun. 2026 Feb 3;82(Pt 3):313–9. doi: 10.1107/S2056989026000939 (PMC12961664; doi:10.1107/S2056989026000939)
Supplement: Supplementary file 1 [file e-82-00313-sup2.zip › Lectures/SCI_Structure Solution_Refinement.pptx]

## Slide 1
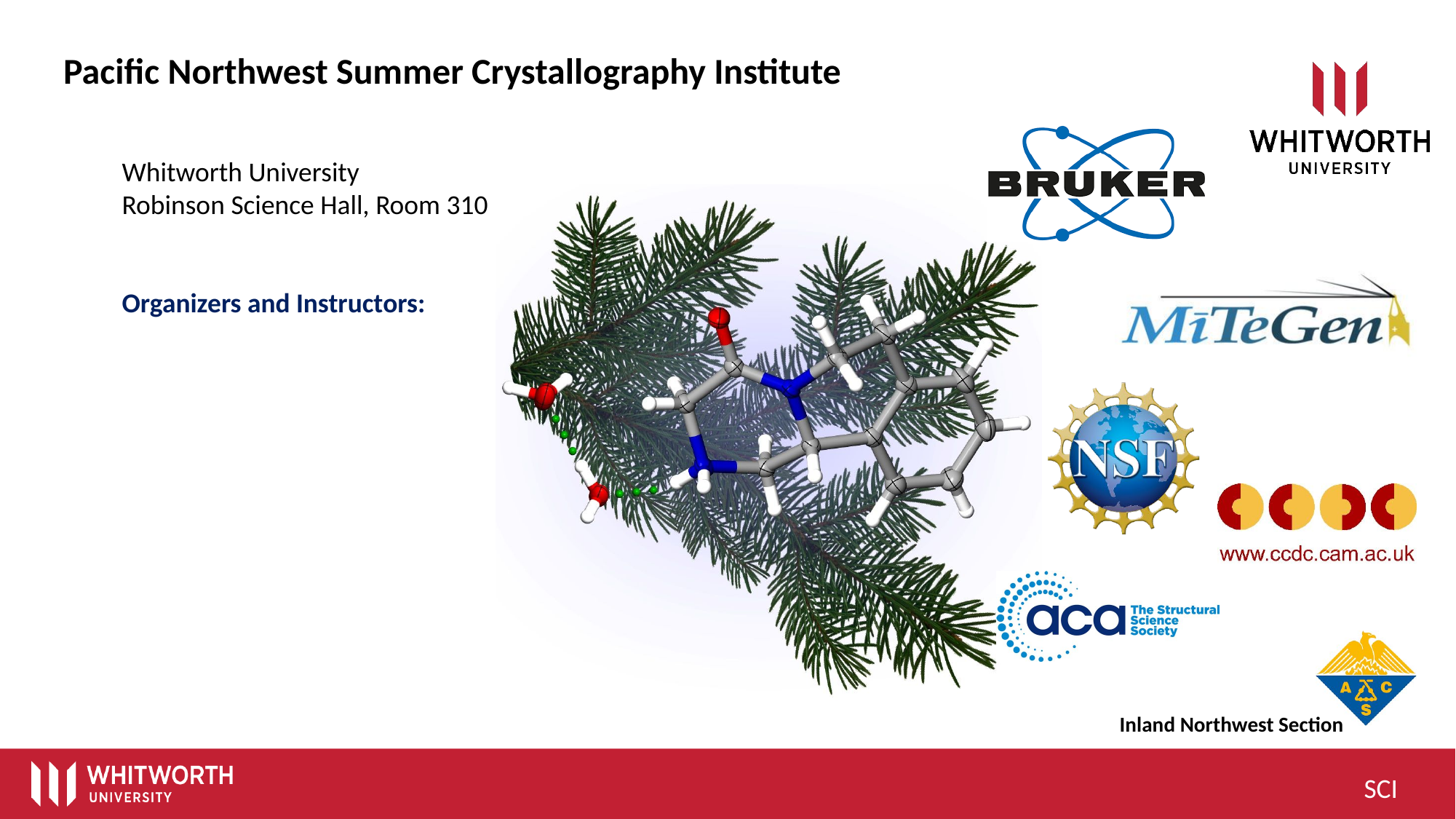

Pacific Northwest Summer Crystallography Institute
Whitworth University
Robinson Science Hall, Room 310
Organizers and Instructors:
Inland Northwest Section
SCI

## Slide 2
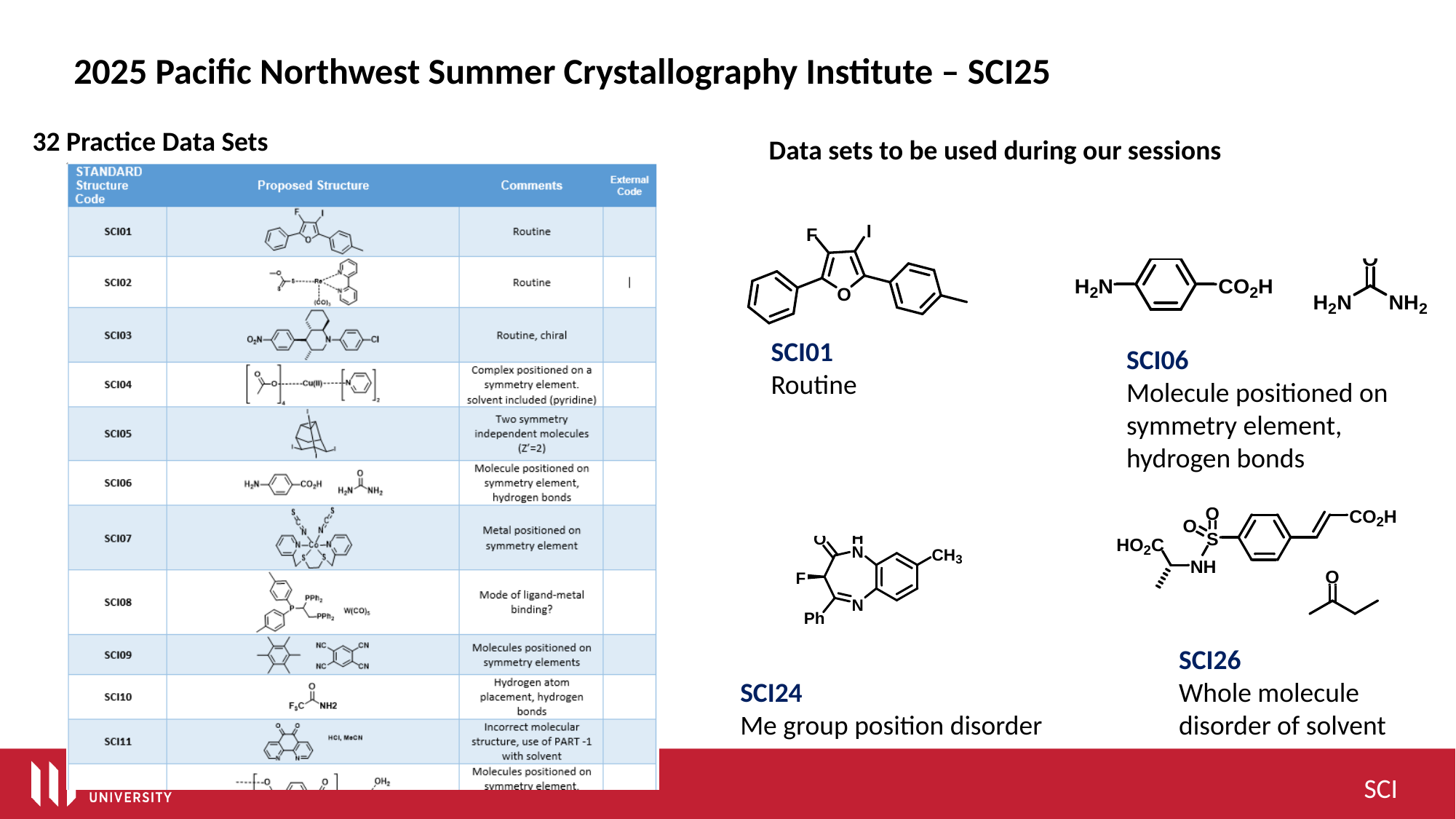

2025 Pacific Northwest Summer Crystallography Institute – SCI25
32 Practice Data Sets
Data sets to be used during our sessions
SCI01
Routine
SCI06
Molecule positioned on symmetry element, hydrogen bonds
SCI26
Whole molecule disorder of solvent
SCI24
Me group position disorder
SCI

## Slide 3
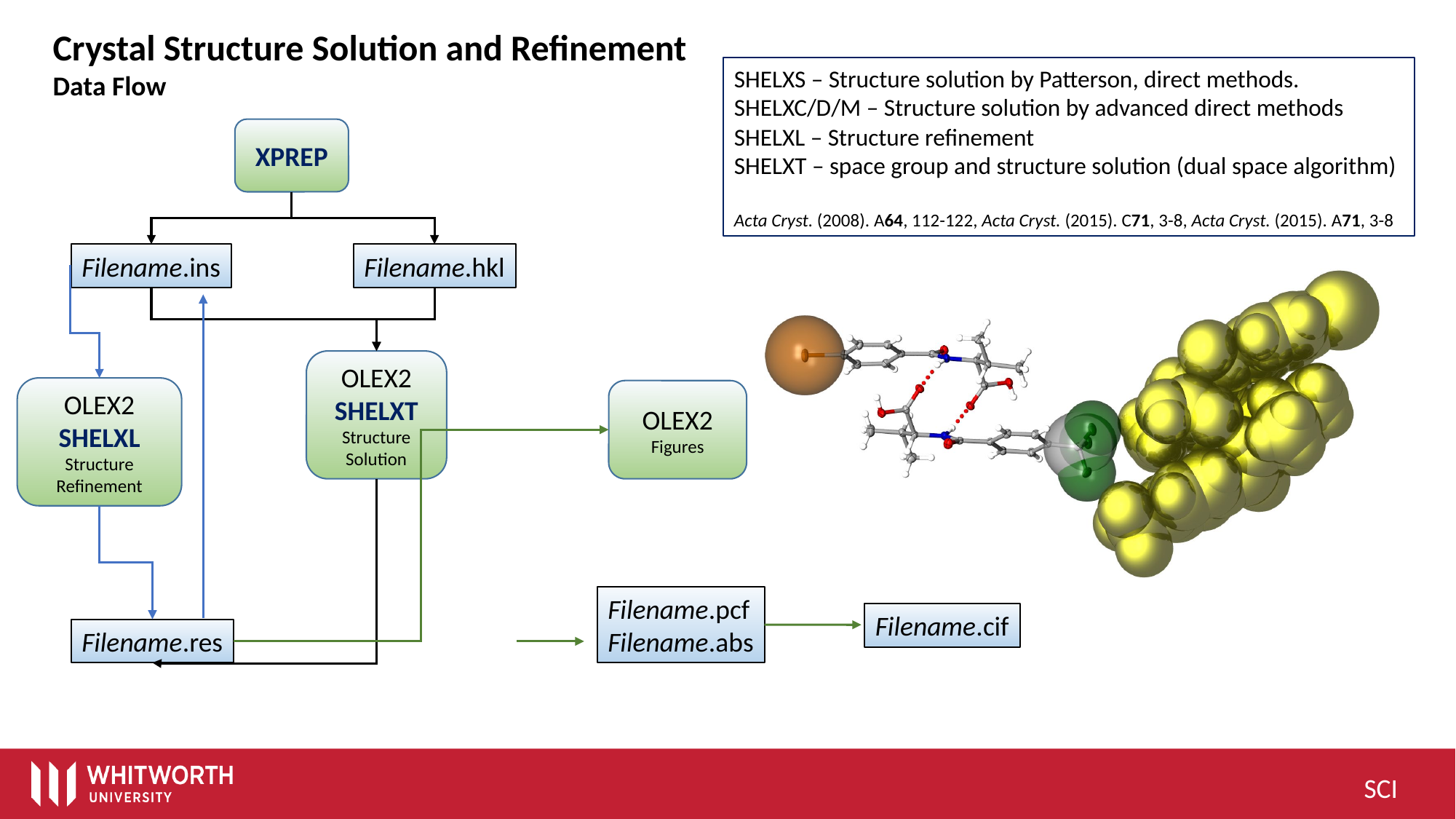

Crystal Structure Solution and Refinement Data Flow
SHELXS – Structure solution by Patterson, direct methods.
SHELXC/D/M – Structure solution by advanced direct methods
SHELXL – Structure refinement
SHELXT – space group and structure solution (dual space algorithm)
Acta Cryst. (2008). A64, 112-122, Acta Cryst. (2015). C71, 3-8, Acta Cryst. (2015). A71, 3-8
XPREP
Filename.ins
Filename.hkl
OLEX2
SHELXT
Structure Solution
OLEX2
SHELXL
Structure Refinement
OLEX2
Figures
Filename.pcf
Filename.abs
Filename.cif
Filename.res
SCI

## Slide 4
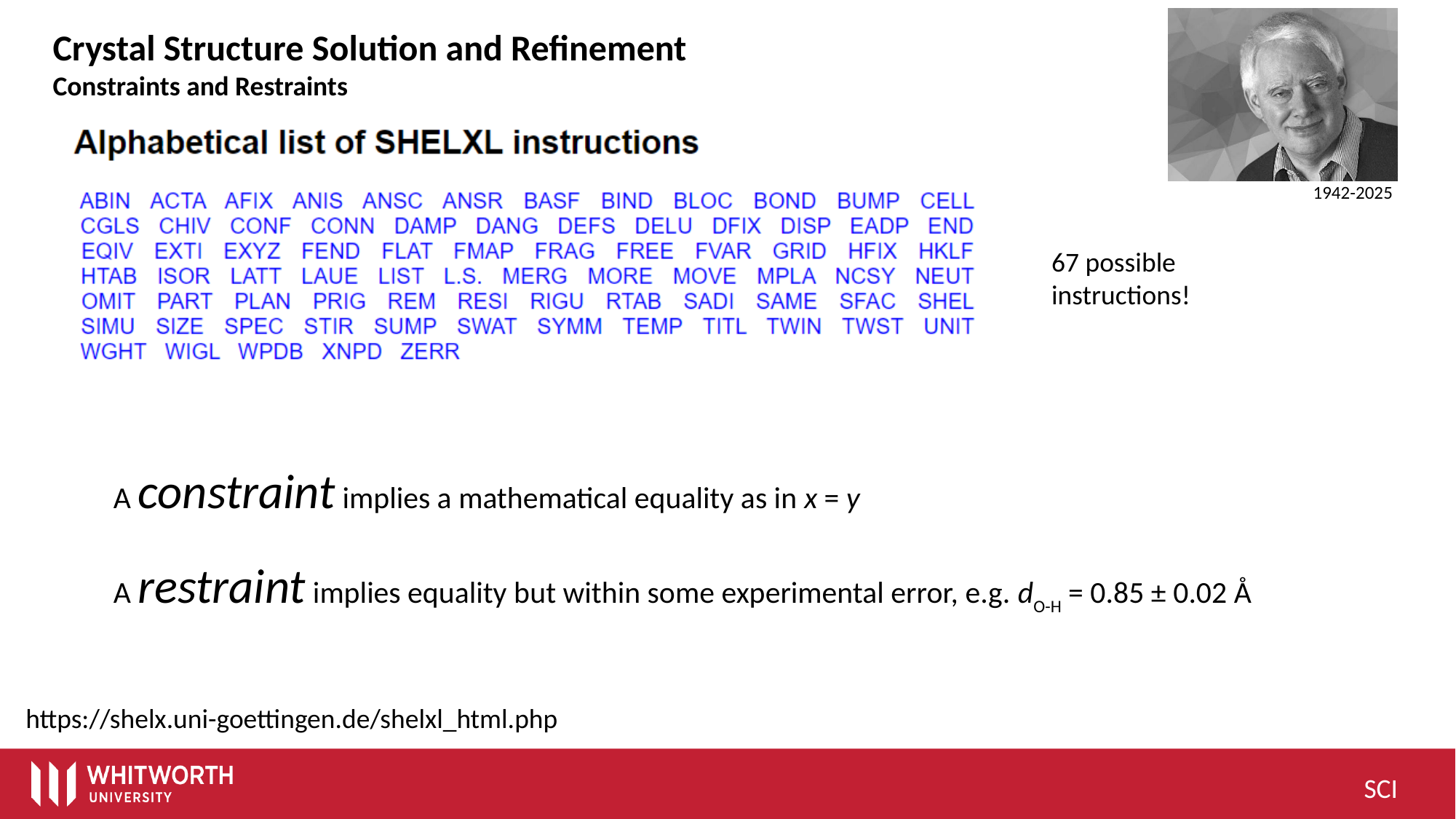

Crystal Structure Solution and Refinement Constraints and Restraints
1942-2025
67 possible instructions!
A constraint implies a mathematical equality as in x = y
A restraint implies equality but within some experimental error, e.g. dO-H = 0.85 ± 0.02 Å
https://shelx.uni-goettingen.de/shelxl_html.php
SCI

## Slide 5
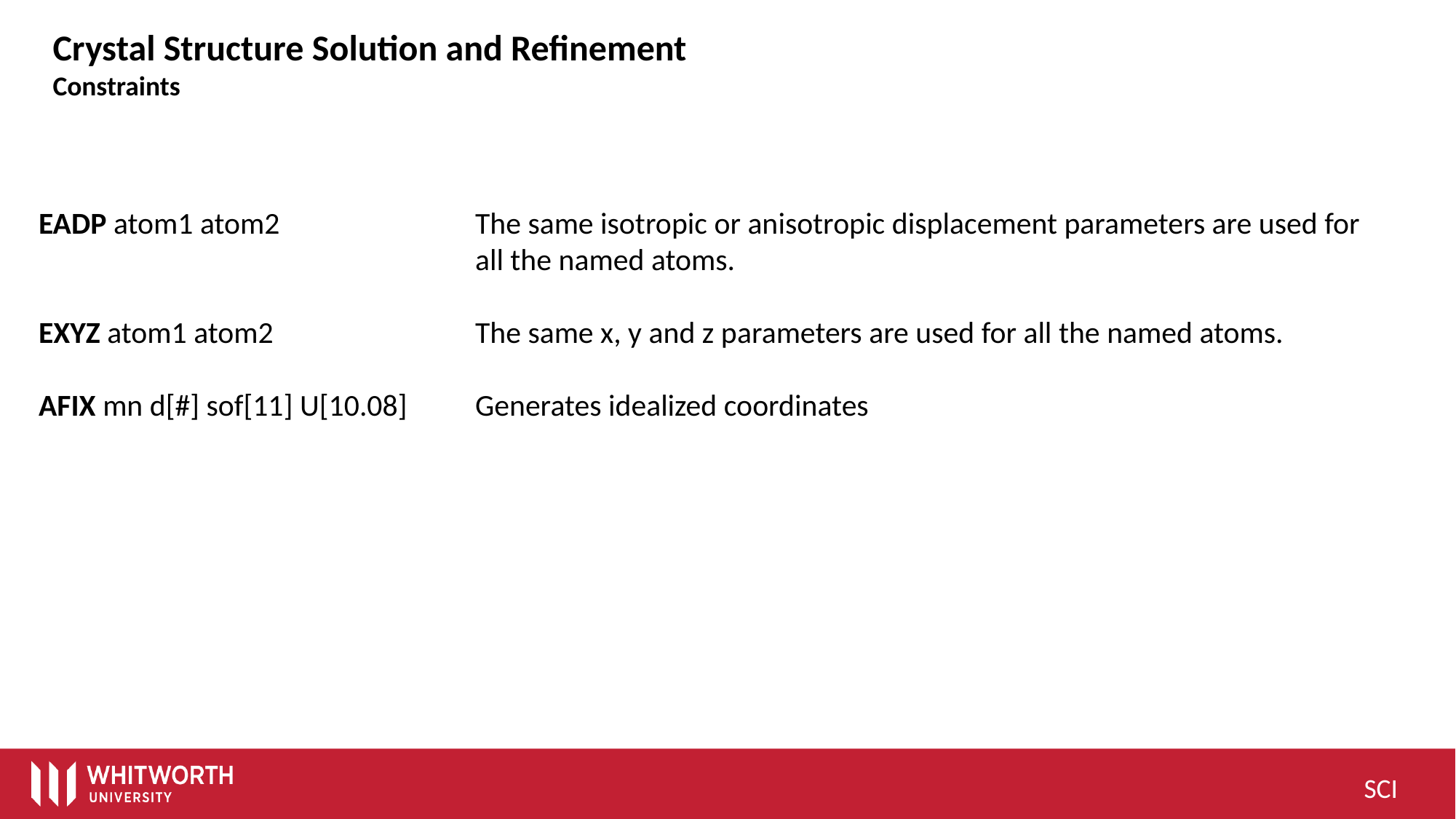

Crystal Structure Solution and Refinement Constraints
EADP atom1 atom2	The same isotropic or anisotropic displacement parameters are used for all the named atoms.
EXYZ atom1 atom2		The same x, y and z parameters are used for all the named atoms.
AFIX mn d[#] sof[11] U[10.08] 	Generates idealized coordinates
SCI

## Slide 6
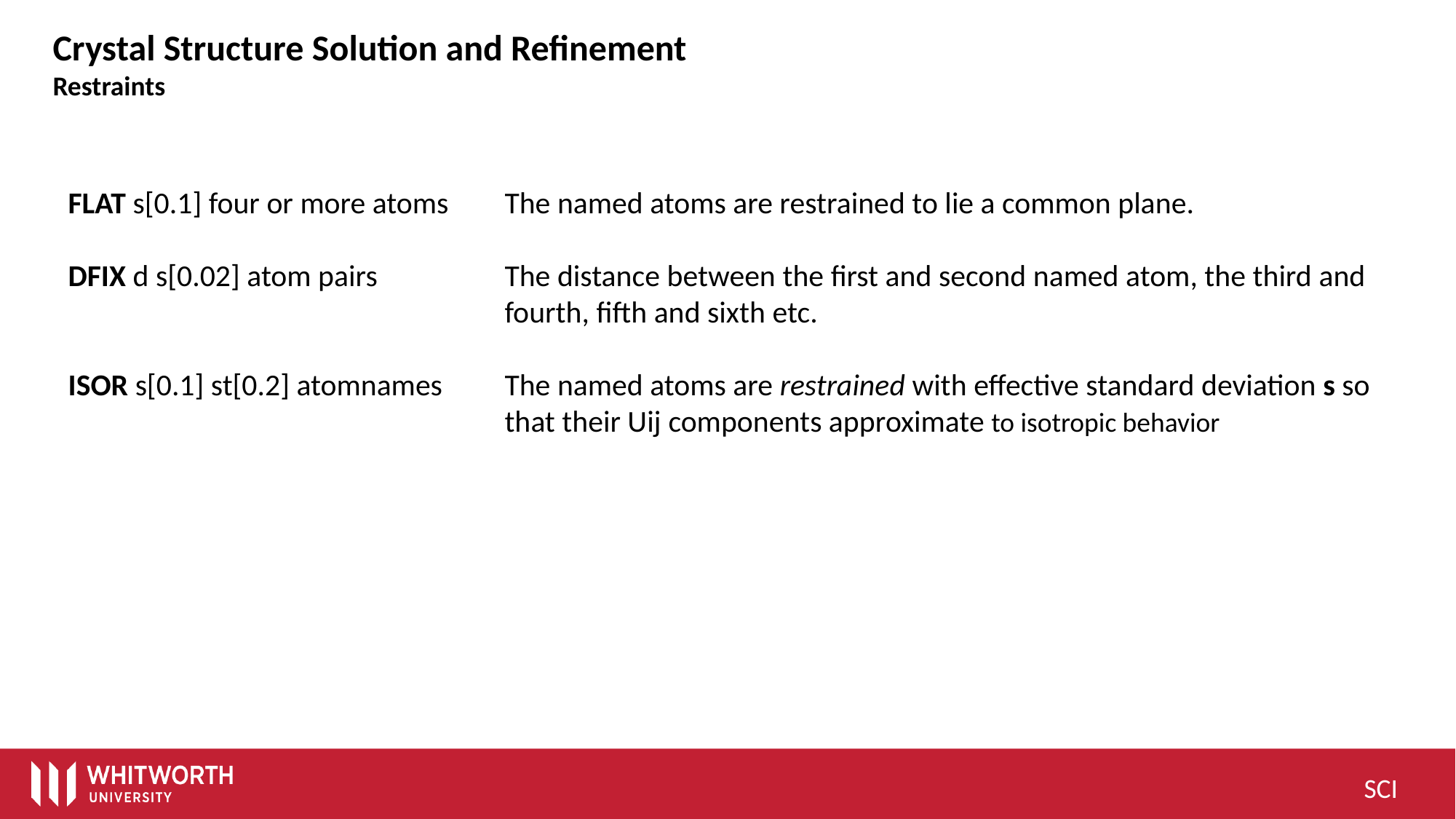

Crystal Structure Solution and Refinement Restraints
FLAT s[0.1] four or more atoms	The named atoms are restrained to lie a common plane.
DFIX d s[0.02] atom pairs	The distance between the first and second named atom, the third and fourth, fifth and sixth etc.
ISOR s[0.1] st[0.2] atomnames	The named atoms are restrained with effective standard deviation s so that their Uij components approximate to isotropic behavior
SCI

## Slide 7
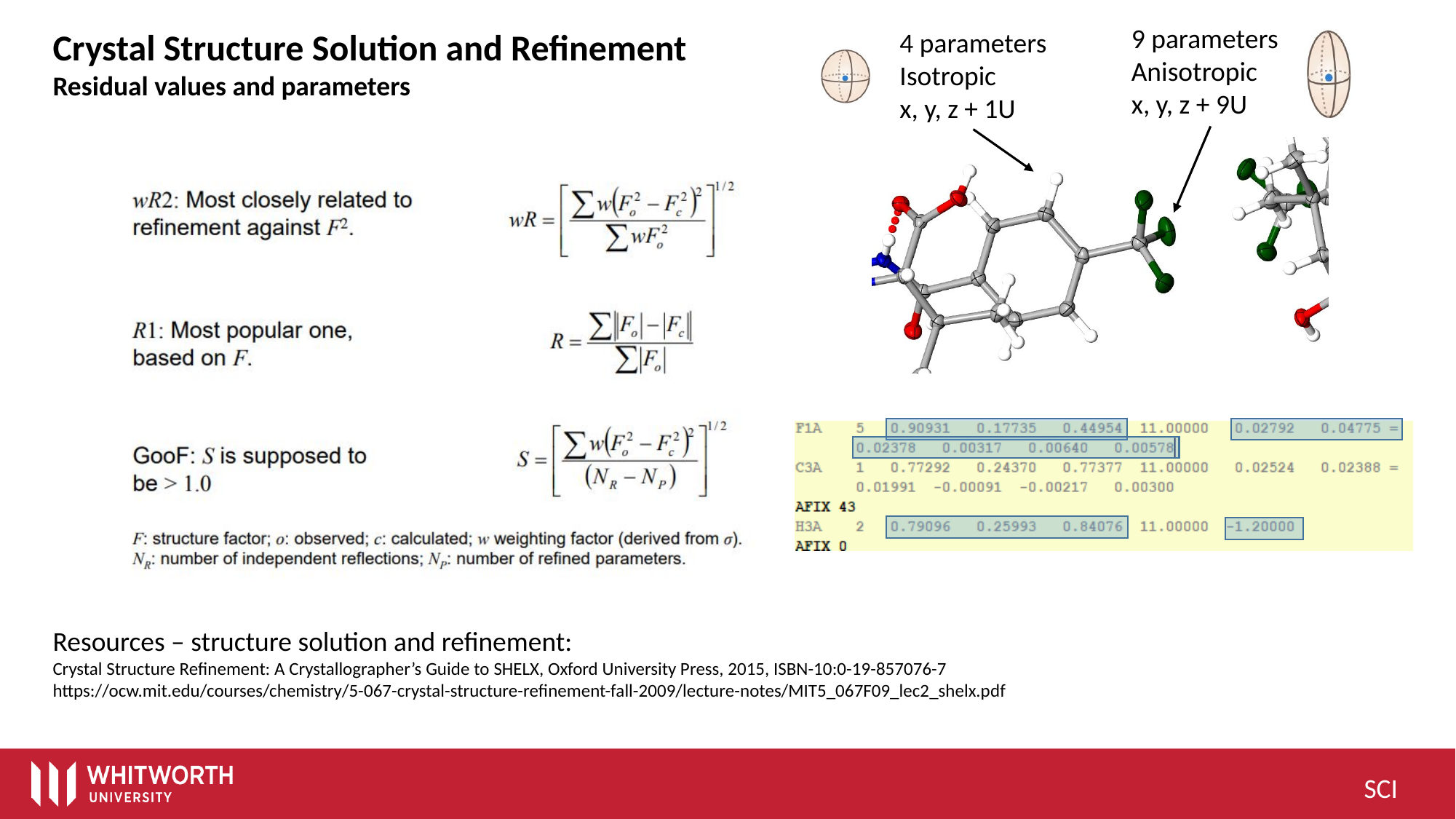

9 parameters
Anisotropic
x, y, z + 9U
Crystal Structure Solution and Refinement Residual values and parameters
4 parameters
Isotropic
x, y, z + 1U
Resources – structure solution and refinement:
Crystal Structure Refinement: A Crystallographer’s Guide to SHELX, Oxford University Press, 2015, ISBN-10:0-19-857076-7
https://ocw.mit.edu/courses/chemistry/5-067-crystal-structure-refinement-fall-2009/lecture-notes/MIT5_067F09_lec2_shelx.pdf
SCI
