# Supplementary material for: Crystallographic workshops – a primer and perspective from Whitworth University’s Summer Crystallography Institute
Source: Acta Crystallogr E Crystallogr Commun. 2026 Feb 3;82(Pt 3):313–9. doi: 10.1107/S2056989026000939 (PMC12961664; doi:10.1107/S2056989026000939)
Supplement: Supplementary file 1 [file e-82-00313-sup2.zip › Practice Data Sets/SCIDataSets_final.pdf]

Pacific Northwest Summer Crystallographic Institute — Whitworth University, Spokane, WA USA

Catalogue of Crystallographic Data Sets

Structure Code	Proposed Structure	Comments
SCI01	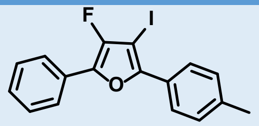	Routine
SCI02	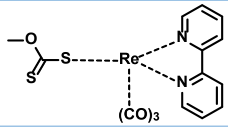	Routine
SCI03	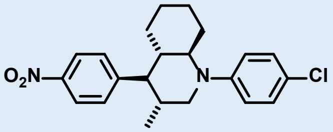	Routine, chiral
SCI04	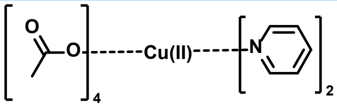	Complex positioned on a symmetry element. solvent included (pyridine)
SCI05	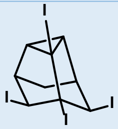	Two symmetry-independent molecules (Z'=2)
SCI06	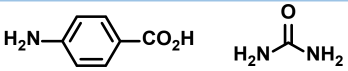	Molecule positioned on a symmetry element, hydrogen bonds
SCI07	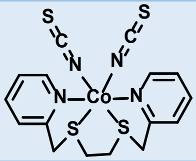	Metal positioned on a symmetry element
SCI08	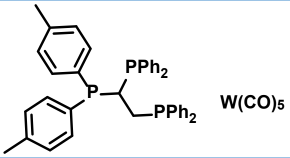	Mode of ligand-metal binding?
SCI09	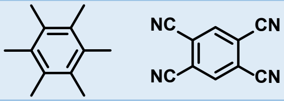	Molecules positioned on symmetry elements
SCI10	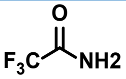	Hydrogen atom placement, hydrogen bonds
SCI11	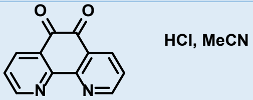	Incorrect molecular structure, use of PART -1 command with solvent
SCI12	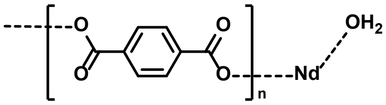	Molecules positioned on a symmetry element, solvent inclusion. Water H atom placement
SCI13	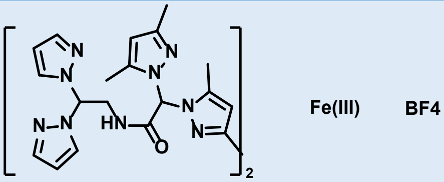	Ligand connectivity?

**SCI14**	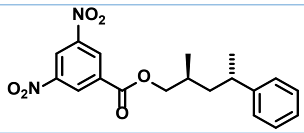	Chiral molecule and space group, no hydrogen bonds
**SCI15**	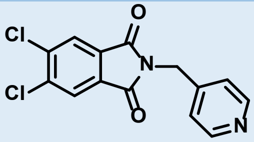	One atom is assigned incorrectly in SHELXT
**SCI16**	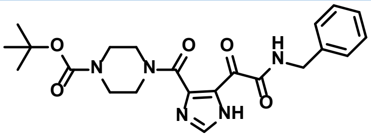	Hydrogen bonds, molecular folding
**SCI17**	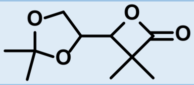	Two symmetry-independent molecules (Z’ = 2)
**SCI18**	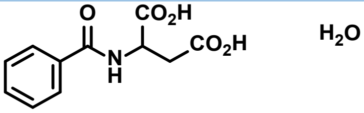	Solvent inclusion, hydrogen bonds
**SCI19**	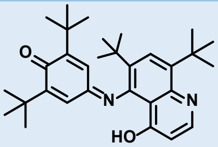	Placement of alcohol H atom, t-Bu group ID
**SCI20**	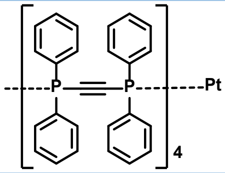	Unexpected structure – bonding pattern and ligand structure

DISORDER Structure Code	Proposed Structure	Comments
**SCI21**	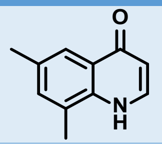	Me group disorder, hydrogen bonds
**SCI22**	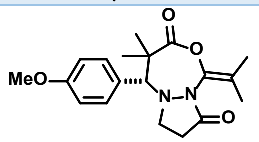	Me group disorder, partial incorrect structure
**SCI23**	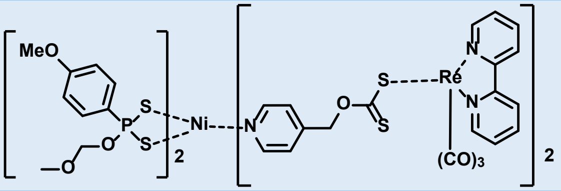	OMe group disorder over two sites. Inclusion of solvent
**SCI24**	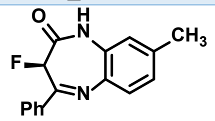	Me group position disorder
**SCI25**	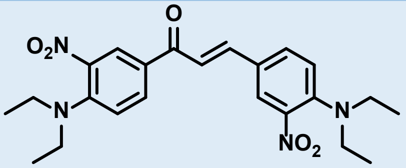	Disorder of C=O and H groups
**SCI26**	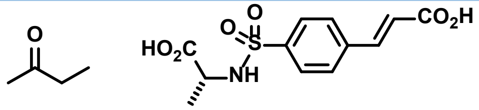	Whole molecule solvent disorder, hydrogen bonds
**SCI27**	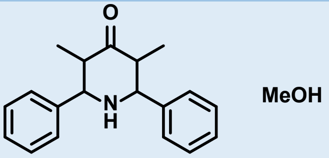	Molecule positioned on symmetry, disordered solvent and C=O group
**SCI28**	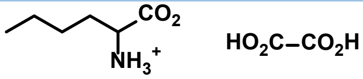	Whole molecular disorder, hydrogen atom position
**SCI29**	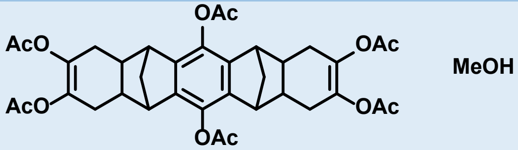	Z' = 2, pendant ester two-part disorder, solvent
**SCI30**	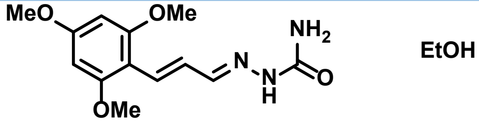	Disordered solvent
**SCI31**	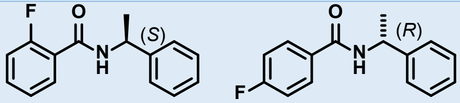	Phenyl and F disorder, use of SUMP command
**SCI32**	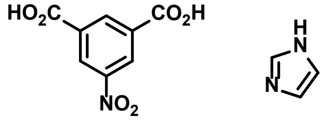	Whole molecule disorder of one component
